# Dynamic Supramolecular Polymers Based on Zinc Bis(diorganophospate)s: Synthesis, Structure and Transformations in Solid State and Solutions

**DOI:** 10.3390/polym14163407

**Published:** 2022-08-20

**Authors:** Maciej Dębowski, Zbigniew Florjańczyk, Katarzyna Godlewska, Alicja Kaczmarczyk, Maciej Dranka, Andrzej Ostrowski

**Affiliations:** Faculty of Chemistry, Warsaw University of Technology, Noakowskiego 3, 00-664 Warsaw, Poland

**Keywords:** hybrid polymer, coordination polymer, epoxide polymerization, metal diorganophosphate, dynamer

## Abstract

The synthesis, structure and some properties of coordination polymers composed of linear zinc bis(diorganophospate)s (ZnDOPs) with a general formula of Zn[O_2_P(OR)_2_]_2_ (where R = CH_3_, C_2_H_5_, *n*-C_4_H_9_, or 2-ethylhexyl group) are described. Hybrid (co)polymers obtained by different procedures were characterized by means of powder XRD, DSC, SEM, TGA coupled with mass spectrometry of the evolved gases and rheological measurements, as well as FTIR and NMR techniques. The morphology, thermal transformations and solubility of ZnDOPs strongly depend on the type of organic substituent in the O_2_P(OR)_2_ ligands and the thermal history of the sample. Because of this, one can obtain highly crystalline rods, semicrystalline powders, as well as rubbery materials exhibiting a second-order transition below −50 °C. Polymeric chains formed by ZnDOPs undergo a reversible dissociation in polar organic solvents (e.g., methanol, DMSO), which allows for easy modification of their composition and physicochemical properties via a simple exchange of diorganophosphate anions. Some of the ZnDOPs were investigated as the latent curing agents for epoxides. On the basis of rheological and DSC studies, it is evident that ZnDOPs catalyze very effectively the cross-linking process within the 130–160 °C temperature range.

## 1. Introduction

**Figure 1:**
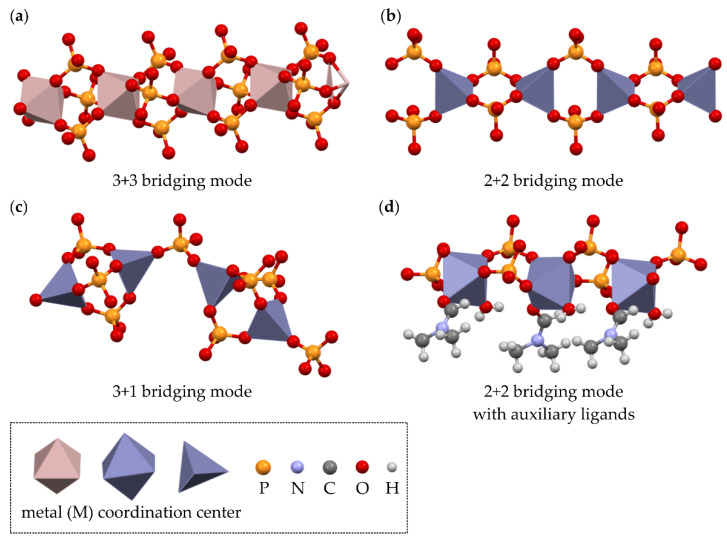
Different modes of metal center binding by (RO)_2_PO_2_^−^ ligands occurring in polymeric metal diorganophosphates: (**a**) triple (RO)_2_PO_2_^−^ bridges, (**b**) double (RO)_2_PO_2_^−^ bridges, (**c**) alternating triple and single (RO)_2_PO_2_^−^ bridges and (**d**) double (RO)_2_PO_2_^−^ bridges with additional ligands. For clarity of presentation organic substituents R are omitted.

For a long time, salts of phosphoric acid diesters have been investigated as synthetic analogues of nucleic acids, lipids and enzymes, as well as precursors of inorganic materials based on metal phosphates [1,2,3,4,5,6,7,8,9]. The studies on their crystal structure have demonstrated that several metal diorganophosphates (MDOPs) exist as one-dimensional (1D) coordination polymers, in which bidentate diorganophospate ligands bridge the adjacent metal cations. Trivalent cations (e.g., Al^3+^, Ce^3+^, Pr^3+^, Nd^3+^) tend to organize into *catena*-like chains containing triple PO_2_ bridges (3 + 3 bridging mode, Figure 1a) and having M(OP)_6_ units as building blocks [10,11,12,13,14,15]. On the other hand, divalent cations (e.g., Ba^2+^, Mg^2+^, Zn^2+^, Cd^2+^) are usually connected via double PO_2_ bridges forming eight-membered P_2_O_4_M_2_ rings [1,10,16,17,18,19,20] (2 + 2 bridging mode, Figure 1b). However, they can also adopt a different mode of polymeric backbone formation in which tetrahedrally coordinated metal centers are alternatively linked together by single and triple diorganophosphate bridges (3 + 1 bridging mode, Figure 1c). The latter mode occurs if bulky *tert*-butyl [4,20] or *p*-OC_6_H_4_OCH_3_ aromatic groups [21] are present within the (RO)_2_PO_2_^−^ ligands. It should be noted that in some systems metal centers can additionally coordinate small molecules (e.g., water, DMF) as auxiliary ligands (Figure 1d) [9,20,22].

MDOPs and their structural analogues with phosphinate bridging ligands have already been successfully utilized in several areas of modern materials science and engineering. For example, these types of 1D coordination polymers have been used as effective flame retardants [23,24,25,26,27,28], reinforcing fillers in polymer composites [14,23,24,25,28,29], organogelators [30,31,32], as well as components of smart materials that change their properties under the influence of external stimulants (e.g., magnetic or electric field) [21,33,34]. One of the main obstacles to the broader use of MDOPs as materials for new technologies is the lack of information about changes in their molecular structure and morphology under the influence of temperature, solvents or other factors that may affect the durability of bridging bonds between metal centers.

Recently, we have reported on thermally induced transformations in the structure of aluminum tris(diorganophosphate)s (AlDOPs) occurring in the solid state between −100 and 800 °C [35]. We have found that AlDOPs preserve their polymeric structures at least up to 180–200 °C. However, most of them are conformationally labile, and a structural disorder is induced in organic ligands by the rotations around the P–O, O–C and C–C bonds at a sufficiently high temperature. AlDOPs are insoluble in organic solvents, whereas under the influence of primary amines (or water—in the case of AlDOPs containing methyl or ethyl groups), their chains dissociate into diorganophosphate anions and complex aluminum cations [15]. This process is reversible, and the polymeric structure is recovered if the amine is gradually removed by distillation or consumed in the chemical reaction. For example, curing epoxy resins with solutions of AlDOPs in aliphatic polyamine leads to the formation of nanocomposites in which nanocrystals of hybrid polymer are dispersed in a cross-linked polymer matrix [15]. In these systems, AlDOPs behave like dynamers, i.e., supramolecular polymers that can alter their molecular structure via dynamic bond cleavage and reassembly [36,37]. It can be expected that the dynamic feature of MDOPs should enable the exchange of monomeric units in solution and generate supramolecular copolymers whose physicochemical properties can be controlled by the appropriate selection of diorganophosphate anions and their distribution in the polymer backbone.

In our search for new materials showing both high motional dynamics in the solid state and constitutional dynamics in solution, we decided to study structural changes in MDOPs based on divalent metal cations in detail. Herein, we describe the synthesis and characterization of four aliphatic zinc bis(diorganophosphate)s (ZnDOPs) with the general formula of Zn[O_2_P(OR)_2_]_2_ (where R = CH_3_ (ZnDMP), C_2_H_5_ (ZnDEP), *n*-C_3_H_7_ (ZnDnPP), *n*-C_4_H_9_ (ZnDBP), or 2-ethylhexyl (ZnBEHP) groups), as well as several hybrid copolymers bearing two different diorganophosphate ligands in a single polymeric chain (C-ZnDOPs). We also discuss their structures, morphologies and thermal transitions occurring in the solid state, as well as the ability of such type of inorganic-organic hybrid polymers to make dynamic structural changes in solution. Some preliminary findings on the application of ZnDOPs as latent curing agents of epoxy resins are also presented.

## 2. Materials and Methods

### 2.1. Materials

All chemicals for the syntheses were purchased from commercial sources and were used without further purification: trimethyl phosphate (TMP) (97%, Sigma-Aldrich, Burlington, MA, USA), triethyl phosphate (TEP) (99%, Sigma-Aldrich), tri-*n*-propyl phosphate (TnPP) (99%, Sigma-Aldrich), triphenyl phosphate (TPhP) (99%, Merck Schuchardt OHG, Hohenbrunn, Germany), di-*n*-butyl phosphate (DBP) (97%, Sigma-Aldrich), bis(2-ethylhexyl) phosphate (BEHP) (97%, Sigma-Aldrich), zinc oxide (≥99.0%, Merck KGaA, Darmstadt, Germany), zinc acetate dihydrate (ZnOAc·2H_2_O) (99%, Merck KGaA), calcium chloride ≥ 97.0%, Sigma-Aldrich), sodium bicarbonate (pure, POCh S.A., Gliwice, Poland), Bisphenol A diglycidyl ether (BADGE) (Brookfield viscosity of 5279 mPa·s at 25 °C, Sigma-Aldrich).

### 2.2. Methods of Characterization

#### 2.2.1. Elemental Analysis

The hydrogen and carbon contents were determined using a Euro EA elemental analyser made by EuroVector Instruments & Software (Pavia, Italy).

The zinc content was analyzed by means of Flame Atomic Absorption Spectroscopy using a PerkinElmer (Waltham, MA, USA) AAnalyst 300 or AAnalyst 800 instruments (with atomization in an air-acetylene flame). A microwave-assisted sample preparation procedure was utilized, in which, before atomization, the samples were mineralized in nitric acid using a Milestone UltraWAVE digestion system.

#### 2.2.2. Powder XRD (PXRD) Analysis

PXRD patterns were recorded at room temperature on a Bruker (Billerica, MA, USA) D8 Advance automated diffractometer equipped with a Lynx-Eye position-sensitive detector using Cu-Kα radiation (*λ* = 1.5406 Å). The data were collected in the Bragg–Brentano (*θ*/2*θ*) horizontal geometry (flat reflection mode) between the 2*θ* angles of 3° and 60° in steps of 0.03°, with 10 s per step.

#### 2.2.3. Variable Temperature PXRD (VT–PXRD) Analysis

VT-PXRD measurements above room temperature were carried out on a Bruker (Billerica, MA, USA) D8 Discover instrument equipped with a VANTEC–1 position-sensitive detector and an Anton Paar DCS-350 heating stage (temperature stability of 1 K) using Cu-Kα radiation and a step size of 0.0183°. The data were collected between the 2*θ* angles of 4° and 60°.

VT-PXRD measurements below room temperature were carried out on a Bruker AXS (Madison, WI, USA) D8 Advance diffractometer equipped with a silver anode X-ray tube. The beam was monochromatized using a focusing multilayer mirror to select the *K*_α1_–*K*_α2_ doublet with wavelength 0.0561 nm and detected with LynxEye position sensitive detector. The samples were enclosed in a 0.7 mm diameter glass capillary with 10 micrometer thick walls. The samples were cooled using a Cryostream 700 coldhead from Oxford Cryosystems (Oxford, United Kingdom), while the flow of the cooling gas was directed along the capillary axis.

#### 2.2.4. Fourier-Transform Infrared (FTIR) Spectroscopy

FTIR spectra were recorded on a Thermo Scientific Nicolet iS5 spectrometer (Waltham, MA, USA) equipped with an iD7 diamond attenuated total reflectance accessory. All sample or background spectra consisted of 16 scans (1.6 s per scan).

Figure S1 in the Supplementary Materials (SM) contains FTIR spectra of the synthesized ZnDOPs.

#### 2.2.5. NMR Spectroscopy in Solution

^1^H and ^31^P NMR spectra were recorded in perdeuterated solvents on a Varian (Palo Alto, CA, USA) NMR System 500 MHz spectrometer operating at 499.87 and 202.35 MHz, respectively. Chemical shifts are reported relative to the residual solvent signal (^1^H) and 85% H_3_PO_4(aq)_ (^31^P). The measurements were carried out at room temperature.

Figures S2–S6 in SM contain ^1^H and ^31^P NMR spectra recorded for the solutions of the investigated ZnDOPs in perdeuterated solvents (DMSO-d_6_ or CDCl_3_).

#### 2.2.6. Diffusion-Ordered NMR Spectroscopy (DOSY NMR)

^1^H DOSY NMR spectra were recorded in perdeuterated solvents on a Varian (Palo Alto, CA, USA) NMR System 500 MHz spectrometer operating at 499.87. Chemical shifts are reported relative to the residual solvent signal. The measurements were carried out at room temperature. Each ^1^H DOSY NMR measurement consisted of a series of 32 spectra: the number of scans 16, acquisition time 4s, relaxation pause d_1_ = 2s—gradient power and delta time were selected in such a way as to record the decay of each of the tested signals as fully as possible.

#### 2.2.7. Differential Scanning Calorimetry (DSC)

DSC measurements were conducted on a TA Instruments (New Castle, DE, USA) DSC Q200 apparatus. Unless stated otherwise, the following temperature program was applied: heating run from −150 to 220 °C followed by a cooling run to −150 °C and another heating run from −150 to 220 °C. During all heating/cooling runs, the temperature was changed at a steady rate of 10 °C/min.

#### 2.2.8. Simultaneous Thermal Analysis (STA)

Thermal degradation under an inert atmosphere, volatile thermal decomposition products and thermal effects of the processes occurring during heating were studied by thermogravimetry (TG) coupled with differential thermal analysis (DTA) and quadrupole mass spectrometry (QMS) using a Netzsch-Gerätebau GmbH (Selb, Germany) STA 449 C Jupiter apparatus coupled with a Netzsch-Gerätebau GmbH (Selb, Germany) QMS 403C Aeolos quadrupole mass spectrometer. The samples were placed in Al_2_O_3_ crucibles and heated in a stream of argon (flow rate of 90 mL/min) from 30 to 1000 °C at a heating rate of 5 °C/min.

#### 2.2.9. Scanning Electron Microscopy (SEM)

SEM images were obtained using a Carl Zeiss AG (Oberkochen, Germany) Ultra Plus field-emission scanning electron microscope equipped with a GEMINI column. Before the imaging, all samples were coated with an electron-conductive layer of carbon or Au/Pd alloy utilizing a high-vacuum sputter coater.

Low-magnification SEM images of ZnDMP and ZnDEP were recorded on a Prisma E microscope (Thermo Fisher Scientific Co., Waltham, MA, USA) working in a low vacuum mode, with no preparation of those samples.

#### 2.2.10. Melting Temperature Analysis

The behavior of samples during heating was visually observed on a Gallenkamp (Cambridge, UK) variable heater, model MPD350.BM2.5. Each sample was placed in a glass capillary tube (outer diameter 1.35 mm and sample height ca. 2 mm).

#### 2.2.11. Rheological Studies

Rheological measurements were conducted on an Anton Paar (Graz, Austria) Physica MCR 301 rotational rheometer using a parallel plate measuring system equipped with the P–PTD/200/TG heating cell and disposable D-PP25/AL/S07 plate (25 mm diameter, measuring gap of 1 mm). The measurements were carried out in an oscillation mode (strain 2%, frequency 10 rad/s), with a continuous stabilization of normal force (FN) at 0 N. Temperature was changed from 80 to 190 °C, with a heating rate of 2 °C/min.

The samples subjected to the analysis were taken from the respective dispersions of ZnDOPs in BADGE that had been prepared earlier at room temperature by mechanical mixing of their constituents in an agate mortar.

#### 2.2.12. Viscometry

The relative viscosity of the diluted solutions of ZnDOPs was measured at 30 °C on an automatic AVS 370 viscometer (SI Analytics, Mainz, Germany) equipped in a SI Analytics 0a Ubbelohde type capillary (capillary constant of 0.0049790 mm^2^/s). Each solution was prepared in the 25 mL volumetric flask either by dissolving a specified amount of ZnDOP in a selected solvent, or by a dilution of the previously prepared stock solution. Before the measurements, each ZnDOP solution was conditioned for 30 min at 30 °C. The relative viscosity was calculated as a mean from 4 consecutive measurements that did not differ more than 1%—the flow times of the solutions were compared with those of the pure solvents.

### 2.3. Preparation of ZnDOPs

#### 2.3.1. Reaction of ZnO with Phosphoric Acid Triester (TOP)

In the case of water-miscible TMP or TEP, the respective ZnDOPs were synthesized according to the following procedure: ZnO (20.00 g, 243.3 mmol), TOP (511 mmol) and redistilled water (550 mL) were placed in a 1 L round-bottom flask equipped with magnetic stirring bar and reflux condenser. The mixture was vigorously stirred and heated under reflux for 24 h. After the reaction completion and cooling to room temperature, the post-reaction mixture was filtered off. The clear and colorless filtrate was concentrated on a rotary evaporator at 60 °C, resulting in the formation of a large amount of white crystalline solid. After dispersing in 100 mL of ethyl ether, the crude insoluble product was isolated via filtration on a Büchner funnel and washed with Et_2_O (4 × 50 mL). The final product was obtained after drying at 60 °C in a vacuum oven. In this manner, ZnDMP (73.82 g, reaction yield of 96%) or ZnDEP (52.28 g, reaction yield of 58%) was synthesized.

#### 2.3.2. Reaction of ZnO with Di-n-propyl phosphate

Di-*n*-propyl phosphate (DnPP) was synthesized from TnPP and CaCl_2_ according to the procedure described in SM.

A solution of DnPP (62.04 g, 340.6 mmol) in 50 mL of diethyl ether was slowly added to a dispersion of ZnO (14.00 g, 170.3 mmol) in 100 mL of methanol, and the resulting mixture was vigorously mixed for 2 days at room temperature. Afterwards, the mixture was heated up to a boiling point, cooled to room temperature and separated via centrifugation. The insoluble solid was washed several times with methanol and the combined methanol layers were evaporated resulting in the formation of crude ZnDnPP. The final product was dried in a vacuum oven at 40 °C.

#### 2.3.3. Reaction of ZnOAc·2H_2_O with Triesters of H_3_PO_4_

- Synthesis of Zinc Bis(diphenylphosphate) (ZnDPhP)

ZnDPhP was obtained according to the method described in the literature (for details see Ref. [33], TPhP Method).

- Synthesis of Zinc Bis(di-*n*-butylphosphate) (ZnDBP)

Into a 100 mL stainless steel pressure reactor (Serie 4790, Parr Instrument Company, Moline, IL, USA) equipped with a PTFE liner and magnetic stirring bar, a solution of ZnOAc·2H_2_O (3.00 g, 13.5 mmol) in 10 mL of water and TBP (7.88 g, 28.7 mmol) were placed. After closing, the reactor was heated for 48 h in an oil bath (*T* = 150 °C). After cooling to room temperature, the organic liquid phase was separated from the aqueous one and subjected to a prolonged vacuum distillation at 160 °C (*p* ≈ 5 × 10^−2^ mbar) resulting in the formation of 2.23 g of the solid product (reaction yield of 34%).

#### 2.3.4. Reactions of ZnOAc·2H_2_O with Phosphoric Acid Diester Sodium Salt

- Standard Method

A typical synthesis was carried out according to the following procedure. In a beaker equipped with a magnetic stirrer, DBP (41.42 g, 191.1 mmol) was neutralized with a 5 wt% aqueous solution of sodium bicarbonate (16.05 g, 191.0 mmol). A solution of ZnOAc·2H_2_O (20.0 g, 90.2 mmol) in water (200 mL) was added dropwise under vigorous stirring to the resulting clear solution of sodium salt of DBP. Even at room temperature, the ion-exchange reaction proceeded very quickly resulting in the precipitation of a flocculent white solid. After 4 h of a vigorous mixing at room temperature, the beaker was placed in a water bath heated to 60 °C and kept there for an additional 1 h. The resulting large agglomerates of an insoluble product were filtered off, and the crude insoluble product was purified by washing several times with distilled water. The final product (ZnDBP) was obtained after 2 days of drying in a vacuum oven at 60 °C (27.49 g of a white powder, reaction yield of 63%).

ZnBEHP was obtained from 8.06 g (95.9 mmol) NaHCO_3_, 31.80 g (95.7 mmol) BEHP and 10.00 g (45.6 mmol) ZnOAc·2H_2_O. The reaction conditions and solution concentrations, as well as isolation and purification of the product were the same as in the case of ZnDBP, although with some minor exceptions. For example, after the neutralization of BEHP with NaHCO_3_ solution, a white dispersion was obtained rather than a clear, colorless solution formed during ZnDBP synthesis; there also occurred no coalescence of solid particles during the heating step. After 2 days of drying in a vacuum oven at 60 °C, a semi-translucent white, waxy solid (ZnBEHP, 30.34 g, reaction yield of 95%) was obtained.

- Room Temperature Method

In contrast to the high temperature method, zinc salts of DBP or BEHP were also obtained without application of heating at the second stage of the process. This procedure resulted in the formation of a white solid (RT-ZnDBP: 12.66 g, reaction yield of 29%, or RT-ZnBEHP: 30.50 g, reaction yield of 95%), after vacuum-drying at 25 °C.

#### 2.3.5. Synthesis of Hybrid Copolymers Based on Zinc Bis(diorganophosphate)s (C-ZnDOPs)

Randomization of monomeric units derived from the pre-synthesized two different ZnDOPs was carried out according to the following procedure: a known amount of each hybrid homopolymer was dissolved at room temperature in methanol (or a 1:1 *v/v* mixture of methanol and chloroform in the case of C-ZnDOPs containing ligands derived from BEHP) to give clear solutions with concentration below 2 wt%. The proper amounts of each solution (calculated based on the chosen molar ratio of monomeric units in the resulting C-ZnDOPs) were weighed into a single beaker and vigorously mixed for 1 h at room temperature. Afterwards, the mixture was concentrated on a rotary evaporator at 60 °C and the resulting solid residue was additionally dried in a vacuum oven at 40 °C, giving the final product.

#### 2.3.6. Curing of BADGE Utilizing Zinc Bis(diorganophosphate) (ZnDOP) as Catalyst

The general procedure was as follows. A mixture of BADGE (2.00 g) and the respective ZnDOP (catalyst loading of 20 or 50 wt%) was thoroughly ground in an agate mortar at room temperature. After being transferred on a PTFE evaporating dish, the resulting dispersion was placed in an oven and conditioned at the selected temperature (e.g., 130 or 160 °C) until it solidified. Afterwards, it was cooled to room temperature and pulverized in an agate mortar.

## 3. Results

### 3.1. Synthesis and Morphology of ZnDOPs

**Scheme 1:**
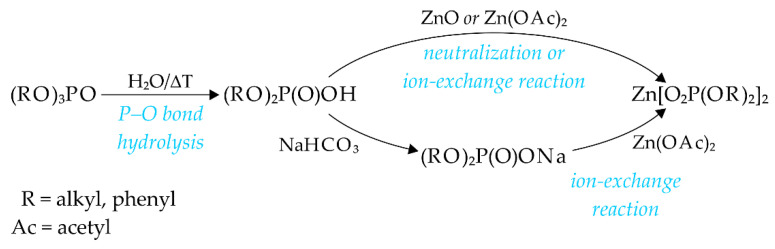
Reaction pathways to ZnDOPs applied in the present study.

Two major synthetic routes were employed for the ZnDOPs synthesis, as shown on Scheme 1. One of them was based on the one-pot, hydrothermal method previously described by Harrison and coworkers [16,18]; in our studies, we modified it by shortening the time necessary for its completion and applying milder conditions (e.g., simply refluxing ZnO with TMP or TEP in water). Under these conditions, one of the P–OR bonds in phosphoric acid triesters underwent hydrolysis yielding the respective dialkyl phosphates (strong acids), which subsequently reacted with ZnO resulting in the formation of the large amounts of ZnDOPs (58% for ZnDEP and 96% for ZnDMP) (see Scheme 1). This hydrolytic strategy gave satisfactory results only for water-miscible triesters of H_3_PO_4_, albeit the yields of ZnDOPs quickly decreased when the size of organic group within the (RO)_3_PO molecules increased (96% versus 58% in the case of ZnDMP and ZnDEP synthesis, respectively).

Hydrolytic reaction utilizing highly hydrophobic TBP required harsher reaction conditions (e.g., temperature of 150 °C applied for 48 h) and replacing ZnO with water-soluble ZnOAc·2H_2_O to produce ZnDBP. Nevertheless, the latter was obtained with a yield of ca. 34%; however, its purification from the unreacted TBP and *n*-butanol (liberated as a side product of hydrolysis) was cumbersome. An attempt to obtain ZnDnPP directly from TnPP using a hydrolytic method did not lead to the formation of the intended product. Thus, we had to first synthesize DnPP (via a controlled dealkylation of TnPP with CaCl_2_, and subsequent liberation of DnPP from its calcium salt under the action of H_2_SO_4_), and then react it with ZnO.

In the second method, ZnDOPs having long alkyl chains in their phosphate ligands, were obtained with good yields directly from sodium salt of the commercially available phosphoric acid diesters (DBP or BEHP) and zinc acetate. The chemistry behind this method is very simple and comprises two consecutive stages carried out in water at room temperature: neutralization of acidic dialkyl phosphate with sodium bicarbonate, followed by the ion-exchange reaction between the resulting water-soluble sodium diorganophosphate and zinc acetate (see Scheme 1). Immediately after mixing the aqueous solutions of both substrates, the product precipitates and forms a fine dispersion of flocculent particles. From this dispersion ZnBEHP can be easily isolated almost quantitatively reaction yield of 95%). In comparison, the sedimentation of ZnDBP at room temperature is less efficient and the yield of isolated solid product typically does not exceed 30%; however, this can be easily changed by application of heating, since at temperatures around 55–60 °C the stability of ZnDBP dispersion is disrupted, resulting in a coalescence of the flocculated, small particles into a few, much larger agglomerates and an increase in the overall yield of ZnDBP up to 63%. It should be noted that the method described above is much more simple and environmentally benign than its other alternatives described in the literature for zinc-containing hybrid polymers, for example, the process based on a cation-exchange resin [38]. Because of the properties of ZnDOPs, especially their solubility in different solvents (see a discussion in one of the following subsections of our manuscript), the latter method would require the use of a large amounts of polar, organic solvents, such as methanol or DMSO.

The carbon, hydrogen and zinc contents in ZnDOPs prepared by the hydrolytic or ion-exchange routes are in very good agreement with the values calculated for the polymers free of any auxiliary ligands (see Table S1 in SM), thus clearly indicating that neither water nor any unreacted substrates are coordinated to the metallic centers. These results also confirm that the compounds investigated in the present study exhibit the same composition (e.g., the molar ratio of diorganophosphate ligands to zinc centers) and general formula {Zn[O_2_P(OR)_2_]_2_}, as ZnDOPs already mentioned in the literature, containing either aliphatic [4,16,18] or aromatic [21,33] substituents. Moreover, in the case of ZnDOPs obtained from triesters of H_3_PO_4_ via a hydrolytic route, they also exclude the possibility of an uncontrolled hydrolysis of phosphoester linkages leading to the formation of zinc salts containing either (ROPO_3_)^2−^ or PO_4_^3−^ ligands. A very similar conclusion can be made based on the analysis of the FTIR spectra of ZnDOPs (Figure S1 in SM), which do not contain any absorption bands above 3000 cm^−1^ or around 1650 cm^−1^ resulting from the stretching or bending vibrations in the zinc-coordinated H_2_O molecules, respectively [33]. Instead, one can easily observe the bands ascribed to the C–H stretching (the 2800–3000 cm^−1^ region), as well as bending and wagging modes (the 1360–1480 cm^−1^ region) within the methyl, methylene or methanetriyl groups [39,40]. The four vibrational bands characteristic of the phosphate ligand occur in the wavenumber region of 720–1200 cm^−1^: between 800–830 cm^−1^ and 730–770 cm^−1^ appear signals attributable to the P–O asymmetric and symmetric stretching modes within the phosphoester linkages, respectively, whereas their analogues from the Zn–O–P–O–Zn bridges are located around 1180–1195 cm^−1^ and 1095–1100 cm^−1^, respectively [39,40]. It is worth noting that the wavenumbers of the latter two vibrational bands in ZnDOPs are about 20–30 cm^−1^ lower compared to their analogues characterizing AlDOPs [15]. In addition to the absorption bands mentioned above, FTIR spectra of the aliphatic ZnDOPs also contain very strong signals ascribed to the C–O stretching modes located between 1000 and 1065 cm^−1^ [39,40].

The as-synthesized samples of ZnDMP and ZnDEP consist of highly crystalline, large particles exhibiting a cuboid or fiber morphology, respectively (Figure S7 in SM). Their PXRD patterns correspond very well to those simulated based on the diffraction data from single-crystal X-ray analyses carried out at room temperature and published by Harrison and coworkers [16,18] (Figures S8 and S9 in SM). No evidence of any other crystalline phases can be found in these samples, indicating that our hydrolytic method of synthesis can be an interesting alternative to the hydrothermal one proposed in the literature. It should be noted, however, that some ZnDMP samples recrystallized from their aqueous solution during a slow evaporation at 70 °C also contain some additional reflections on their PXRD patterns, indicating the presence of another crystalline phase with an unknown structure. This observation prompted us to closely investigate the relationship between temperature and ZnDMP structure, the results of which will be discussed in a separate section of this manuscript.

ZnDnPP also exhibits a highly ordered crystalline structure (Figure S10 in SM); however, the analysis of PXRD patterns obtained for ZnDBP and ZnBEHP (Figures S11 and S12 in SM, respectively) points out that a further elongation of alkyl chains (above C3) within diorganophosphate ligand hinders the mutual ordering of ZnDOP particles into large domains having the regular internal structure: a low crystallinity of both these materials is clearly indicated by a substantial widening of their PXRD reflections, especially in the case of ZnBEHP, the X-ray diffractogram of which also shows the presence of a broad halo located at 2*θ* angle of ca. 20°, which is characteristic of an amorphous phase (Figure S12 in SM). This conclusion is further supported by SEM, since only a small fraction of fiber- or rod-like structures is observed at a high magnification for the ZnDBP sample obtained at room temperature (Figure S13 in SM), whereas one cannot distinguish any discrete particles in the case of ZnDBP conditioned at 60 °C. The latter also applies to ZnBEHP and its samples exhibit a continuous morphology, without any distinct particles being visible (Figure S14 in SM).

**Figure 2:**
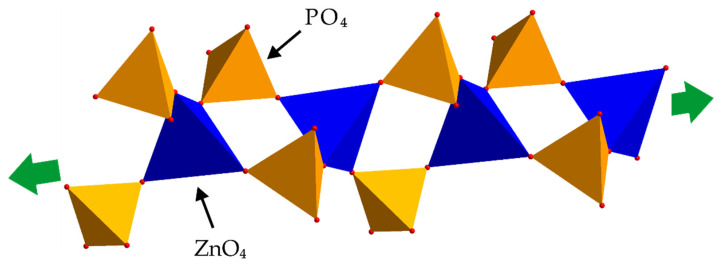
Model of a single polymeric chain proposed on the basis of the partially refined crystal structure of ZnDBP. Green arrows indicate the direction in which the ZnDBP chain propagates. For the clarity of presentation, carbon and hydrogen atoms are omitted, whereas red dots represent oxygen atoms.

For the purpose of structural studies, we have prepared the needle-like crystals of ZnDBP by means of its recrystallization from a mixture of DMSO and methanol (1:5 vol/vol) carried out at −20 °C. This material was subjected to a single-crystal X-ray analysis (details of that procedure are included in SM). Unfortunately, the high flexibility of aliphatic chains within the (*n*-BuO)_2_PO_2_ ligands resulting from rotations around the C–C bonds caused severe structural disorder outside the coordination polymer core. Although it did not permit full refinement and solution of that crystal structure, the connectivity pattern showing 2 + 2 bridging mode in the polymer chain is noted. Nevertheless, very good compliance between the theoretical PXRD pattern calculated based on this partially solved ZnDBP structure and the one recorded experimentally (see Figure S11 in SM) indicates that the former may be considered a good approximation of the actual ZnDBP crystal structure, especially concerning its rigid core formed by ZnO_4_ and PO_4_ tetrahedra sharing their vertices. According to our proposal, the basic structural unit of ZnDBP is a polymeric chain in which tetrahedrally coordinated Zn centers are lined up on a single axis, and two bidentate diorganophosphate bridging ligands connect each pair of them. As a result, in a single ZnDBP chain, there are eight-membered Zn(O–P–O)_2_Zn rings, the planes of which are arranged at an angle of ca. 90 (a spiro-type system) (Figure 2). The average Zn···Zn distance in that structure (4.578–4.589 Å) is within the range observed for other aliphatic ZnDOPs exhibiting the same 2 + 2 metal center bridging mode (4.546 Å for ZnDEP [16] or 4.665–4.697 Å for ZnDMP [18]), but longer than in its aromatic analogues (4.329–4.425 Å [21]). The PO_4_ groups show distortions from the structure of a regular tetrahedron since the P–O bond lengths within the Zn(O–P–O)_2_Zn rings are shorter than those responsible for bonding organic substituents, whereas the opposite effect occurs in the case of the angles between them. The O–P–O bond angles in the bridges between zinc centers (117.06–118.25°) are the largest ones and exceed the rest of them by about 5–20°. It should be noted that such features of the PO_4_ tetrahedra seem to be typical for ZnDOPs regardless of the type of organic groups in their structure.

### 3.2. Thermogravimetric Analysis and Thermal Stability of Aliphatic ZnDOPs

The results of STA analysis show that when heated in an inert atmosphere, the samples of aliphatic ZnDOPs start to endothermally decompose at temperatures between 215 and 300 °C (as suggested by the values of the bend points on the respective TG curves, *T*_b_, Table 1) and stop changing their weight at ca. 260–430 °C, depending on the type of organic substituents present within the (RO)_2_PO_2_ ligand (see the end points of the respective dTG peaks in Figures S15–S19 in SM). As could be expected, the chemical bonds in most of the investigated aliphatic ZnDOPs are more prone to thermolysis than those present within aromatic ZnDOPs, although some thermally labile functional groups attached to the phenyl ring may alter that hierarchy [21,33]. Taking into account the values of *T*_b_, temperature corresponding to 2% or 5% of weight loss (*T*_98%_ or *T*_95%_, respectively), as well as the extrapolated onset temperature of thermolysis (*T*_onset_), it is evident that ZnDOPs containing short (C1–C3) aliphatic chains are also less stable than their aluminum-based analogues having the same diorganophosphate ligands (AlDOPs) [35]; however, this situation changes in the case of ZnDBP or ZnBEHP, whose pyrolysis is characterized by a higher energy barrier (initial temperature). Surprisingly, the latter compound is the most resistant to temperature among all of the investigated ZnDOPs, even when compared to ZnDMP—a derivative that can decompose only via more complex and energy-consuming processes involving homolytic cleavage of the P–OCH_3_ or PO–CH_3_ linkages and subsequent transformations of the liberated radicals [35,41]. The reason for such an unusually high thermal stability of ZnBEHP is still unclear and needs a more detailed, experimental investigation, which was out of the scope of the present study.

QMS analysis of the evolved gases (Figures S15b–S19b in SM) reveals that the chemical reactions occurring during pyrolysis of aliphatic ZnDOPs are limited only to the P–O–C bond system, whereas Zn–O–P linkages are resistant to any scission: no QMS peaks attributed to the typical volatile oxophosphorus species (such as PO, PO_2_, HOPO, or HOPO_2_; *m*/*z* = 47, 63, 64, or 80, respectively) [42] could be detected. Moreover, a complete lack of any analytical signals above *m*/*z* = 60 (70 in the case of ZnBEHP) is strong evidence that, similar to the pyrolysis of AlDOPs [35], the thermal degradation of aliphatic ZnDOPs in inert atmosphere proceeds essentially via the C–O bond splitting reactions involving the β-elimination of olefines (e.g., ethylene, *m*/*z* = 28, propene, *m*/*z* = 41 and 42, 1-butene, *m*/*z* = 41 and 56) [43], as well as a subsequent rearrangement between the newly generated POH and the remaining phosphoester groups, leading to the OH-containing species [44] (e.g., primary alcohols giving characteristic QMS signal of ^+^CH_2_OH ions, *m*/*z* = 31 [45]). The product that indirectly indicates the occurrence of the P–OR bond scission (e.g., ether resulting from the recombination of alkyl and alkoxy radicals [41]) can be observed only in the case of ZnDMP pyrolysis—signals derived from dimethyl ether (*m*/*z* = 45 and 46) [43], or formaldehyde (*m*/*z* = 29 and 30) [43] are easily distinguishable on the QMS spectrum. It is worth noting that a common feature of the aliphatic ZnDOPs pyrolysis is a simultaneous condensation of the POH groups leading to the elimination of water molecules (*m*/*z* = 17 and 18) [43].

### 3.3. Thermal Transitions in Aliphatic ZnDOPs below 200 °C

The DSC studies reveal that ZnDOPs undergo several endothermic phase transitions on heating from −100 to 200 °C. These processes proceed without any weight loss; additionally, based on the optical observations conducted on a Gallenkamp Melting Point Apparatus, they can be categorized into two groups: *solid*→*liquid* transitions and those occurring without any change in the physical state of the sample (*solid*→*solid* transitions). Some of them are reversible and are accompanied by the exothermic peaks on the DSC cooling curves, albeit the latter usually occurs at temperatures several degrees lower than the respective endotherms.

**Figure 3:**
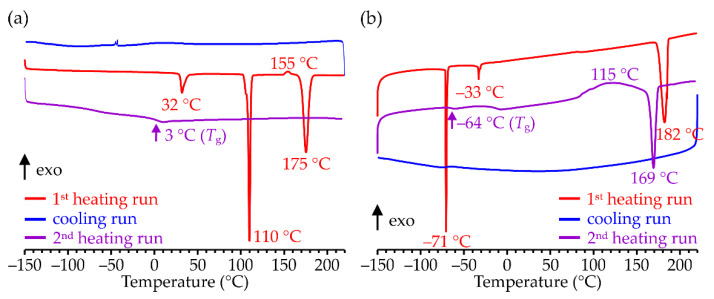
DSC traces of the pristine samples of: (**a**) ZnDMP, and (**b**) ZnDEP. The measurements were carried out in the following order of runs: 1st heating (red lines), cooling (blue lines, 2nd heating (purple lines). The glass transition temperatures are marked with arrows.

On heating, the pristine ZnDMP sample exhibits three endothermic transitions with maxima located at 32, 110 and 175 °C (Figure 3a), whose enthalpies are equal to −2.4, −12.4 and −12.9 kJ/mol, respectively. The first two of them proceed in a solid state, while the one with the highest peak temperature arises simply from the melting of the sample. Interestingly, there exists also a small exothermic peak (Δ*H* = 0.6 kJ/mol)) situated prior to the melting endotherm (its maximum at ca. 155 °C)—a very similar situation occurs in the case of several semi-crystalline polymers (e.g., polylactide) [46] undergoing some structural rearrangement (for example, a disorder-to-order crystal phase transition) immediately before melting.

The systematic analysis of the X-ray diffractograms recorded at different temperatures by means of the VT–PXRD method gives evidence that the crystal structure of ZnDMP does not change significantly within the temperature range of 25–105 °C. As could be expected, within that area the increase in temperature resulted only in a gradual expansion of the ZnDMP unit cell volume due to its polymeric chains moving away from each other in a direction perpendicular to their axes. The results of our calculations (see Table S2 in SM) show that within the margin of estimation error this process is anisotropic since it increases the value of the *c*-axis unit length of ZnDMP, whereas the remaining lattice parameters *a* and *b* decrease their values (albeit with some fluctuations). It should be noted that the almost linear and continuous character of those changes is indicative of the absence of any polymorphic transitions during the heating of this low-temperature crystalline phase of ZnDMP (denoted as α-ZnDMP), which is structurally identical with the one determined by Harrison and coworkers for ZnDMP monocrystal [18].

On heating above 105 °C (i.e., in the temperature region that corresponds to the first strong endothermic transition observed in the 1st heating DSC curve of the pristine ZnDMP), the PXRD reflections ascribed to α-ZnDMP start to disappear, while new ones show the indicating transformation of α-ZnDMP into a new crystalline phase (denoted as β-ZnDMP—during the VT-PXRD measurement, this process is completed at 165 °C (Figure S20 in SM)). We tried to carry out a full profile refinement of the latter’s cell parameters, but we did not get any reliable results due to an insufficient number of observed reflections. Rapid cooling of the ZnDMP sample previously conditioned for 3 h at 130 °C does not reverse the α-ZnDMP → β-ZnDMP transition since the PXRD pattern of such a sample recorded at room temperature (Figure S21a in SM) reveals a set of reflections attributable solely to β-ZnDMP—two of them exhibiting the highest intensity (2*θ* angle of 9.626° and 13.277°) can be considered diagnostic PXRD signals for an identification of this crystalline phase. It is worth noting that β-ZnDMP is stable at room temperature and does not undergo any structural rearrangement while being stored for several days—nevertheless, after 7 days at that temperature, a development of the PXRD reflection characteristic for α-ZnDMP (2*θ* = 11.263°) could be detected (Figure S21b in SM). This process can be accelerated even further if the β-ZnDMP sample is conditioned at the temperature near that of the α-ZnDMP → β-ZnDMP transition (see Figure S21a in SM).

The PXRD reflections of β-ZnDMP are clearly visible up to about 165 °C, but on further heating they gradually disappear, and above 180 °C, the sample of ZnDMP becomes an isotropic, viscous liquid (Figure S20 in SM). Upon rapid cooling during a DSC test, it transforms into an amorphous solid that shows a second-order transition around 3 °C (see the 2nd heating DSC curve in Figure S3a).

The DSC heating curve of pristine ZnDEP (Figure S3b) contains 3 endotherms with maxima at approximately −71 °C (Δ*H* = −7.5 kJ/mol), −33 °C (Δ*H* = −0.8 kJ/mol) and 182 °C (Δ*H* = −21 kJ/mol). Similar to ZnDMP, when heated above 180 °C ZnDEP becomes a viscous liquid, the cooling of which leads to the formation of an amorphous material exhibiting a second-order transition at −64 °C and no DSC peaks below room temperature. In contrast to ZnDMP, the amorphized ZnDEP sample is prone to a slow cold crystallization during the 2nd heating run—a process that begins above 80 °C, ends around 150 °C and turns immediately into melting. Interestingly, its enthalpy closely matches that of the melting, and they both constitute approximately 80% of the value of enthalpy of melting detected during the 1st heating cycle. Moreover, the crystalline domains formed during cold crystallization are characterized by a lower melting temperature (169 °C) than the pristine ZnDEP.

In order to investigate the first two endothermic processes occurring during the heating of ZnDEP, we carried out the DSC and VT-PXRD measurements between −100 and room temperature. The obtained calorimetric results (Figure S22 in SM) indicate that both those transitions are fully reversible since their endotherms are accompanied on the DSC cooling curves by the respective exothermic peaks characterized by almost the same absolute values of enthalpy: between cooling and heating mode, the maxima of the related DSC peaks are shifted by only ca. 5−8 °C relative to each other. Previously, a very similar situation was observed on the DSC trace of the aluminum-based analogue of ZnDEP, aluminum tris(diethylphosphate), and the overcoming of energy barriers related to the rotations around C–C and C–O bonds within the (RO)_2_PO_2_ ligand was proposed as the source of that low-temperature DSC endotherms [35]—the same explanation can be given in the case of ZnDEP. In the latter’s crystal structure determined by Harrison and coworkers at room temperature [16], all ethyl groups around phosphorus center occur in the *gauche*-*anticlinal* (G^−^A^−^) conformation (the O–P–O–C torsion angles of −75(1)° and −104(2)°), which has a higher potential energy than the most stable *gauche*-*gauche* conformer (or the *trans*-*gauche*-*gauche*-*trans* one, if both the carbon-oxygen and phosphorus-oxygen torsion angles are considered) [39,40]. One can expect that with a decreasing temperature this structure might be inclined to adopt a spatial configuration with less potential energy, thus giving rise to exotherms observed on the DSC cooling curve. The results of the VT-PXRD measurements (Figure S23 in SM) support this hypothesis, since the temperature-dependent structural changes below 0 °C impact mainly the reflections located at the high 2*θ* angle region of the ZnDEP X-ray diffractogram, which originate from diffractions occurring on the planes at least partially created by carbon atoms—for example, the (401) plane (see Figure S24 in SM) disappears between −70 and −80 °C. The two main reflections ascribed to the (200) and (110) planes, which are parallel to the axis of the ZnDEP polymeric chain and pass through the positions of zinc atoms, undergo only the typical changes resulting from a temperature-driven variation of the unit cell dimensions (e.g., they shift to higher 2*θ* values with lowering temperature due to a contraction of the ZnDEP unit cell and its polymeric chains getting closer to each other). Based on this conception, the lack of any structural transitions in ZnDMP below 100 °C is entirely understandable, since at room temperature the methyl groups in α-ZnDMP are already in the most stable *gauche*-*gauche* arrangement, according to the crystallographic data published in the literature [18]. Still, the presented explanation is only a hypothesis that needs further, experimental verification (e.g., ZnDEP single crystal XRD measurements below 0 °C), since other structural factors related to the thermal shrinkage of the ZnDEP unit cell may also play an important role in those processes.

Thermal effects of the subzero temperature structural transitions are also observed for several other homologues of ZnDEP containing longer alkyl chains, such as: *n*-propyl (between ca. −14 and −27 °C), *n*-butyl (between −88 and −95 °C) and 2-ethylhexyl (between −25 and −42 °C) (see Figure S25 in SM). Based on the VT-PXRD measurements of the cooled samples of ZnDnPP (Figure S26 in SM) or ZnBEHP (Figure S27 in SM), one can conclude that with the lowering of temperature the crystal structures of these compounds not only undergo a standard thermal contraction resulting in a shift of PXRD reflections to higher values of 2*θ* angle, but also some larger reorganization of their atoms giving rise to new X-ray diffraction planes. In both these derivatives, the low-temperature processes are related mainly to the X-ray reflections with the highest intensities and the lowest 2*θ* values, which most probably have the same origin as in ZnDEP (e.g., zinc atoms in the parallelly located ZnDOP polymeric chains). This means that the proposed structural reorganization must involve not only the conformational changes within organic substituents, but also a non-continuous relocation of the Zn(OPO)_2_Zn rigid cores. However, due to the lack of structural data, the exact nature of the abovementioned changes remains unknown and needs further investigation. Interestingly, a careful analysis of the DSC traces (see SM, Figure S25a for ZnDnPP and Figure S25c for ZnBEHP) and VT-PXRD patterns recorded during cooling and heating steps indicates that the occurring structural transformations seem to be completely reversible, since the original crystal structure of the previously cooled samples of ZnDnPP or ZnBEHP is restored upon their reheating to room temperature. This restoring is slower than the opposite process, since the characteristic reflections of the low-temperature phases are still observed on the PXRD diffractograms at the temperatures higher by 10–20 °C than those during the earlier cooling step—for example, α- and β-ZnBEHP phases coexist at room temperature within the sample at the end of the cooling-heating procedure, while only the former was present at the beginning of it (see Figure S27 in SM). It should be emphasized that the temperature window in which all these low-temperature structural transformations proceed corresponds very well to the one defined by the positions of the endo- and exotherms observed on the DSC heating and cooling curves below room temperature. It is also worth noting that, apart from the transition detected at the subzero temperature region, ZnDOPs with long alkyl (C3–C8) substituents also undergo another low-temperature structural reorganization around room temperature (ZnBEHP), or close to it (ZnDnPP and ZnDBP), the nature of which strongly depends on the type of organic group: ZnDnPP becomes a very viscous liquid, while the samples of the *n*-butyl and 2-ethylhexyl derivatives remain crystalline solids (although with a changed structure). Upon cooling from a temperature higher than that of the second structural transition, the melted *n*-propyl derivative solidifies into a material which do not show any transformations at the subzero temperature region, and whose melting temperature (55.8 °C) and enthalpy of fusion (Δ*H* = −6.6 kJ/mol) differ from those exhibited by the pristine ZnDnPP sample (62.1 °C and Δ*H* = −18.2 kJ/mol, Figure S25a in SM). At room temperature, ZnDBP preheated earlier to 90 °C exhibits the PXRD pattern, which differs from that of the initial sample (see Figure S11 in SM) indicative of its temperature-impeded structural reorganization, and similarly to an analogous sample of ZnDnPP is structurally stable at the subzero temperatures (no thermal effects below room temperature during the DSC 2nd cooling run, see Figure S25b in SM).

### 3.4. Aliphatic ZnDOPs in Solution

**Figure 4:**
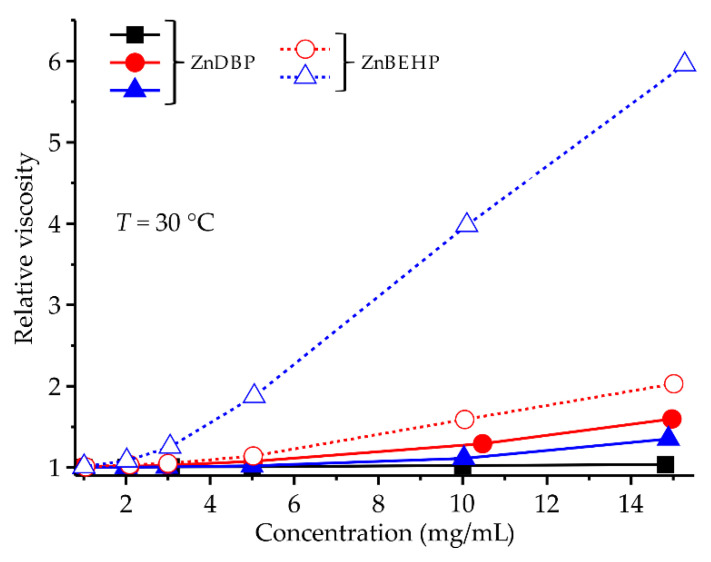
Variation of relative viscosity with concentration of ZnDBP or ZnBEHP solutions in selected organic solvents: methanol (black line), chloroform (red line) and toluene (blue line). The measurements were carried out at 30 °C.

The solubility of aliphatic ZnDOPs depends significantly on the type of the alkyl substituent in their molecule. The butyl and 2-ethylhexyl derivatives are soluble in chloroform and aprotic, nonpolar solvents such as cyclohexane, benzene, or toluene (ZnDBP has also good solubility in methanol), and we have investigated the variation in the relative viscosity of the resulting solutions with their concentration (1–15 mg/mL) at 30 °C—the results of these studies are shown in Figure 4.

In diluted solutions (sample concentration up to 3 mg/mL), only a slight linear increase in the relative viscosity values is observed, which can be attributed mainly to the interactions between solvent and the individual molecules of ZnDOPs. On the other hand, at higher sample concentrations the rheological behavior of the system depends on the type of the applied solvent: in methanol, the viscosity of solutions is almost constant, while in the case of systems based on less polar chloroform or toluene it increases significantly. One can assume that in the last two solvents ZnDBP and ZnBEHP exist in the form of large (polymeric) particles and above a certain critical concentration, hydrodynamic interactions between them give rise to the additional viscosity increase.

**Figure 5:**
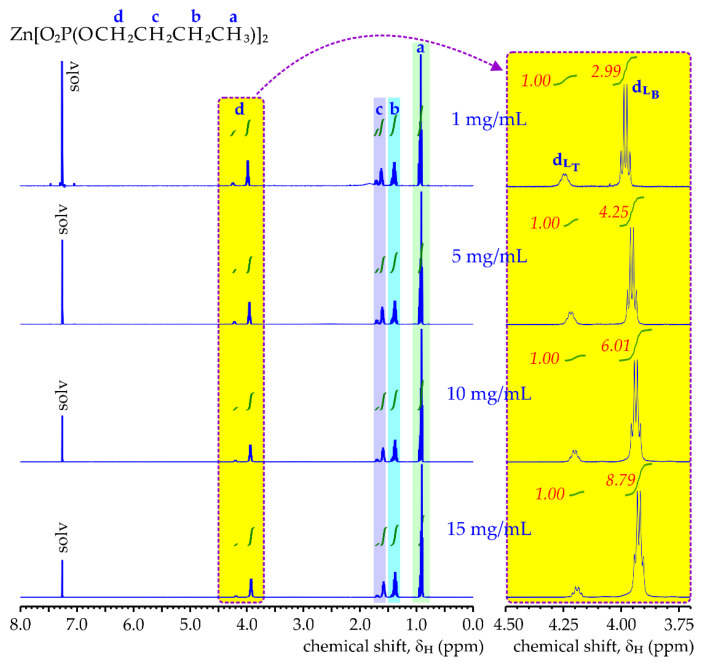
^1^H NMR spectra recorded for different mass concentrations of ZnDBP (1–15 mg/mL) in CDCl_3_ at: 0–8 ppm region (left side) and 3.7–4.5 ppm region (right side). Selected signal integrals are presented as green curves together with their values (red fonts). Abbreviations: solv—solvent residual peak, d_LT_—signal of POC*H*_2_C_3_H_7_ group within the terminal (RO)_2_PO_2_ ligand, d_LB_—signal of POC*H*_2_C_3_H_7_ group within the bridging (RO)_2_PO_2_ ligand. The ^1^H NMR signals were assigned to the different groups of protons appearing within the ZnDBP formula using small letters a–d.

Figure 5 shows the ^1^H NMR spectra of ZnDBP dissolved in CDCl_3_. These spectra contain signals characteristic for protons within the *n*-butyl chain constituting: methyl group (*δ*_H,a_ ≈ 0.9 ppm,), two inner methylene groups (*δ*_H,b_ ≈ 1.4 ppm*δ*_H,c_ ≈ 1.6–1.7 ppm) and methylene moiety connected to oxygen (*δ*_H,d_ at 4.0–4.2 ppm). As can be seen, a common feature of these spectra is the fact that each peak of the chemically equivalent group of protons is divided into two components having very similar multiplicity and chemical shifts (the difference in their *δ*_H_ values not larger than 0.3 ppm), but significantly differing in integrals—it is especially evident in the case of the nuclear resonance of the most deshielded protons from the –OCH_2_– group. Interestingly, the relative peak integral of the main component increases when increasing the concentration of the ZnDBP solution. A plausible explanation for that phenomenon is that within each NMR signal the main multiplet comes from diorganophosphate ligands in the polymer bridging groups (L_B_), whereas the second one with a smaller integral can be associated with (RO)_2_PO_2_ groups at the ends of the ZnDBP polymeric chain (terminal ligands, L_T_)—exactly the same “duplication” of ^1^H NMR signals is reported in the literature for ZnDMP [18] and ZnDEP [16] solutions in D_2_O.

Taking into account the zero electric charge of the ZnDBP chain, individual charges of its components {Zn^2+^ and [(RO)_2_PO_2_]^−1^ ions} and the fact of its formation via Zn(OPO)_2_Zn bridging units, one can expect that every ZnDBP (or generally ZnDOP) chain should have one diorganophosphate group at each of its ends, meaning two L_T_ ligands per single polymeric chain. Based on this, the formula of ZnDBP can be rewritten as L_T_[Zn(L_B_)_2_]_(*n* − 1)_ZnL_T_. The L_B_/L_T_ NMR peak integral ratio allows one to calculate the average number of repeating units (degree of polymerization, *n*) in a ZnDBP molecule (L_B_/L_T_ = *n* − 1), and its value depends on the concentration of the sample. As can be seen from data presented in Table 2, for the investigated range of the concentration of ZnDBP solution in CDCl_3_, the *n* value changes from ca. 4 (ZnDBP tetramer) up to 10 (ZnDBP dekamer), which corresponds to the number average molecular weight between 1900 and 4800 g/mol, respectively.

Conclusions very similar to the ones described above can be made based on the analysis of the ^31^P NMR spectra of ZnDBP solutions in CDCl_3_ (Figure S28 in SM). They contain two peaks located around −2 ppm (main signal) and −13 ppm—a relative integral ratio of the former to the latter increases when increasing the mass concentration of the respective solution. The estimated degrees of polymerization and number average molecular weights are very close to the values derived from ^1^H NMR—the observed small differences can result from lower signal-to-noise ratio and problems with peak integration occurring in the case of phosphorus NMR.

It should be noted that ^1^H and ^31^P NMR spectra of ZnDBP in either non-polar (benzene-d_6_, Figure S29 in SM), or strongly polar (DMSO-d_6_, methanol-d_4_, Figures S5 and S30 in SM, respectively) solvents show no differentiation between the inner and terminal diorganophosphate ligands: only one set of the proton resonance peaks ascribed to the *n*-C_4_H_9_ group and one population of phosphorus nuclei are visible, respectively, suggesting that in these solvents ZnDBP molecules are either in the form of long polymeric chains (in which the concentration of the chain-ends is below the detection point of the NMR method), or they are completely depolymerized to monomeric species or free ions. The same applies to other ZnDOPs, which, in the ^31^P NMR spectra recorded in a polar solvent, give one average signal at about 0–−2 ppm (see Figures S1–S4 in SM). In order to further investigate the abovementioned phenomena, we have measured the diffusion coefficients of ZnDBP particles (*D*_ZnDBP_) in diluted solutions (mass concentration of 1 mg/mL) in perdeuterated methanol, chloroform and benzene at 25 °C by means of the ^1^H DOSY NMR method. The obtained values of *D*_ZnDBP_ are summarized in Table 3, together with those of the ZnDBP hydrodynamic radii (*R*_H,ZnDBP_) estimated based on the Stokes–Einstein equation [46,47]:

(1) $$D_{\mathrm{ZnDBP}}=\frac{k_{B}T}{6\pi \eta R_{H,\mathrm{ZnDBP}}f},$$

where *k*_B_ is the Boltzmann constant, *T* is the absolute temperature, *f* is friction factor (*f* = 1 for a hard sphere), and *η* is the solution viscosity.

**Scheme 2:**
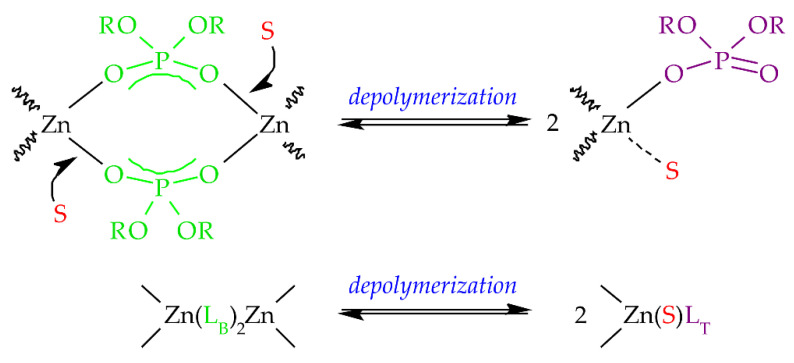
Interaction of ZnDOP polymeric chains with polar solvent (S), and formation of oligomeric species.

Although the presented data should be considered a gross approximation of the real values of *R*_H,ZnDBP_ (for example, they do not take into account the deviation of the shape of ZnDBP particles from sphericity), one can conclude that the size of ZnDBP particles strongly depends on the polarity of the solvent. The largest particles are found in nonpolar C_6_D_6_, while the smallest ones are found in CD_3_OD solution. Probably polar solvents can complex zinc atoms in ZnDBP (or ZnDOPs in general) and break down the bridging bonds into small oligomers, as shown in Scheme 2, or mononuclear complexes.

### 3.5. Synthesis and Characterization of Hybrid Copolymers

**Scheme 3:**
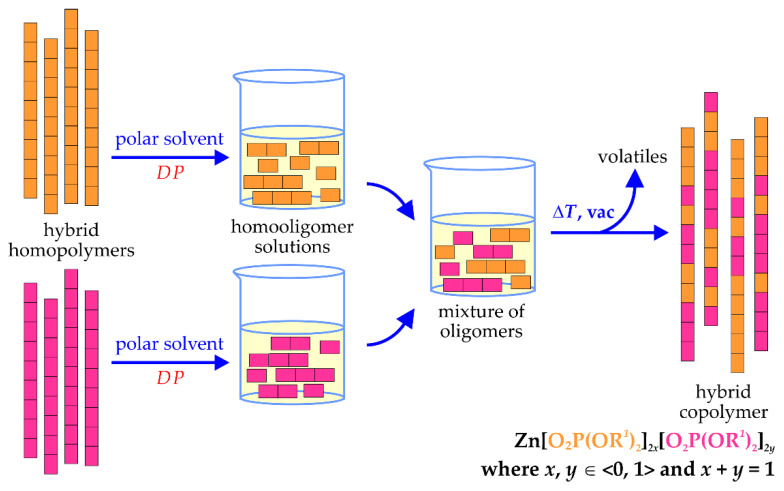
Synthesis of a hybrid copolymer through randomization of monomeric units of two different ZnDOPs depolymerized in polar solvent. Abbreviations: *DP*—depolymerization; vac—vacuum distillation.

To confirm the reversible dissociation of aliphatic ZnDOPs in polar solvents, we have made attempts to obtain hybrid copolymers by dissolving two homopolymers in methanol and then evaporating the volatiles (see Scheme 3). For that purpose, we have used both the aliphatic ZnDOPs described in this manuscript, as well as their simplest aromatic analogue, namely, ZnDPhP, which was fully characterized in our previous work [33].

We have found that the abovementioned procedure leads to fully amorphous or semicrystalline materials, which are well-soluble in non-polar solvents. Figures S31–S33 (SM) contain ^1^H DOSY NMR spectra recorded at 25 °C for several hybrid copolymers dissolved in C_6_D_6_ (a mass concentration of each system was 5 mg/mL), whereas Table 4 shows the values of their diffusion coefficients (*D*_cop_) and hydrodynamic radii (*R*_cop_) approximated based on the Stokes–Einstein formula (see Equation (1), where *D*_ZnDBP_ and *R*_H,ZnDBP_ are replaced with *D*_cop_ and *R*_H,cop_, respectively).

It should be noted that the *D*_cop_ values for the various groups of protons in the respective hybrid copolymer are the same within the experimental error. Thus, it can be assumed that the different organic substituents (e.g., *n*-butyl and phenyl groups in the case of ZnDBP-*co*-ZnDPhP copolymer) present in its structure constitute the same macromolecule. The mobility and *R*_H,cop_ of the copolymer molecules depends on the type of diorganophosphate ligands. For example, a copolymer containing methyl and ethyl groups (ZnDMP-*co*-ZnDEP) forms smaller molecules with a higher diffusion coefficient than its analogue bearing longer alkyl chains—*n*-butyl and 2-ethylhexyl groups (ZnDBP-*co*-ZnBEHP).

More detailed studies carried out for the copolymer containing phenyl and *n*-butyl groups (ZnDBP-*co*-ZnDPhP) show that the distribution of ligands in its chains is not fully uniform and, by using an appropriate solvent, it can be separated into fractions with different compositions. For example, ^1^H NMR analysis (Figure S34 in SM) indicates that a sample of ZnDBP-*co*-ZnDPhP, in which the overall molar ratio of the aromatic and aliphatic monomeric units is 1:1, contains a fraction of polymeric chains enriched with aromatic ligands (the molar ratio of (PhO)_2_PO_2_/(*^n^*BuO)_2_PO_2_ is 4:1) and is insoluble in cyclohexane—just like pure ZnDPhP homopolymer. At the same time, the cyclohexane-soluble part of that sample retains the overall equimolar composition. The ^31^P NMR spectrum of this copolymer in C_6_D_6_ (Figure S35 in SM) contains two signals located at −1.18 and −12.94 ppm, which can be attributed to phosphorus nuclei in the bridging diorganophosphate ligands containing aliphatic and aromatic groups, respectively. Taking into account that ZnDPhP homopolymer is insoluble in non-polar media, the presence of its ^1^H and ^31^P NMR peaks in the spectrum of ZnDBP-*co*-ZnDPhP suggests that zinc diphenylphosphate monomeric units or blocks “gain” solubility in C_6_D_6_ due to their incorporation into the macromolecule containing blocks of ZnDBP.

**Figure 6:**
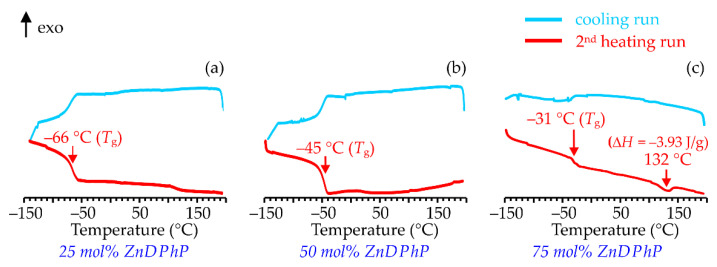
DSC traces of the ZnDBP-*co*-ZnDPhP copolymers containing different amounts of aromatic monomeric units: (**a**) 25 mol%, (**b**) 50 mol%, and (**c**) 75 mol%. The cooling and 2nd heating runs are presented.

The DSC thermograms of the ZnDBP-*co*-ZnDPhP copolymers indicate the presence of a homogeneous amorphous phase, whose glass transition temperature (*T*_g_) depends on the molar ratio of the alkyl and aromatic (RO)_2_PO_2_ ligands—for copolymers containing 25, 50 and 75 mol% of diphenylphosphate units the *T*_g_ values are −66, −45 and −31 °C, respectively (Figure 6). Interestingly, in the ZnDBP-*co*-ZnDPhP copolymers with an excess of monomeric units of a given type, a small fraction of the crystalline domains may also occur—they give rise to the PXRD reflections (e.g., the one with the highest intensity located at 2*θ* = 7.87°, Figure S36 in SM) closely matching those previously reported for the samples of a pure ZnDPhP [33]. It is possible that in these systems a high concentration of oligomers of one type favors a quick formation of larger particles of the respective homopolymer, thus changing the overall molar ratio of the remaining alkyl and aryl building blocks from which a copolymer molecule can be synthesized.

### 3.6. Curing of Epoxy Resins in the Presence of ZnDOPs

**Figure 7:**
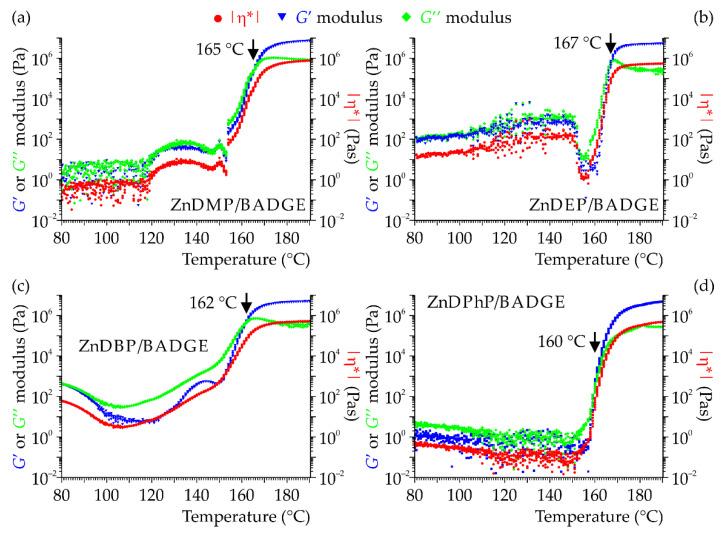
Variation of the complex viscosity (|η*|), storage modulus (*G*′) and loss modulus (*G*″) with temperature during thermal curing of the ZnDOP/BADGE mixtures containing 20 wt%: (**a**) ZnDMP (ZnDMP/BADGE), (**b**) ZnDEP (ZnDEP/BADGE), (**c**) ZnDBP (ZnDBP/BADGE), or (**d**) ZnDPhP (ZnDPhP/BADGE). The lowest temperature at which *G*′ ≥ *G*″ is indicated with an arrow.

The results presented above suggest that ZnDOPs are Lewis acids and can form complexes with organic compounds of nucleophilic properties. This feature prompted us to conduct some preliminary studies on the potential catalytic activity of ZnDOPs in the curing of epoxy resin—for that purpose, a thermosetting epoxy monomer, bisphenol A diglycidyl ether (BADGE), was chosen. Initially, we observed that by heating above 160 °C, one can solidify BADGE compositions containing a few wt% of ZnDMP, ZnDEP, ZnDBP or ZnDPhP (namely, ZnDOP/BADGE compositions), whereas its pure sample remains liquid under similar treatment. This finding led us to have a closer look into the changes of viscoelastic properties occurring during the heating of ZnDOP/BADGE by utilizing the method of oscillatory rheology. In these experiments, the changes in the complex viscosity (|η*|), storage modulus (*G*′) and loss modulus (*G*″) were recorded while heating the samples at a constant rate from 80 to 190 °C. The results from these measurements carried out for the compositions filled with 20 wt% of the selected ZnDOPs are presented in Figure 7.

As can be seen, upon heating to ca. 150 °C, the viscous properties prevail in all systems since *G*′ has lower values than *G*″: within this temperature region, the compositions behave like viscous liquids. Around 140–150 °C, a transition region starts, in which both moduli and viscosity rapidly increase their values. Subsequently, between 160 and 170 °C the storage modulus reaches values higher than *G*″, indicating that the material’s elastic properties start prevailing and it becomes solid. The whole process ends at around 180 °C and all of the studied rheological parameters stabilize their values at levels that are 5–7 orders of magnitude higher than those at the beginning of the curing process.

The changes in rheological parameters in the temperature range of 80–140 °C are much smaller and depend on the type of the curing agent. In the case of ZnDPhP/BADGE, the values of these parameters decrease slightly, whereas in compositions based on ZnDMP, ZnDEP or ZnDBP, some fluctuation in |η*|, *G*′ and *G*″ are observed, which may be related to changes in the structure of the respective ZnDOP and initiation of the curing process.

The thermal effects during the isothermal curing of BADGE in the presence of 20 wt% of ZnDEP were investigated within a 30 min time span at the temperatures of 130, 140, 150 and 160 °C, using the DSC method (Figure S37 in SM). The results of these tests show that at 130 °C a possible exothermic transition (polymerization of the epoxy groups) requires at least 26 min to begin. At higher temperatures, the induction period is getting shorter (starting from about 10 min at 140 °C to virtually immediate polymerization at 160 °C), and the maximum curing rate is reached after 3.5–23 min. Based on these results, we think that ZnDOPs can be used as very effective, latent curing agents in the so-called one-component epoxy resin composition. An important advantage is that such compositions are stable when stored at ambient conditions, and will cure quickly when heated to a moderately high temperature.

The curing process often leads to a change in the structure of ZnDOPs. For example, the PXRD measurements (Figure S38 in SM) clearly show that the filler/catalyst particles in the ZnDMP/BADGE composition (a 20 or 50 wt% filler load) hardened at 160 °C have an amorphous structure, while in the case of the same materials cured at lower temperature (130 °C) one can also observe the reflections attributable to β-ZnDMP, with only a small trace of those characteristic for the structure of ZnDMP before curing (its α phase). On the other hand, the crystalline domains of ZnDPhP immobilized in the epoxy resin are detectable at polyepoxide hardened at 160 °C (Figure S39 in SM), albeit only for the more concentrated system—their structure corresponds very well to the one exhibited at the same temperature by the high temperature ZnDPhP crystal phase forming at temperatures exceeding 145 °C [33]. Therefore, it can be suspected that in the solidified polyepoxide matrix the particles of ZnDOPs at least partially (their amorphization always occurs) retain the ordered structure they adopt at the temperature applied during curing. Moreover, it is likely that, beside the catalytic activity, some of these particles can also react with either epoxide or the newly formed polyepoxide; however, this hypothesis needs more detailed studies since the structure of the resulting products, as well as the mechanism of the epoxide curing, has not been fully elucidated yet.

## 4. Conclusions

We have developed very simple and efficient methods for the synthesis of hybrid polymers based on zinc bis(diorganophosphate)s, in which commercially available tri- or diesters of phosphoric acid and water-soluble zinc acetate are utilized. Solid samples of alkyl ZnDOPs show the ability to undergo a structural reorganization under mild thermal conditions. In the solid state, the compounds form linear chains in which zinc atoms are linked by two dialkylphosphate ligands. Homopolymers with the methyl or ethyl substituents form highly crystalline products with melting points in the range of 170–180 °C, whereas ZnDnPP seems to undergo the *solid*→*liquid* transition below 100 °C.

In polar solvents (e.g., methanol, DMSO, or water in the case of ZnDMP and ZnDEP), ZnDOPs undergo a reversible dissociation, which allows one to obtain statistical hybrid copolymers by reshuffling diorganophosphate anions. The properties of the resulting compounds strictly depend both on the types and molar ratio of their constituting monomeric units. ZnDOP homopolymers with long alkyl substituents and copolymers containing two different organic substituents are semi-crystalline or amorphous solids, which easily dissolve in non-polar organic solvents and form solutions whose viscosity increases strongly above a certain critical concentration.

Some preliminary observations indicate that ZnDOPs can be used as the environmentally friendly, latent curing agents for epoxy resins, which can be an interesting alternative to the conventional dicyandiamide-based systems. The examples of other potential applications of ZnDOPs, e.g., as flame retardants, nucleators and reinforcing fillers for organic polymers, will be presented in the forthcoming contributions.

## Figures and Tables

**Table 1:** Thermogravimetric data of aliphatic ZnDOPs measured in argon.

Compound	Type of Process ^1^	*T*_b_ ^2^ (°C)	*T*_98%_ ^3^ (°C)	*T*_95%_ ^3^ (°C)	*T*_onset_ ^4^ (°C)	*T*_max_ ^5^ (°C)	*T*_endset_ ^4^ (°C)	Δ*m* ^6^ (wt%)	*m*_800_ ^7^ (wt%)
ZnDMP	multistep	231.0	273.2	291.6	287.4	308.6	nd ^8^	31.16	67.99
					nd ^8^	340.9	nd ^8^		
					nd ^8^	345.2	352.2		
ZnDEP	one-step	225.0	264.0	280.1	299.9	306.9	309.1	44.62	53.83
ZnDnPP	one-step	257.0	284.3	293.9	297.8	301.3	302.9	51.24	47.59
ZnDBP	one-step	241.0	280.1	301.4	306.4	309.3	312.4	56.44	42.83
ZnBEHP	one-step	296.0	304.7	306.0	306.5	309.2	312.1	68.02	30.86

^1^ Refers to the number of steps in which thermal decomposition occurred (e.g., one–step—a single, well-defined step of weight loss, multistep—many steps of weight loss, some overlapping with each other). ^2^ Temperature of the beginning of weight loss (a bend point on the TG curve). ^3^ *T*_98%_ and *T*_95%_ denote temperatures at which sample mass reached 98% and 95% of its initial value, respectively. ^4^ *T*_onset_ and *T*_endset_ denote the extrapolated onset and endset temperatures of the first and last degradation step, respectively. ^5^ Temperature at the maximum decomposition rate. The value ascribed to the main weight loss step is underlined. ^6^ Total mass variation during all decomposition steps (as % of the initial sample weight). ^7^ Residual mass of the sample measured at 800 °C (as % of the initial sample weight). ^8^ not determined due to overlapping of the peaks.

**Table 2:** The degree of polymerization (*n*) and number average molecular weight (*M*_n_) of the oligomeric species existing in the ZnDBP solutions in CDCl_3_ calculated based on ^1^H and ^31^P NMR data.

Solution Concentration (mg/mL)	^1^H NMR ^1^		^31^P NMR ^2^	
	*n* ^3^	*M*_n_ (g/mol)	*n* ^3^	*M*_n_ (g/mol)
1	4.0	1940	nd ^4^	nd ^4^
5	5.3	2560	5.9	2850
10	7.0	3390	8.8	4260
15	9.8	4740	12.4	6000

^1^ Values calculated based on the data presented in Figure 5. ^2^ Values calculated based on the data presented in Figure S28 (SM). ^3^ Values calculated from the formula *n* = L_B_/L_T_ + 1. ^4^ Not determined due to insufficient value of the signal-to-noise ratio.

**Table 3:** The results of ^1^H DOSY NMR measurements carried out at 25 °C for the solutions of ZnDBP in perdeuterated solvents of different polarity.

Solvent	Concentration (mg/mL)	*D*_solv,H_ ^1^ × 10^9^ (m^2^/s)	*η* ^2^ (mPas)	*D*_ZnDBP_ × 10^9^ (m^2^/s)	*R*_H,ZnDBP_ (nm)
CDCl_3_	1	1.772	0.604	0.361	1.0
	10	1.655	0.646	0.165	2.0
C_6_D_6_	1	1.469	0.665	0.163	2.0
CD_3_OD	1	1.467	0.624	0.501	0.7

^1^ diffusion coefficient ascribed to the residual solvent peak (protonated molecules of the solvent) in the respective ZnDBP solution. ^2^ viscosity of the ZnDBP solution calculated from the equation $\eta =\left(D_{1}/D_{\mathrm{solv},H}\right)\times \eta_{1}$ in which $D_{1}$ and $\eta_{1}$ are the diffusion coefficient of the solvent’s protonated molecules in pure perdeuterated solvent and viscosity of the latter, respectively. The values of $\eta_{1}$ are taken from [48] (CDCl_3_) and [49] (benzene-d_6_, methanol-d_4_).

**Table 4:** The results of ^1^H DOSY NMR measurements carried out at 25 °C for the 5 mg/mL solutions of the ZnDOPs-based hybrid copolymers in C_6_D_6_.

Hybrid Copolymer ^1^	*D*_solv,H_ ^2^ × 10^9^ (m^2^/s)	*η* ^3^ (mPas)	*D*_cop_ × 10^9^ (m^2^/s)	*R*_H_,_cop_ (nm)
ZnDMP-*co*-ZnDEP	1.360	0.719	0.192	1.6
ZnDBP-*co*-ZnBEHP	1.360	0.719	0.047	6.5
ZnDBP-*co*-ZnDPhP	1.310	0.746	0.120	2.4

^1^ molar ratio of the monomeric units of each type is 1:1. ^2^ diffusion coefficient ascribed to the residual solvent peak (protonated molecules of C_6_H_6_) in the respective copolymer solution. ^3^ viscosity of the hybrid copolymer solution calculated from the equation $\eta =\left(D_{1}/D_{\mathrm{solv},H}\right)\times \eta_{1}$, in which $D_{1}$ and $\eta_{1}$ are the diffusion coefficient of C_6_H_6_ molecules in pure perdeuterated solvent and viscosity of the latter, respectively (*η*_1_ = 0.636 mPas based on the data from [49]).

## Data Availability

The data presented in this study are available in the article and Supplementary Material.

## Supplementary Materials

The following supporting information can be downloaded at: https://www.mdpi.com/article/10.3390/polym14163407/s1, Details of ZnDBP crystal structure determination combined with the additional references [50,51,52,53]; Details of di-*n*-propyl phosphate (DnPP) synthesis; Table S1: Elemental analysis of the investigated ZnDOPs; Table S2: Cell parameters (*a*, *b* and *c*) and cell volume (*V*) calculated from the VT-PXRD patterns collected during heating of the ZnDMP sample from 25 to 105 °C; Figure S1: FTIR spectra of ZnDOPs; Figures S2–S5: ^1^H and ^31^P NMR spectra of ZnDMP, ZnDEP, ZnDnPP and ZnDBP, respectively, recorded in DMSO-d_6_ at room temperature; Figure S6: ^1^H and ^31^P NMR spectra of ZnBEHP recorded in CDCl_3_ at room temperature; Figure S7: SEM images of the as-synthesized samples of ZnDMP and ZnDEP; Figure S8: Experimental PXRD pattern of ZnDEP recorded at room temperature and PXRD pattern simulated from a single–crystal X–ray measurement; Figure S9: α-ZnDMP PXRD pattern simulated from a single-crystal X-ray measurement and experimental PXRD patterns of ZnDMP recorded at room temperature; Figure S10: PXRD pattern of ZnDnPP recorded at room temperature; Figure S11: PXRD pattern simulated based on ZnDBP structure derived from a single–crystal X–ray measurement, and experimental PXRD patterns recorded at room temperature for different ZnDBP samples; Figure S12: PXRD pattern of ZnBEHP recorded at room temperature; Figures S13 and S14: SEM images of ZnDBP and ZnBEHP, respectively; Figures S15–S19: Simultaneous thermal analysis (STA) of ZnDMP, ZnDEP, ZnDnPP, ZnDBP and ZnBEHP, respectively, in argon, coupled with quadrupole mass spectrometry (QMS) of the evolved gases; Figure S20: VT-PXRD patterns of ZnDMP recorded at different temperatures; Figure S21: PXRD patterns of the ZnDMP samples subjected to thermal conditioning; Figure S22: DSC traces of ZnDEP recorded between −100 °C and room temperature; Figure S23: VT-PXRD patterns of ZnDEP recorded below room temperature, during cooling and subsequent heating steps; Figure S24: Location of (401), (200) and (110) diffraction planes within the ZnDEP structure; Figure S25: DSC traces of the pristine samples of ZnDnPP, ZnDBP and ZnBEHP recorded in the cooling→heating→cooling→heating mode; Figures S26 and S27: VT-PXRD traces of ZnDnPP and ZnBEHP, respectively, recorded below room temperature, during cooling and subsequent heating steps; Figure S28: ^31^P NMR spectra of ZnDBP solutions in CDCl_3_ recorded at room temperature for different sample’s mass concentrations (5–15 mg/mL); Figures S29 and S30: ^1^H and ^31^P NMR spectra of ZnDBP solution in benzene-d_6_ and methanol-d_4_, respectively; Figure S31: ^1^H DOSY NMR spectrum of the ZnDMP–*co*–ZnDEP copolymer (50 mol% of ZnDMP monomeric units) dissolved in benzene-d_6_; Figure S32: ^1^H DOSY NMR spectrum of the ZnDBP–*co*–ZnDPhP copolymer (50 mol% of ZnDPhP monomeric units) dissolved in benzene-d_6_; Figure S33: ^1^H DOSY NMR spectrum of the ZnDBP–*co*–ZnBEHP copolymer (50 mol% of ZnDBP monomeric units) dissolved in benzene-d_6_; Figure S34: ^1^H NMR spectra of different fractions of the ZnDBP-*co*-ZnDPhP copolymer (50 mol% of ZnDPhP monomeric units) dissolved in DMSO-d_6_; Figure S35: ^31^P NMR spectrum of the ZnDBP-*co*-ZnDPhP copolymer (50 mol% of ZnDPhP monomeric units) recorded in benzene-d_6_; Figure S36: PXRD patterns of the ZnDBP-*co*-ZnDPhP copolymers containing different amounts of aromatic monomeric units; Figure S37: DSC traces recorded during isothermal heating of the ZnDEP/BADGE composition (20 wt% of ZnDEP load) at different temperatures; Figure S38: PXRD patterns of the ZnDMP/BADGE compositions before and after curing at different temperatures; Figure S39: PXRD patterns of the ZnDPhP/BADGE compositions before and after curing at 160 °C.
