# Supplementary material for: Dynamic Supramolecular Polymers Based on Zinc Bis(diorganophospate)s: Synthesis, Structure and Transformations in Solid State and Solutions
Source: Polymers (Basel). 2022 Aug 20;14(16):3407. doi: 10.3390/polym14163407 (PMC9412697; doi:10.3390/polym14163407)
Supplement: Supplementary file 1 [file polymers-14-03407-s001.zip › polymers-1867657-supplementary.pdf]

Supplementary Materials

Dynamic Supramolecular Polymers Based on Zinc Bis(diorganophospate)s: Synthesis, Structure and Transformations in Solid State and Solutions

Maciej Dębowski *, Zbigniew Florjańczyk, Katarzyna Godlewska, Alicja Kaczmarczyk, Maciej Dranka and Andrzej Ostrowski

Faculty of Chemistry, Warsaw University of Technology, Noakowskiego 3, 00-664 Warsaw, POLAND

TABLE OF CONTENT

Component	Short Description	Page
	Details of the ZnDBP crystal structure determination	S3
	Details of di-*n*-propyl phosphate (DnPP) synthesis	S3
Table S1	Elemental analysis of the investigated ZnDOPs	S4
Table S2	Cell parameters (*a*, *b* and *c*) and cell volume (*V*) calculated from the VT-PXRD patterns collected during heating of the ZnDMP sample from 25 °C to 105 °C	S4
Figure S1	FTIR spectra of ZnDOPs	S5
Figure S2	^{1}H and ^{31}P NMR spectra of ZnDMP recorded in DMSO-d_6 at room temperature	S6
Figure S3	^{1}H and ^{31}P NMR spectra of ZnDEP recorded in DMSO-d_6 at room temperature	S7
Figure S4	^{1}H and ^{31}P NMR spectra of ZnDnPP recorded in DMSO-d_6 at room temperature	S8
Figure S5	^{1}H and ^{31}P NMR spectra of ZnDBP recorded in DMSO-d_6 at room temperature	S9
Figure S6	^{1}H and ^{31}P NMR spectra of ZnDEP recorded in $CDCl_3$ at room temperature	S10
Figure S7	SEM images of the as-synthesized samples of ZnDMP and ZnDEP	S11
Figure S8	Experimental PXRD pattern of ZnDEP recorded at room temperature and PXRD pattern simulated from a single–crystal X–ray measurement	S12
Figure S9	α-ZnDMP PXRD pattern simulated from a single-crystal X-ray measurement and experimental PXRD patterns of ZnDMP recorded at room temperature	S13
Figure S10	PXRD pattern of ZnDnPP recorded at room temperature	S13
Figure S11	PXRD pattern simulated based on ZnDBP structure derived from a single–crystal X–ray measurement, and experimental PXRD patterns recorded at room temperature for different ZnDBP samples	S14
Figure S12	PXRD pattern of ZnBEHP recorded at room temperature	S14
Figure S13	SEM images of ZnDBP	S15
Figure S14	SEM images of ZnBEHP	S15
Figure S15	Simultaneous thermal analysis (STA) of ZnDMP in argon coupled with quadrupole mass spectrometry (QMS) of the evolved gases	S16
Figure S16	Simultaneous thermal analysis (STA) of ZnDEP in argon coupled with quadrupole mass spectrometry (QMS) of the evolved gases	S16
Figure S17	Simultaneous thermal analysis (STA) of ZnDnPP in argon coupled with quadrupole mass spectrometry (QMS) of the evolved gases	S16
Figure S18	Simultaneous thermal analysis (STA) of ZnDBP in argon coupled with quadrupole mass spectrometry (QMS) of the evolved gases	S17
Figure S19	Simultaneous thermal analysis (STA) of ZnBEHP in argon coupled with quadrupole mass spectrometry (QMS) of the evolved gases	S17
Figure S20	VT-PXRD patterns of ZnDMP recorded at different temperatures	S18
Figure S21	PXRD patterns of the ZnDMP samples subjected to thermal conditioning	S19
Figure S22	DSC traces of ZnDEP recorded between −100 °C and room temperature	S19

corresponding author: maciej.debowski@pw.edu.pl

Figure S23	VT-PXRD patterns of ZnDEP recorded below room temperature, during cooling and subsequent heating steps	S20
Figure S24	Location of (401), (200) and (110) diffraction planes within the ZnDEP structure	S21
Figure S25	DSC traces of the pristine samples of ZnDnPP, ZnDBP and ZnBEHP recorded in the cooling→heating→ cooling→heating mode	S22
Figure S26	VT-PXRD traces of ZnDnPP recorded below room temperature, during cooling and subsequent heating steps	S23
Figure S27	VT-PXRD patterns of ZnBEHP recorded below room temperature, during cooling and subsequent heating steps	S24
Figure S28	^{31}P NMR spectra of ZnDBP solutions in $CDCl_3$ recorded at room temperature for different sample's mass concentrations (5–15 mg/mL)	S24
Figure S29	^{1}H and ^{31}P NMR spectra of ZnDBP solution in benzene-d_6	S25
Figure S30	^{1}H and ^{31}P NMR spectra of ZnDBP solution in methanol-d_4	S25
Figure S31	^{1}H DOSY NMR spectrum of the ZnDMP–*co*–ZnDEP copolymer (50 mol% of ZnDMP monomeric units) dissolved in benzene-d_6	S26
Figure S32	^{1}H DOSY NMR spectrum of the ZnDBP–*co*–ZnDPhP copolymer (50 mol% of ZnDPhP monomeric units) dissolved in benzene-d_6	S27
Figure S33	^{1}H DOSY NMR spectrum of the ZnDBP–*co*–ZnBEHP copolymer (50 mol% of ZnDBP monomeric units) dissolved in benzene-d_6	S28
Figure S34	^{1}H NMR spectra of different fractions of the ZnDBP-*co*-ZnDPhP copolymer (50 mol% of ZnDPhP monomeric units) dissolved in DMSO-d_6	S29
Figure S35	^{31}P NMR spectrum of the ZnDBP-*co*-ZnDPhP copolymer (50 mol% of ZnDPhP monomeric units) recorded in benzene-d_6	S30
Figure S36	PXRD patterns of the ZnDBP-*co*-ZnDPhP copolymers containing different amounts of aromatic monomeric units	S30
Figure S37	DSC traces recorded during isothermal heating of the ZnDEP/BADGE composition (20 wt% of ZnDEP load) at different temperatures	S31
Figure S38	PXRD patterns of the ZnDMP/BADGE compositions before and after curing at different temperatures	S31
Figure S39	PXRD patterns of the ZnDPhP/BADGE compositions before and after curing at 160 °C	S32

corresponding author: maciej.debowski@pw.edu.pl

Details of ZnDBP crystal structure determination

A single crystal suitable for X-ray diffraction studies was selected under a polarizing microscope, mounted in inert oil and transferred to the cold gas stream of the Oxford Diffraction κ-CCD Gemini A Ultra diffractometer. Cell refinement and data collection, and data reduction and analysis were performed with the CRYSALIS^{PRO} software [50]. The crystal data were processed in Olex2 [51], solved with the ShelXT [52] structure solution program using Intrinsic Phasing and refined with the SHELXL–2018/3 program refinement package [53] using Least Squares minimization.
ZnDBP crystallizes from a methanol/DMSO solution as a 1D coordination polymer in triclinic space group $P-1$ in the form of colourless needles.
Crystal Data for ZnDBP: space group: triclinic, space group $P-1$, a = 9.1749(10) Å, b = 15.314(2) Å, c = 19.409(2) Å, α = 72.004(12)°, β = 89.213(9)°, γ = 87.397(10)°, V = 2590.9(6) Å^3, Z = 4, T = 291.0(1) K, μ(MoKα) = 1.087 mm^{-1}, 28800 reflections measured (6.798° ≤ 2θ ≤ 61.014°), 15454 unique (R_{int} = 0.1388, R_{sigma} = 0.2643) which were used in all calculations. The final R_1 was 0.2161 ($I > 2\sigma(I)$) and wR_2 was 0.6005 (all data).

References
50. CRYSALISPRO Software system, Rigaku, Oxford, UK, 2022.
51. Dolomanov, O.V.; Bourhis, L.J.; Gildea, R.J; Howard, J.A.K.; Puschmann, H. OLEX2: a complete structure solution, refinement and analysis program. *J. Appl. Cryst.* **2009**, *42*, 339–341.
52. Sheldrick, G.M. SHELXT - Integrated space-group and crystal-structure determination. *Acta Cryst.* **2015**, *A71*, 3–8.
53. Sheldrick, G.M. Crystal Structure Refinement with SHELXL. *Acta Cryst.* **2015**, *C71*, 3–8.

Details of di-*n*-propyl phosphate (DnPP) synthesis

The reaction was carried as follows: to a 100 mL round-bottom flask containing $CaCl_2$ (18.75 g, 163.9 mmol) and equipped in a 3-way distillation adapter connected to a Liebig condenser, liquid TnPP (25.00 g, 110.4 mmol) was added and the reagents were thoroughly mixed with magnetic stirring bar. The flask was placed in an oil bath preheated to 120 °C and the reaction was carried out at that temperature for 20 h. After cooling to room temperature, the post-reaction mixture was mixed with water and filtered through a cellulose filter. The aqueous filtrate was washed several times with CH_2Cl_2 and concentrated on a rotary evaporator resulting in 44.37 g of a solid product – crude calcium bis(di-*n*-propylphosphate) (CaDnPP).
The entire crude CaDnPP was placed in a 250 mL round-bottom flask and for 12 hours thoroughly mixed with 100 mL of CH_2Cl_2. To the resulting dispersion, after cooling it to *ca.* −10 °C, 19.53 g of 85% H_2SO_4 was added dropwise and the reaction was carried out for 30 minutes. Afterwards, the post-reaction mixture was separated on a Büchner funnel and the insoluble fraction was washed 2 times with 50 mL of CH_2Cl_2. The filtrate was dried over an anhydrous $MgSO_4$ and concentrated on a rotary evaporator resulting in 16.66 g of a liquid product, DnPP.
The overall reaction yield, calculated based on the amount of TnPP, was approximately 83%.

corresponding author: maciej.debowski@pw.edu.pl

Table S1. Elemental analysis of the investigated zinc bis(diorganophosphate)s

Compound (ZnDOP) [1]	Compound's formula (theoretical values) [2]	Results of elemental analysis		
		C (wt%)	H (wt%)	Zn (wt%)
ZnDMP	$C_4H_{12}O_8P_2Zn$ (C, 15.23%; H, 3.83%; Zn, 20.73%)	15.27	3.97	20.6
ZnDEP	$C_8H_{20}O_8P_2Zn$ (C, 25.83%; H, 5.42%; Zn, 17.60%)	25.79	5.44	17.7
ZnDnPP	$C_{12}H_{28}O_8P_2Zn$ (C, 33.70%; H, 6.60%; Zn, 15.29%)	33.22	6.21	15.6
ZnDBP	$C_{16}H_{36}O_8P_2Zn$ (C, 39.72%; H, 7.50%; Zn, 13.52)	39.76 [4]<br>39.88 [5]	7.41 [4]<br>7.15 [5]	nd [3]<br>13.7 [5]
ZnBEHP	$C_{32}H_{68}O_8P_2Zn$ (C, 54.27%; H, 9.68%; Zn, 9.24%)	54.83	9.20	9.1

[1] zinc bis(diorganophosphate) containing methyl (ZnDMP), ethyl (ZnDEP), *n*-propyl (ZnDnPP), *n*-butyl (ZnDBP), or 2–ethylhexyl (ZnBEHP) groups.
[2] values of carbon, hydrogen and zinc content (in wt%) calculated for the assumed compound's formula.
[3] not determined.
[4] ZnDBP not subjected to temperature above 25 °C.
[5] ZnDBP subjected to 60 °C during the last stage of synthesis.

Table S2. Cell parameters (*a*, *b* and *c*) and cell volume (*V*) calculated from the VT-PXRD patterns collected during heating of the ZnDMP sample from 25 °C to 105 °C

T (°C)	*a* (Å)	*b* (Å)	*c* (Å)	*V* (Å^3)
25	9.328	10.413	11.935	1159.1
35	9.325	10.403	11.970	1161.0
45	9.344	10.416	11.999	1167.6
55	9.342	10.400	12.043	1169.9
65	9.311	10.375	12.131	1171.7
75	9.314	10.379	12.149	1174.3
85	9.300	10.376	12.189	1176.1
95	9.303	10.377	12.209	1178.5
105	9.301	10.381	12.230	1180.7

corresponding author: maciej.debowski@pw.edu.pl

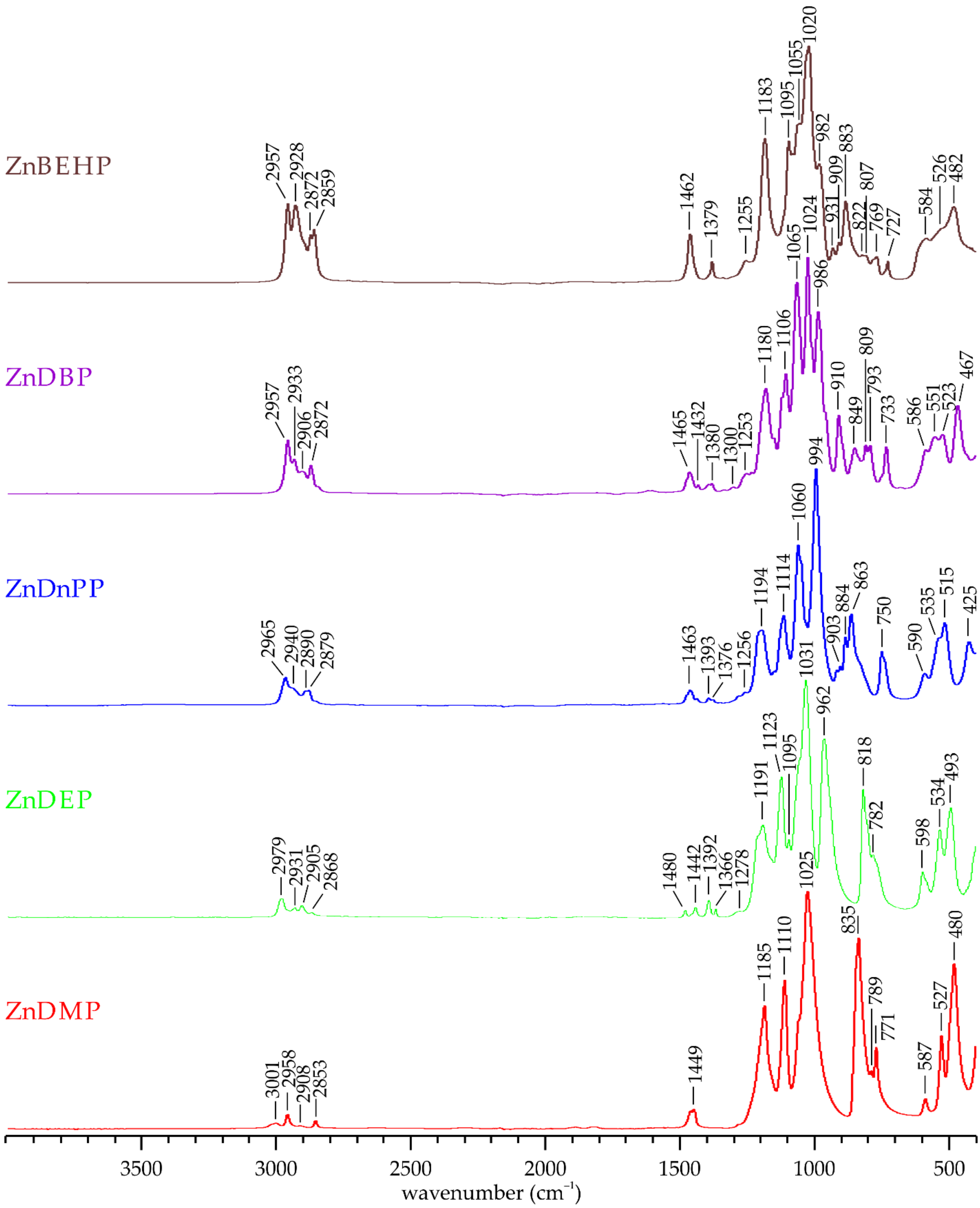

Figure S1. FTIR spectra of ZnDOPs.

corresponding author: maciej.debowski@pw.edu.pl

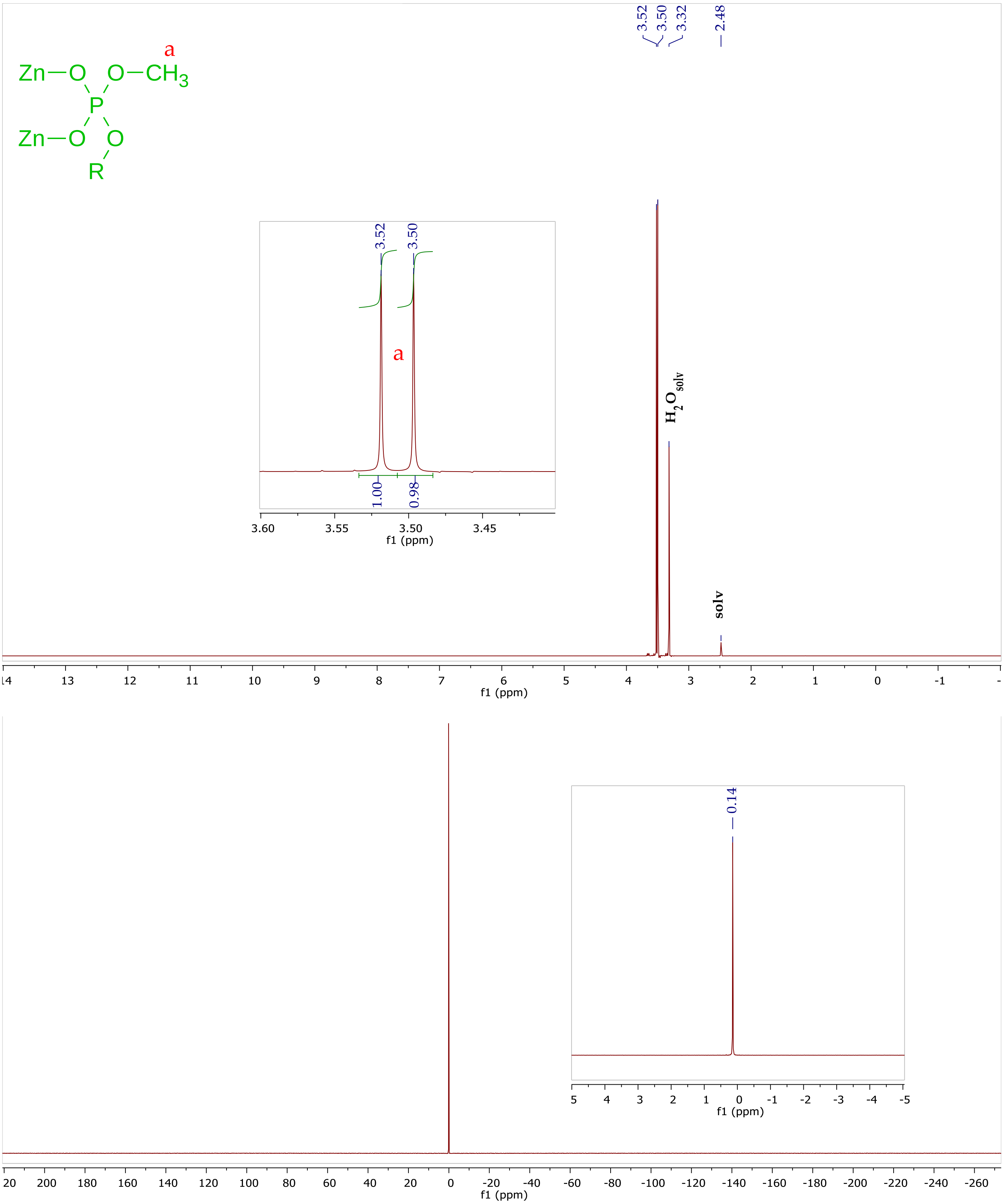

Figure S2. ^{1}H (top) and ^{31}P (down) NMR spectra of ZnDMP solution in DMSO-d_6 (mass concentration of 1 mg/mL) recorded at room temperature. Abbreviations: solv – solvent residual peak, H_2O_{solv} – water present in solvent.

corresponding author: maciej.debowski@pw.edu.pl

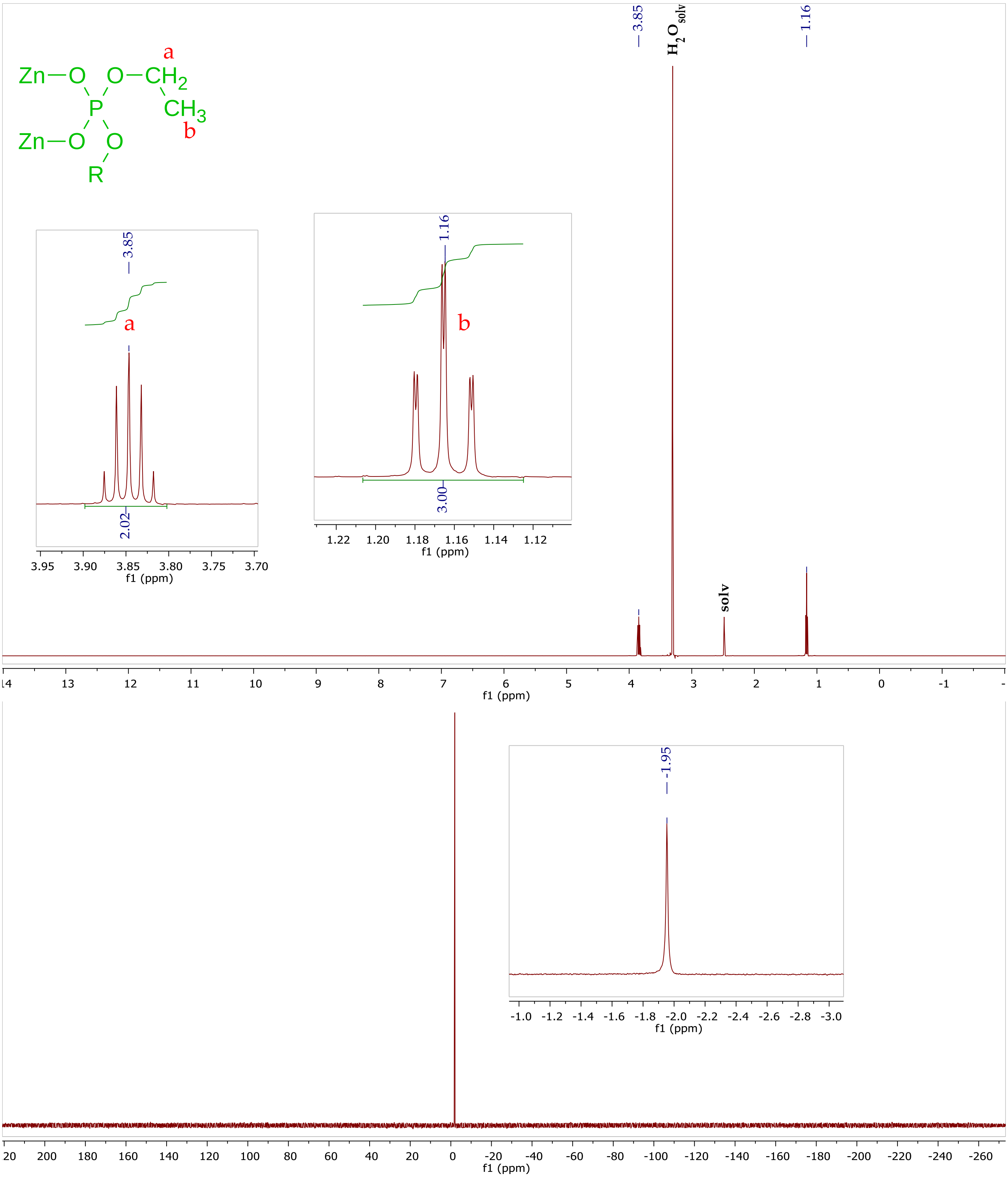

Figure S3. ^{1}H (top) and ^{31}P (down) NMR spectra of ZnDEP solution in DMSO-d$_6$ (mass concentration of 1 mg/mL) recorded at room temperature. Abbreviations: solv – solvent residual peak, H_2O_{solv} – water present in solvent.

corresponding author: maciej.debowski@pw.edu.pl

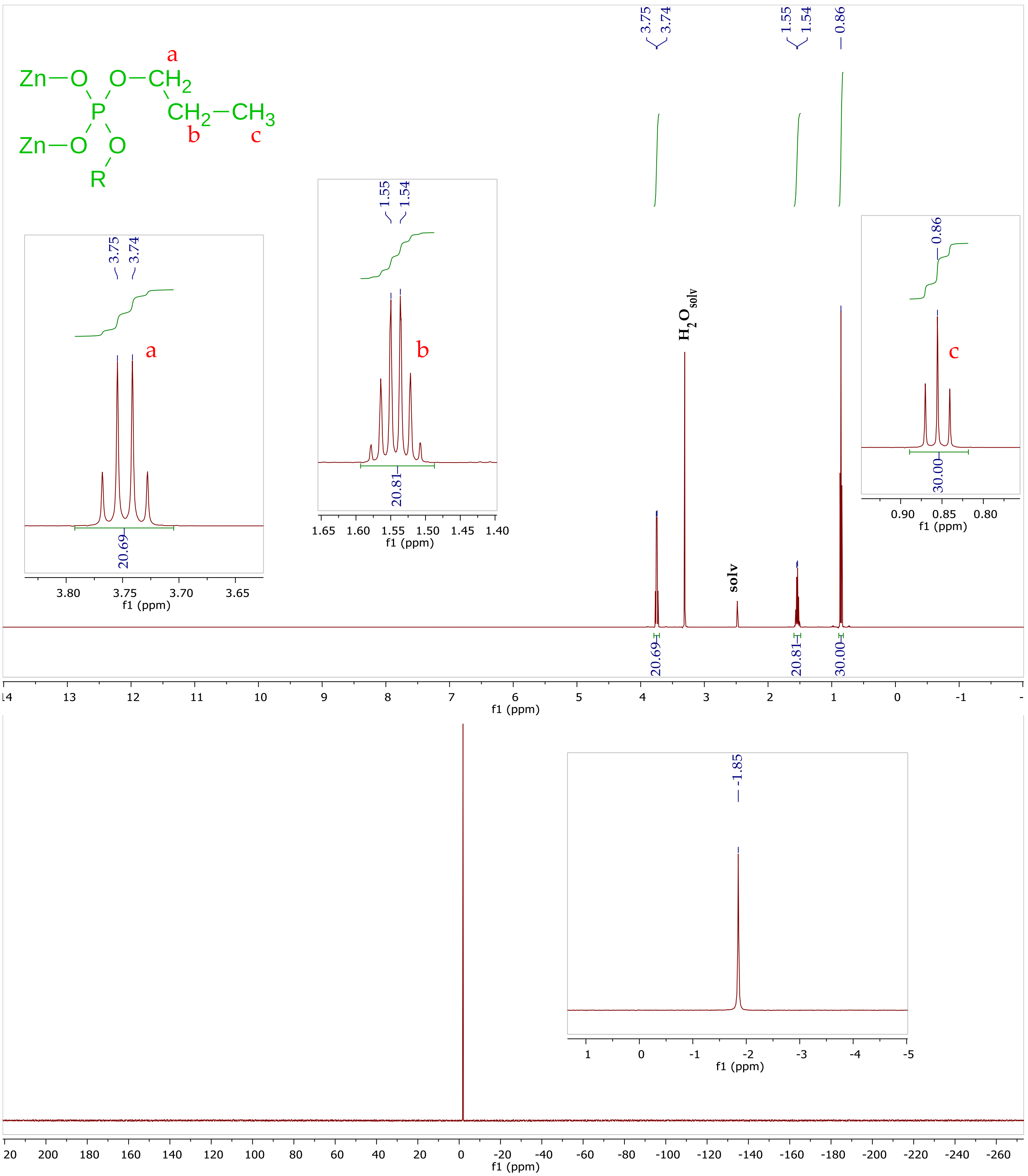

Figure S4. ^{1}H (top) and ^{31}P (down) NMR spectra of ZnDnPP solution in DMSO-d_6 (mass concentration of 1 mg/mL) recorded at room temperature. Abbreviations: solv – solvent residual peak, H_2O_{solv} – water present in solvent.

corresponding author: maciej.debowski@pw.edu.pl

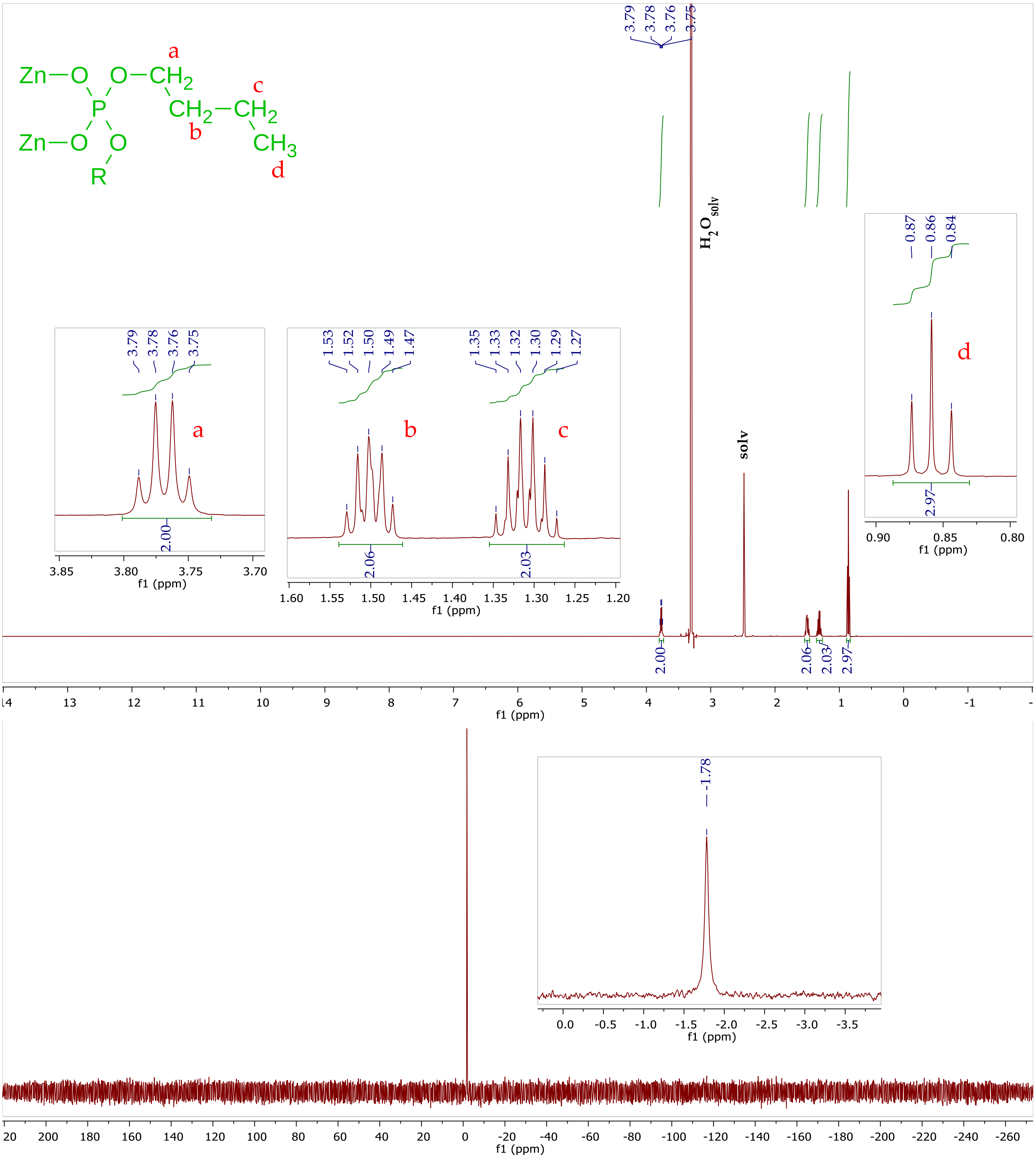

Figure S5. ^{1}H (top) and ^{31}P (down) NMR spectra of ZnDBP solution in DMSO-d_6 (mass concentration of 1 mg/mL) recorded at room temperature. Abbreviations: solv – solvent residual peak, H_2O_{solv} – water present in solvent.

corresponding author: maciej.debowski@pw.edu.pl

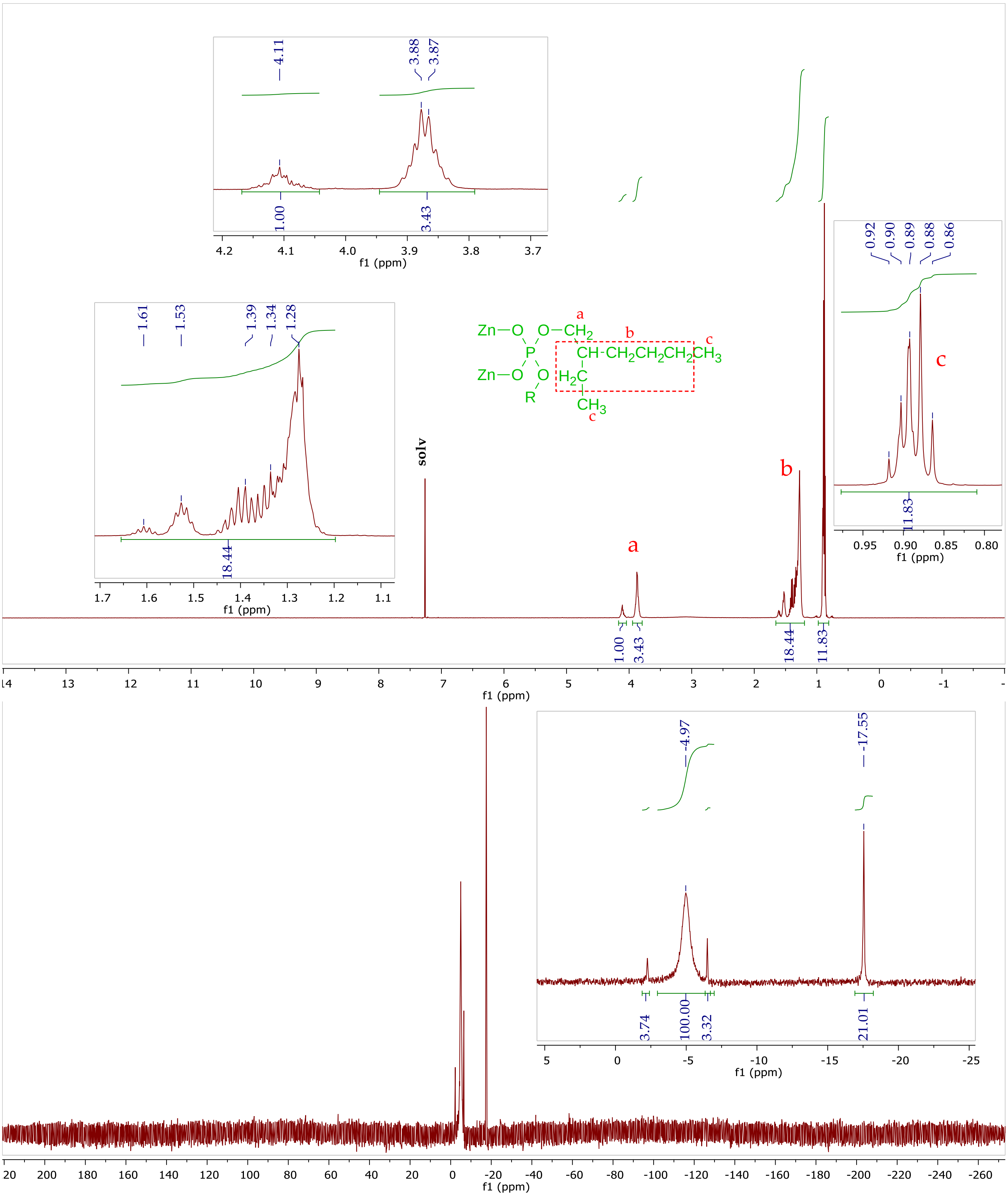

Figure S6. ^{1}H (top) and ^{31}P (down) NMR spectra of ZnBEHP solution in $CDCl_3$ (mass concentration of 1 mg/mL) recorded at room temperature. Abbreviations: solv – solvent residual peak.

corresponding author: maciej.debowski@pw.edu.pl

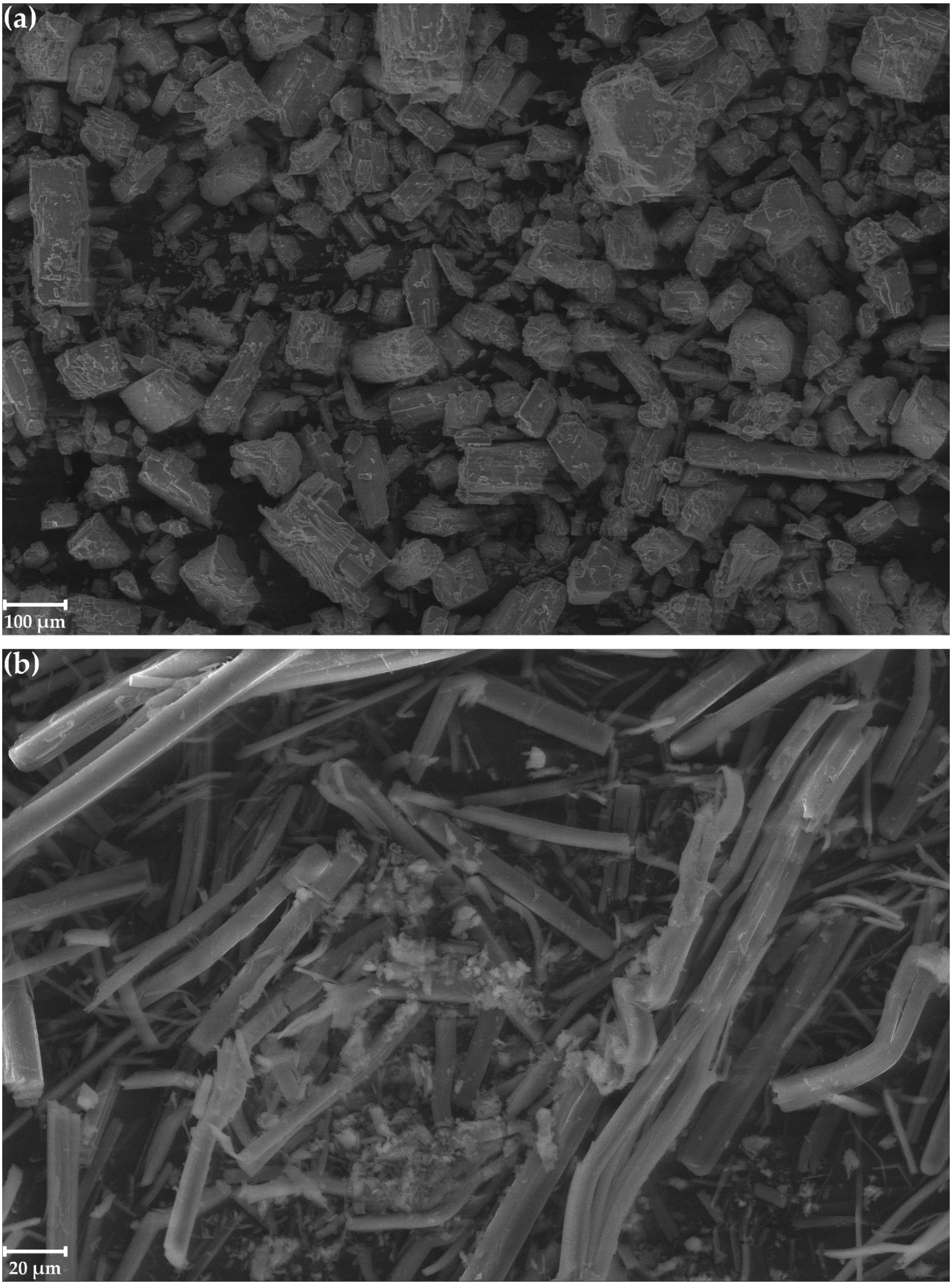

Figure S7. SEM images of the as-synthesized samples of: (a) ZnDMP and (b) ZnDEP. The images were recorded on a Prisma E instrument (Thermo Fisher Scientific Co.) with magnification of 100x (a) or 500x (b).

corresponding author: maciej.debowski@pw.edu.pl

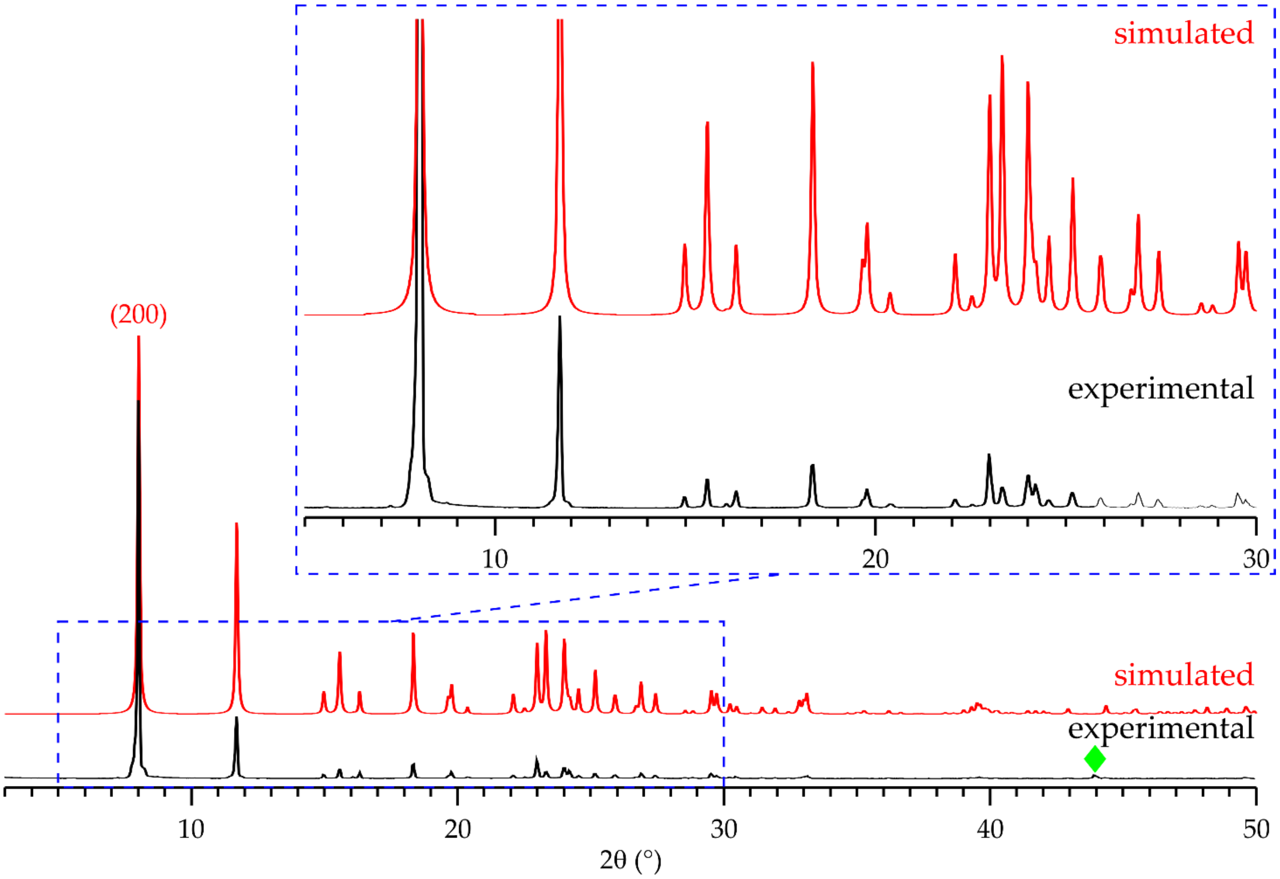

Figure S8. Experimental PXRD pattern of ZnDEP recorded at room temperature (black line) and PXRD pattern simulated from a single–crystal X–ray measurement carried out by Harrison and coworkers [16] (red line). The reflection observed for a polycrystalline sample around 44° (marked with ♦) results from diamond powder used as an internal standard. Miller index of the major reflection is denoted. The diffractograms are normalized between 0 and 1.

References

16. Harrison, W.T.A.; Nenoff, T.M.; Gier, T.E.; Stucky, G.D. Tetrahedral-atom zincophosphate structures. Zinc diethyl phosphate, $[Zn(O_2P(OC_2H_5)_2)_2]_x$, a one-dimensional inorganic "polymer". *Inorg. Chem.* **1992**, *31*, 5395–5399.

corresponding author: maciej.debowski@pw.edu.pl

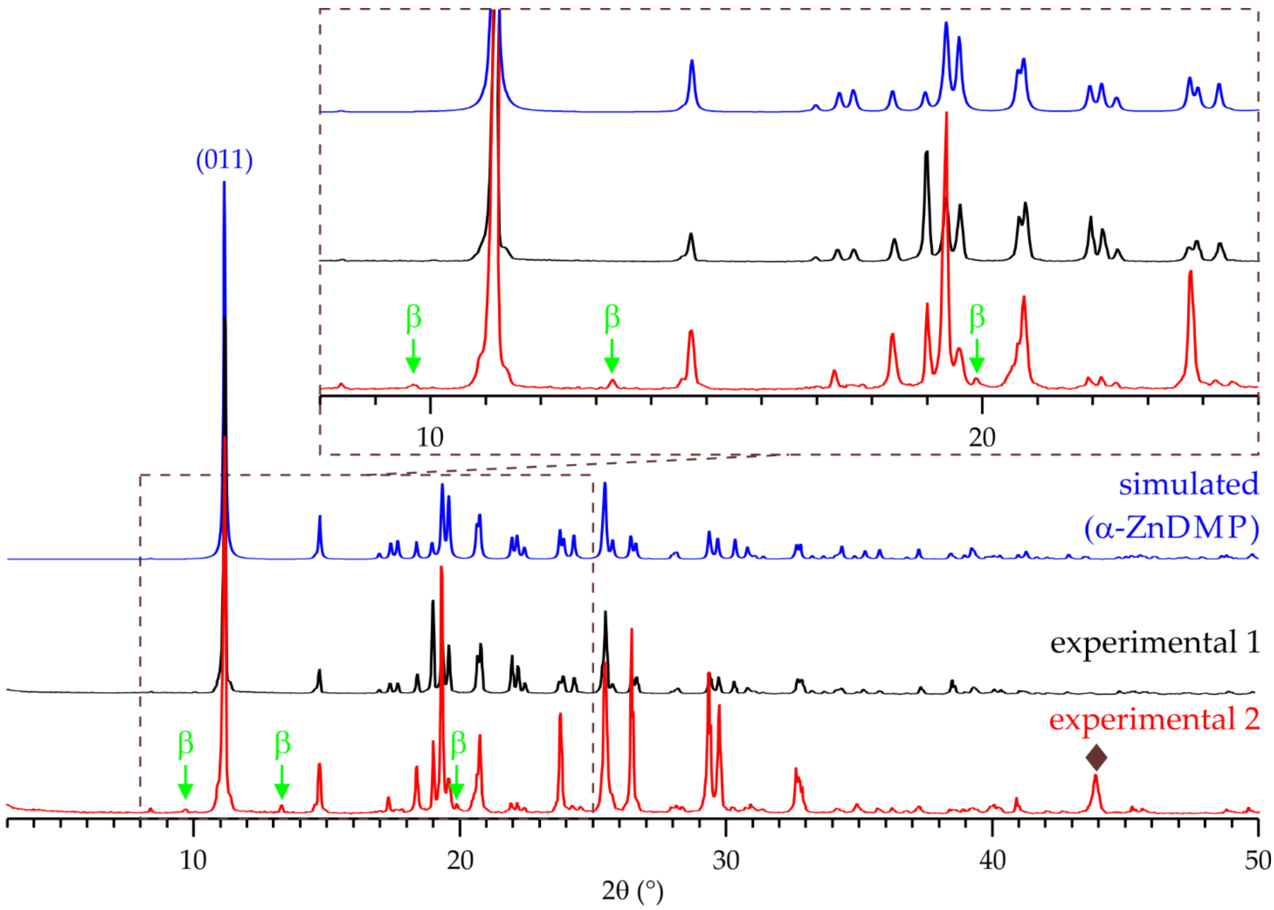

Figure S9. PXRD pattern of α-ZnDMP simulated from a single–crystal X–ray measurement carried out by Harrison and coworkers [18] (blue line) and experimental PXRD patterns of the as-synthesized ZnDMP sample (black line), or crystals formed during a slow (24 h) evaporation of ZnDMP aqueous solution at 70 °C (red line). Symbol ♦ indicates a reflection from diamond powder used as an internal standard, while some reflections of β-ZnDMP are marked with β symbol. The diffractograms are normalized between 0 and 1.

References

18. Harrison, W.T.A.; Nenoff, T.M.; Gier, T.E.; Stucky, G.D. Zinc dimethyl phosphate, $Zn[O_2P(OCH_3)_2]_2$, a one-dimensional inorganic polymer. *J. Mater. Chem.* **1994**, *4*, 1111–1115.

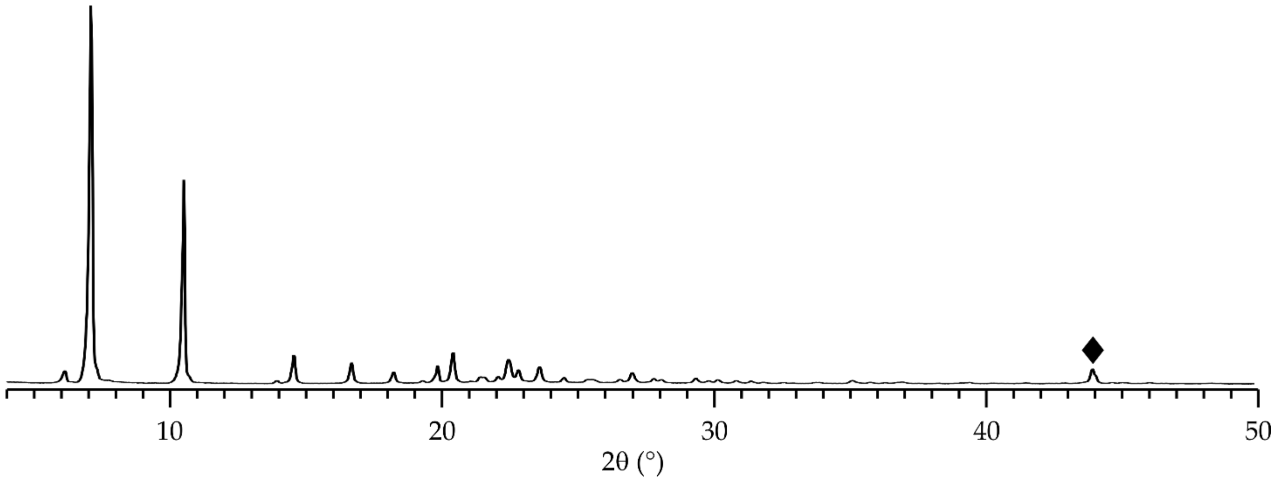

Figure S10. PXRD pattern of ZnDnPP recorded at room temperature. Symbol ♦ indicates a reflection from diamond powder used as an internal standard.

corresponding author: maciej.debowski@pw.edu.pl

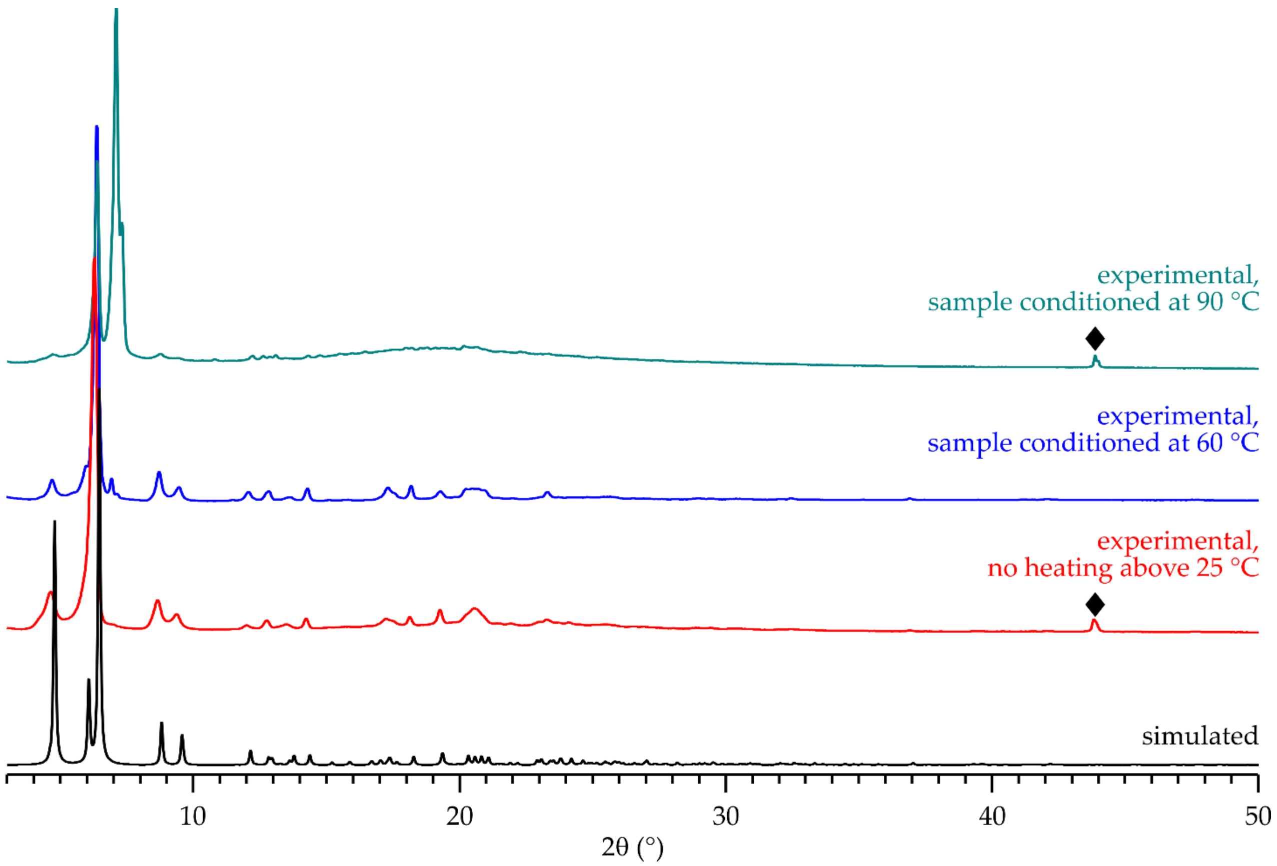

Figure S11. PXRD pattern simulated based on ZnDBP structure derived from a single–crystal X–ray measurement (black line), and experimental PXRD patterns recorded at room temperature for the ZnDBP samples obtained at 25 °C (red line), as well as subjected to heating at 60 °C (blue line) or 90 °C (green line). The diffractograms are normalized between 0 and 1. Symbol ♦ indicates a reflection from diamond powder used as an internal standard.

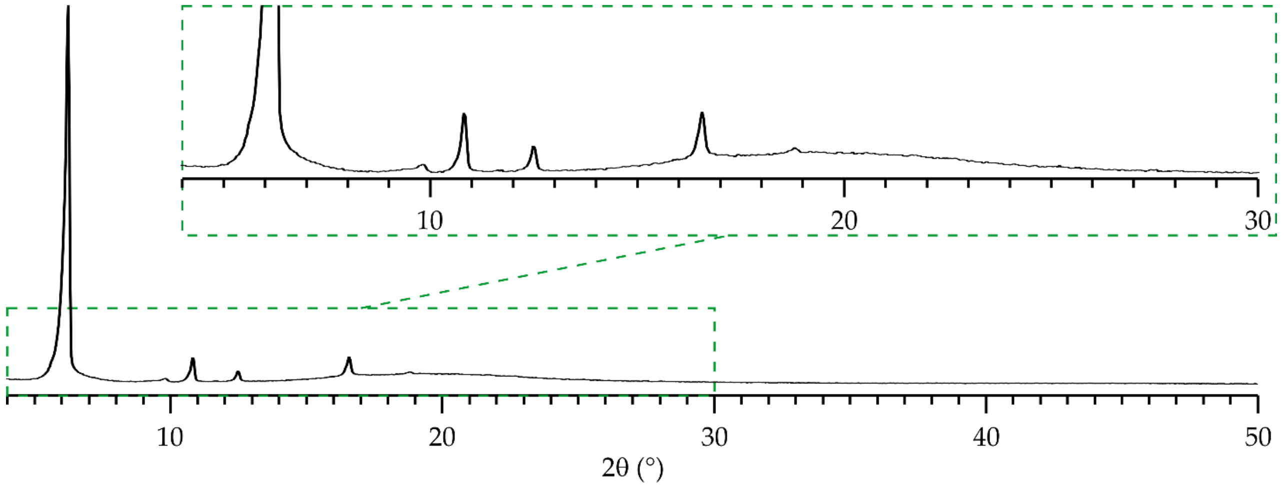

Figure S12. Experimental PXRD pattern of the ZnBEHP sample.

corresponding author: maciej.debowski@pw.edu.pl

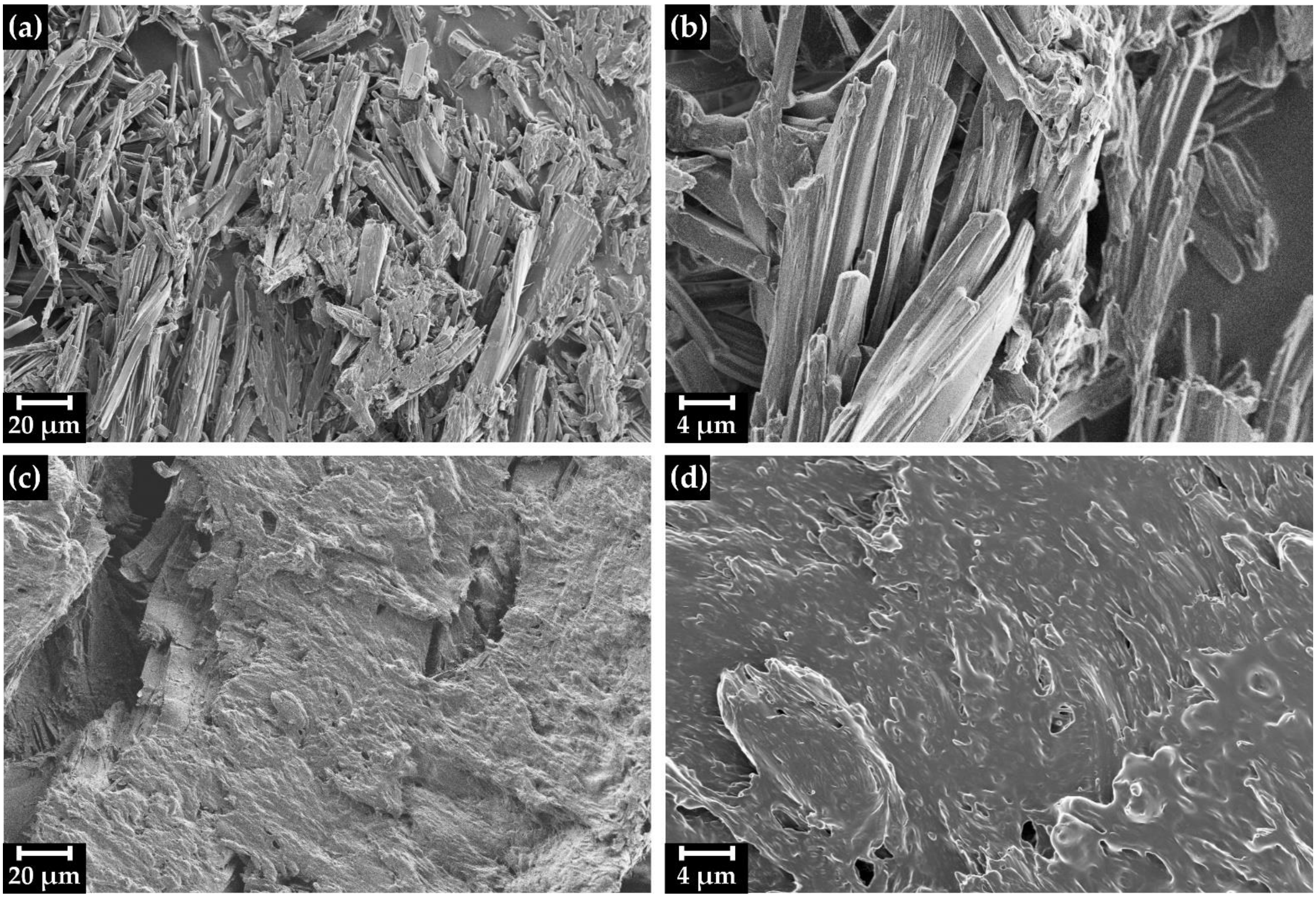

Figure S13. SEM images of ZnDBP synthesized at room temperature (a, b), or heated at 60 °C during the last stage of the synthesis (c, d). The images were recorded on a Zeiss Ultra Plus instrument with magnification of 1000x (a, c) or 5000x (b, d).

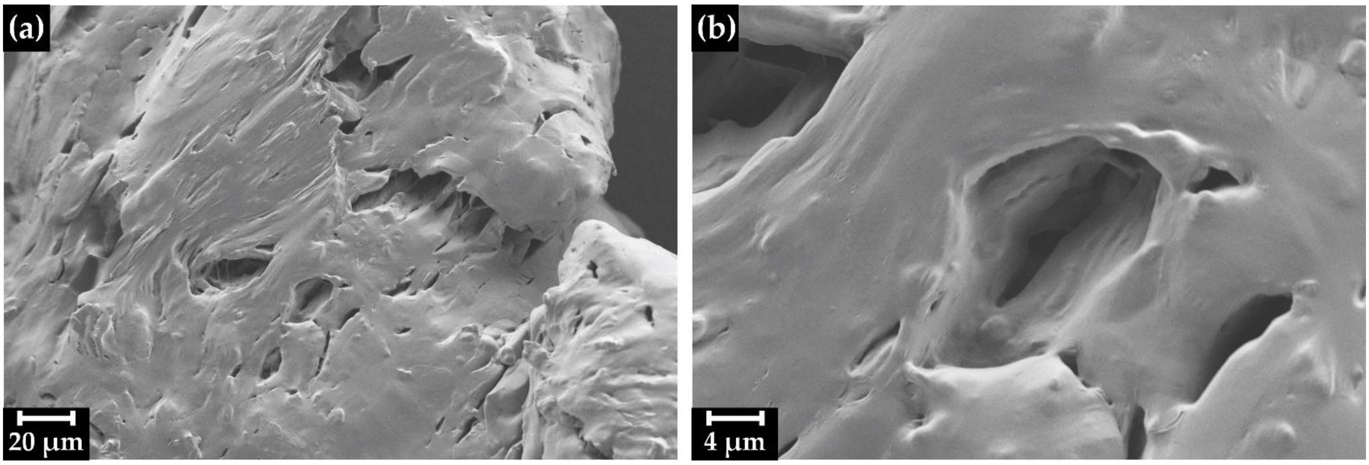

Figure S14. SEM images of ZnBEHP heated at 60°C during the last stage of the synthesis. The images were recorded on a Zeiss Ultra Plus instrument with magnification of 1000x (a, c) or 5000x (b, d).

corresponding author: maciej.debowski@pw.edu.pl

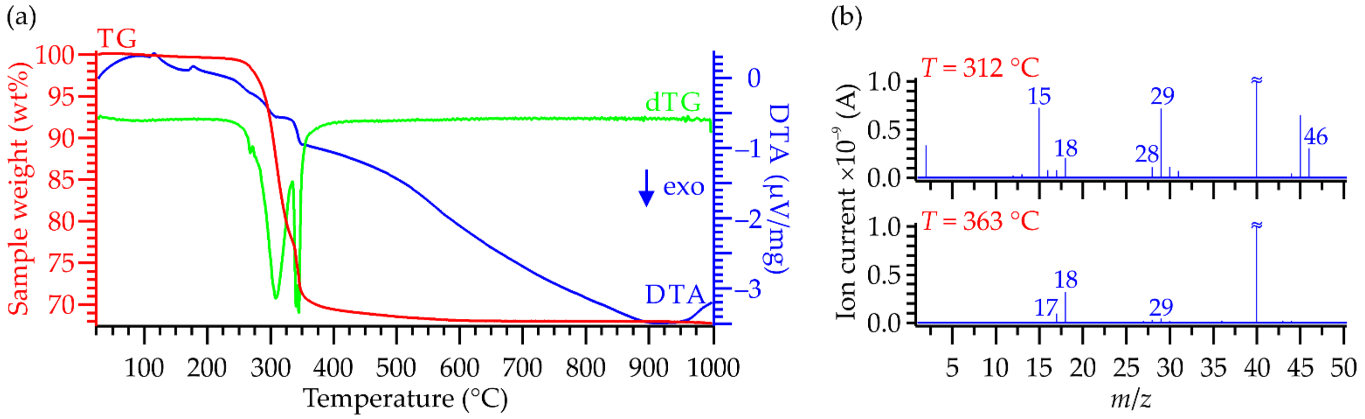

Figure S15. STA of ZnDMP carried out in argon: (a) TG (red line), dTG (green line) and DTA (blue line) curves, (b) QMS spectra of the evolved volatile products from the ZnDMP pyrolysis, taken at the specified temperatures.

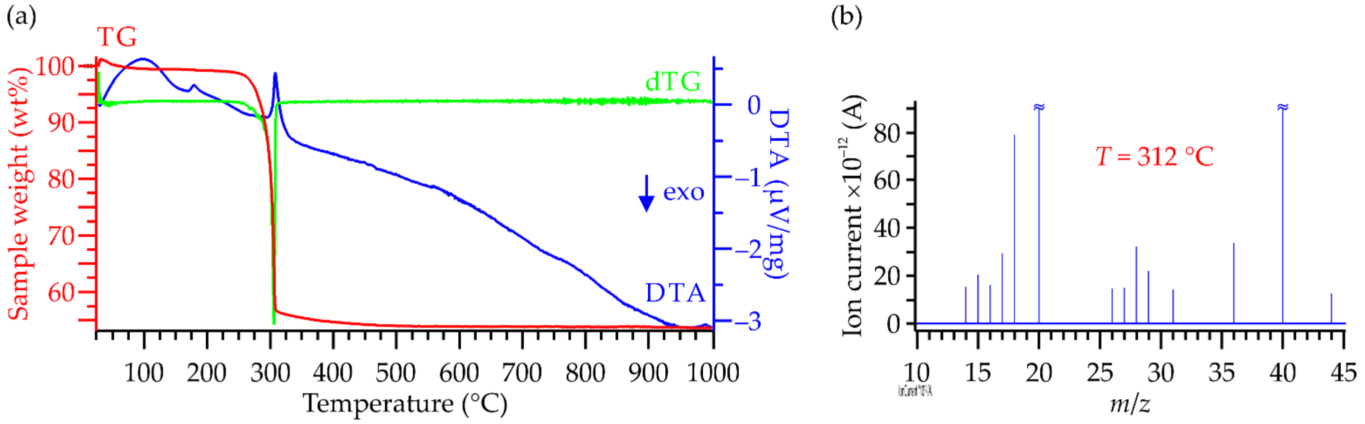

Figure S16. STA of ZnDEP carried out in argon: (a) TG (red line), dTG (green line) and DTA (blue line) curves, (b) QMS spectrum of the evolved volatile products from the ZnDEP pyrolysis, taken close to the temperature of the maximum rate of decomposition.

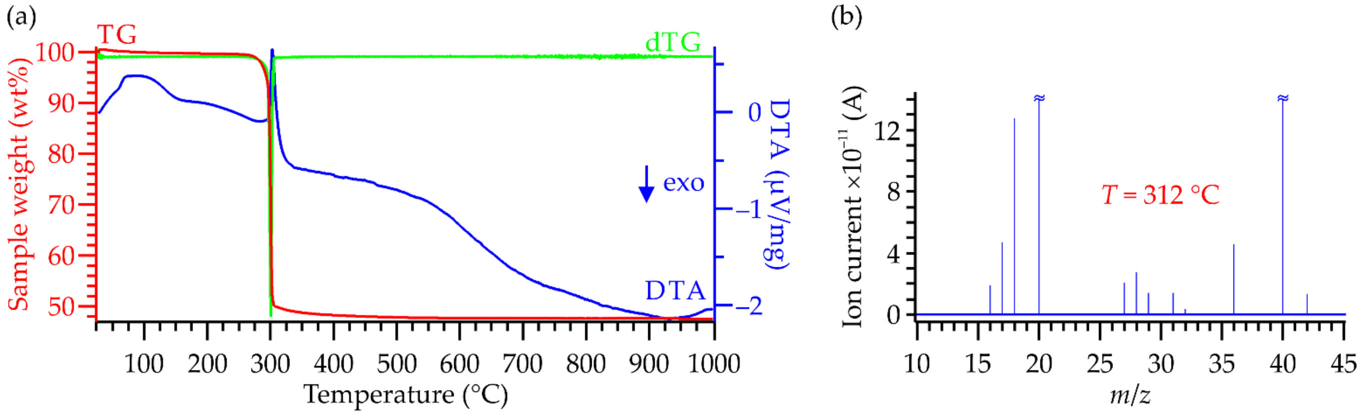

Figure S17. STA of ZnDnPP carried out in argon: (a) TG (red line), dTG (green line) and DTA (blue line) curves, (b) QMS spectrum of the evolved volatile products from the ZnDnPP pyrolysis, taken close to the temperature of the maximum rate of decomposition.

corresponding author: maciej.debowski@pw.edu.pl

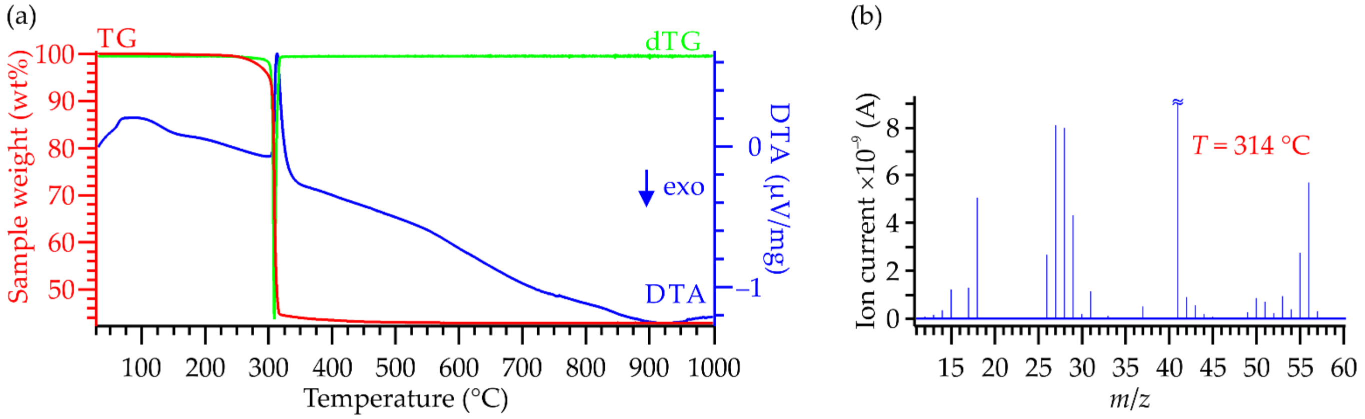

Figure S18. STA of ZnDBP carried out in argon: (a) TG (red line), dTG (green line) and DTA (blue line) curves, (b) QMS spectrum of the evolved volatile products from the ZnDBP pyrolysis, taken close to the temperature of the maximum rate of decomposition.

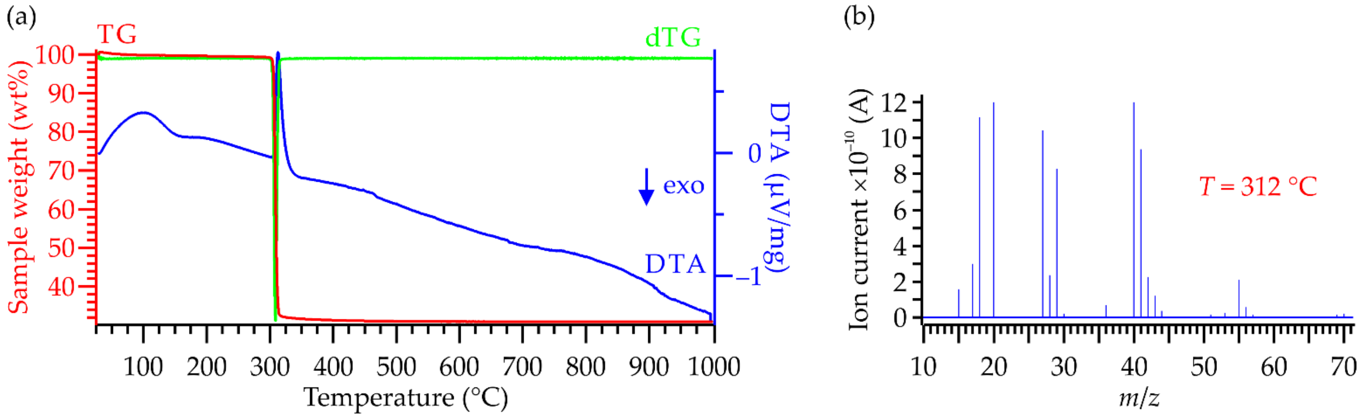

Figure S19. STA of ZnBEHP carried out in argon: (a) TG (red line), dTG (green line) and DTA (blue line) curves, (b) QMS spectrum of the evolved volatile products from the ZnBEHP pyrolysis, taken close to the temperature of the maximum rate of decomposition.

corresponding author: maciej.debowski@pw.edu.pl

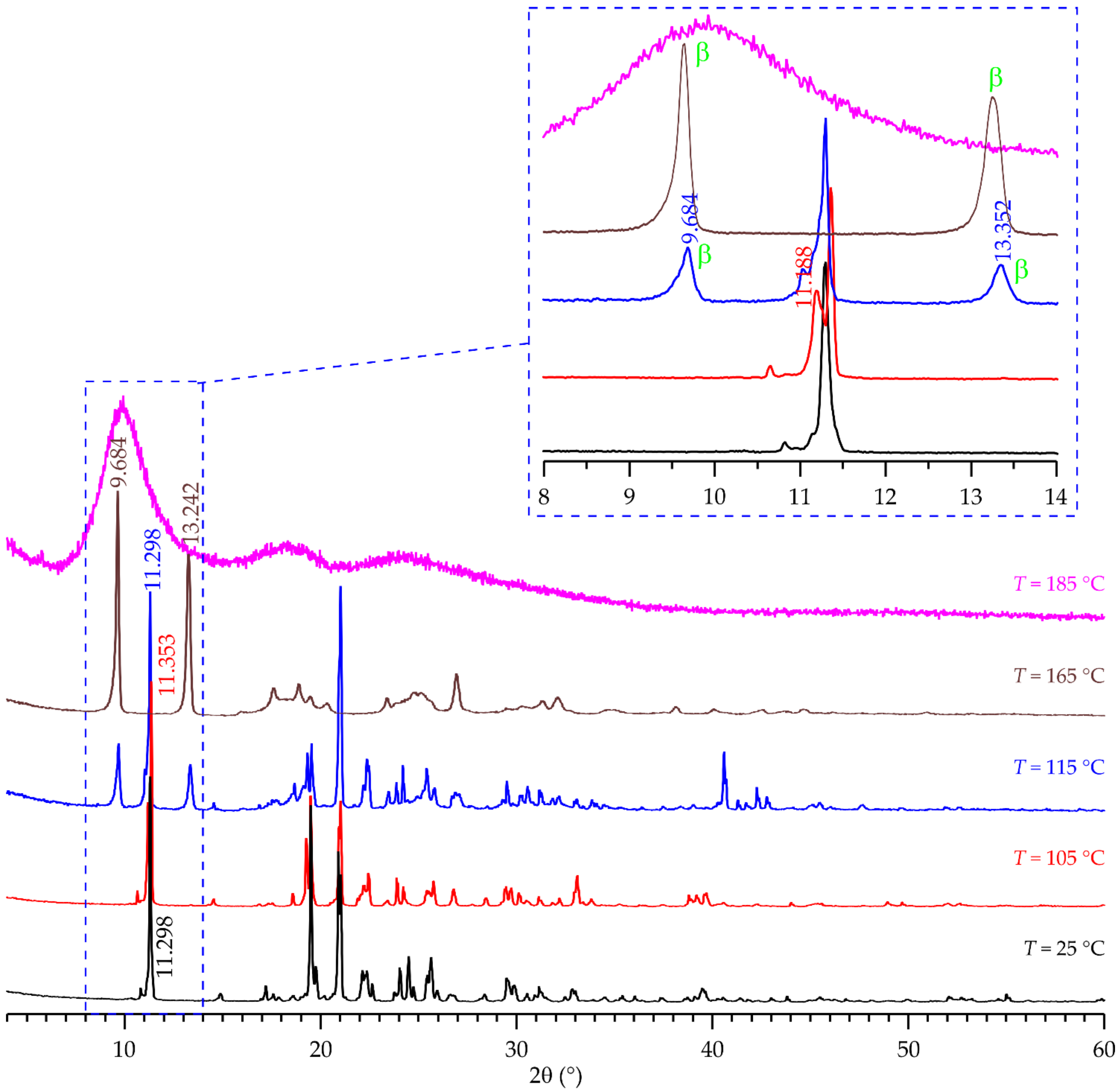

Figure S20. VT-PXRD patterns of ZnDMP recorded at the selected temperatures. The main reflections attributable to β-ZnDMP are marked with green β symbol. The diffractograms are normalized between 0 and 1.

corresponding author: maciej.debowski@pw.edu.pl

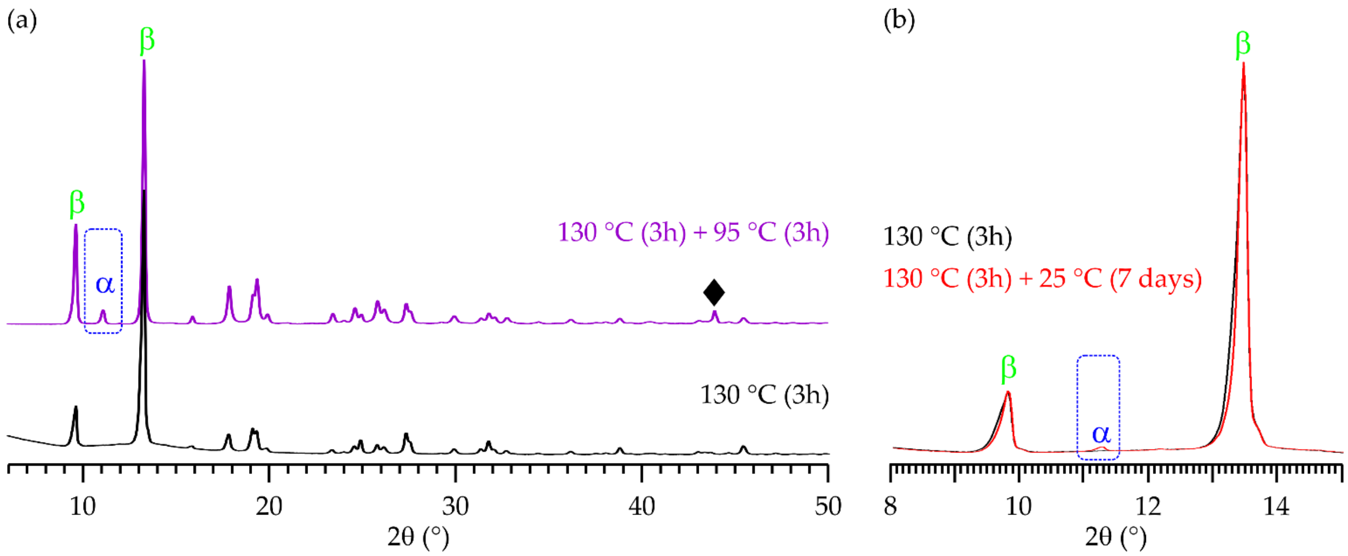

Figure S21. PXRD patterns of the ZnDMP sample: (a) heated for 3h at 130 °C (black line) and subsequently conditioned at 95 °C for 3h (purple line), (b) heated for 3h at 130 °C (black line) and subsequently conditioned at 25 °C for 7 days (red line). Symbol ♦ indicates a reflection from diamond powder used as an internal standard, while some reflections characteristic for α-ZnDMP or β-ZnDMP are marked with α or β symbols, respectively.

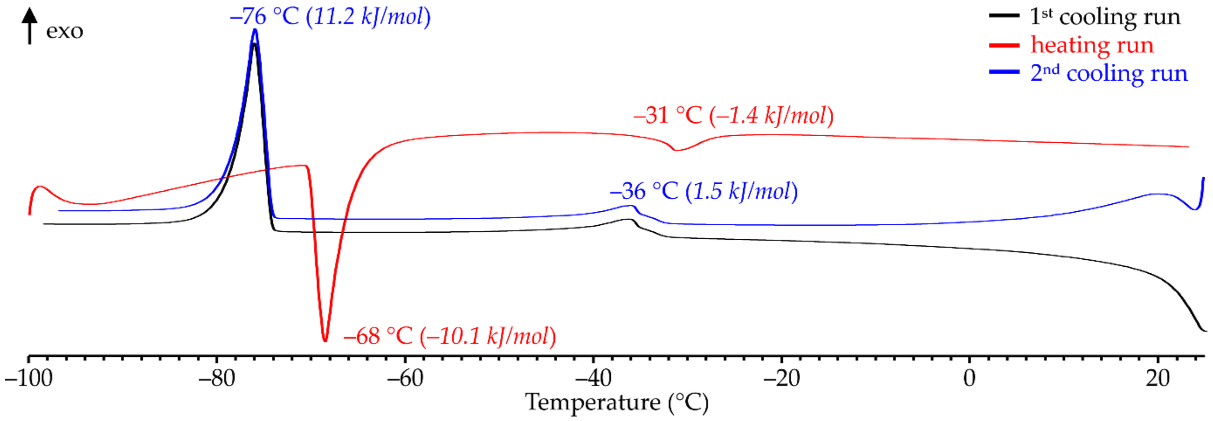

Figure S22. DSC traces of ZnDEP recorded between −100 °C and room temperature. The measurement was carried out at the 10 °C/min rate, in the following order of runs: cooling→heating→cooling.

corresponding author: maciej.debowski@pw.edu.pl

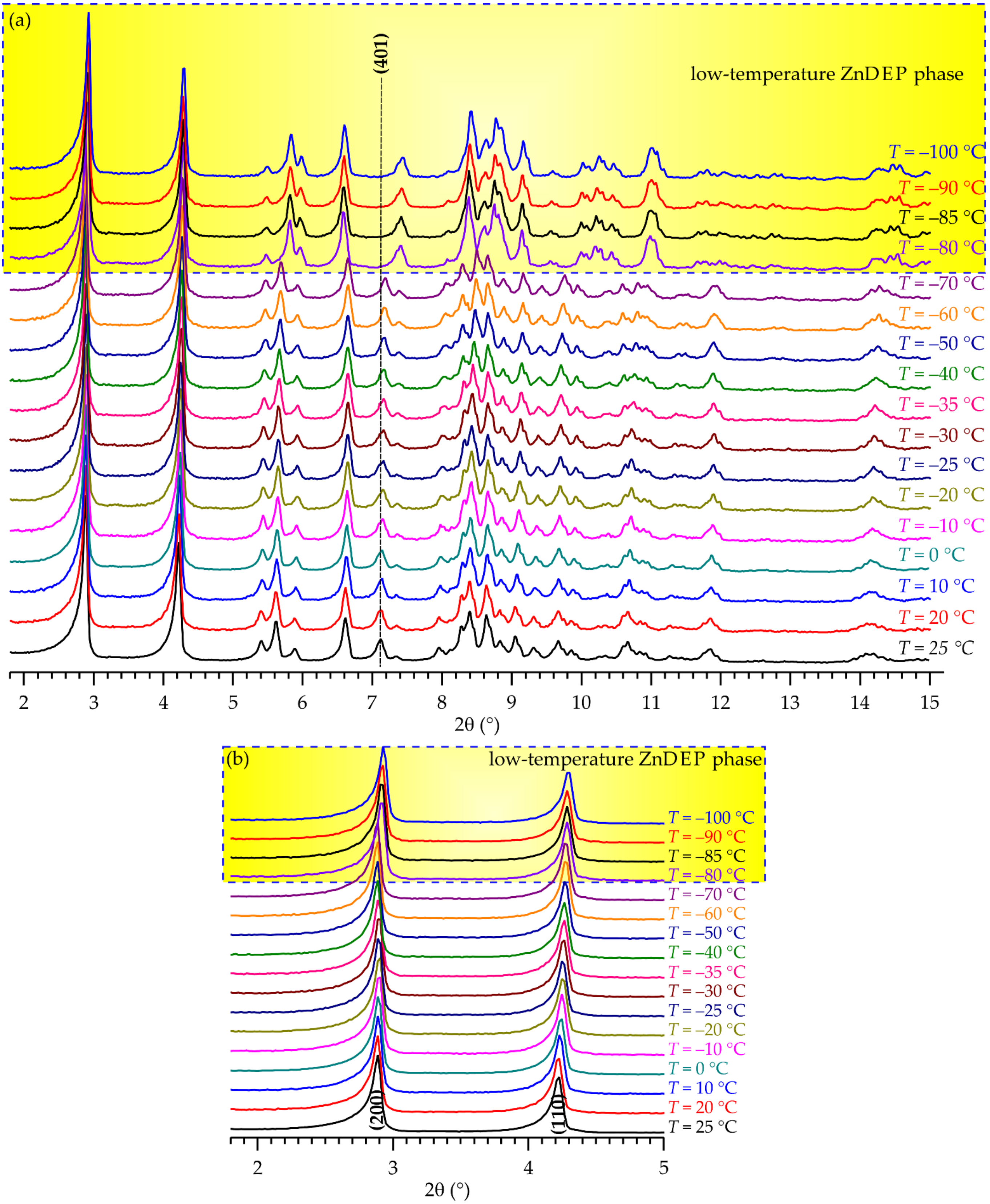

Figure S23. VT-PXRD patterns of ZnDEP recorded below room temperature (temperature of each measurement is shown): (a) full pattern, (b) the 1.8–5.0 2θ angle region. The measurements were conducted on an instrument equipped with Ag X-ray source (λ = 0.561 Å). The reflections attributable to the (200), (110) and (401) diffraction planes in ZnDEP are indicated. The diffractograms are normalized between 0 and 1.

corresponding author: maciej.debowski@pw.edu.pl

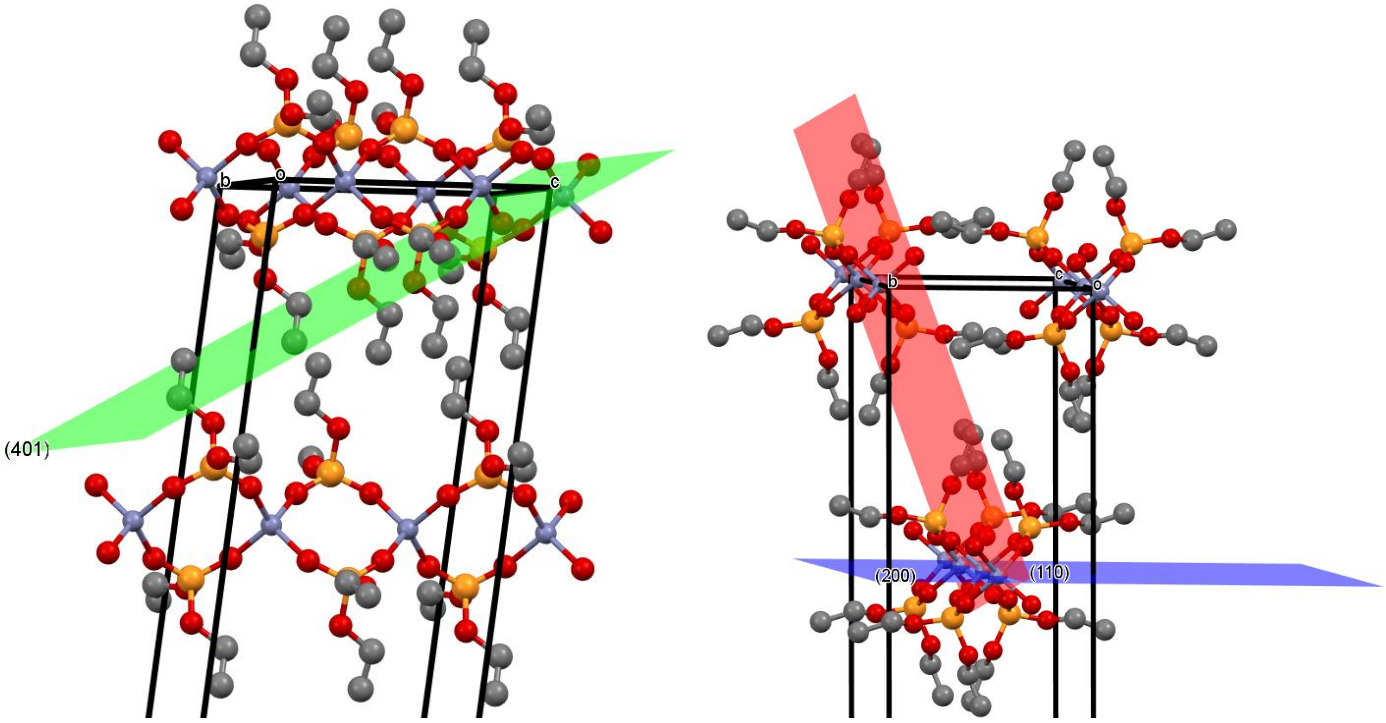

Figure S24. Location of the selected X-ray diffraction planes within the ZnDEP structure: (401) plane (green), (200) plane (dark blue) and (110) plane (red) – data from a single crystal XRD measurement at room temperature carried out by Harrison and coworkers [16]. Zinc, phosphorus, oxygen and carbon atoms are showed as dark violet, orange, red and grey spheres, respectively. Hydrogen atoms are omitted for the clarity of presentation.

References

16. Harrison, W.T.A.; Nenoff, T.M.; Gier, T.E.; Stucky, G.D. Tetrahedral-atom zincophosphate structures. Zinc diethyl phosphate, $[Zn(O_2P(OC_2H_5)_2)_2]_x$, a one-dimensional inorganic "polymer". *Inorg. Chem.* **1992**, *31*, 5395–5399.

corresponding author: maciej.debowski@pw.edu.pl

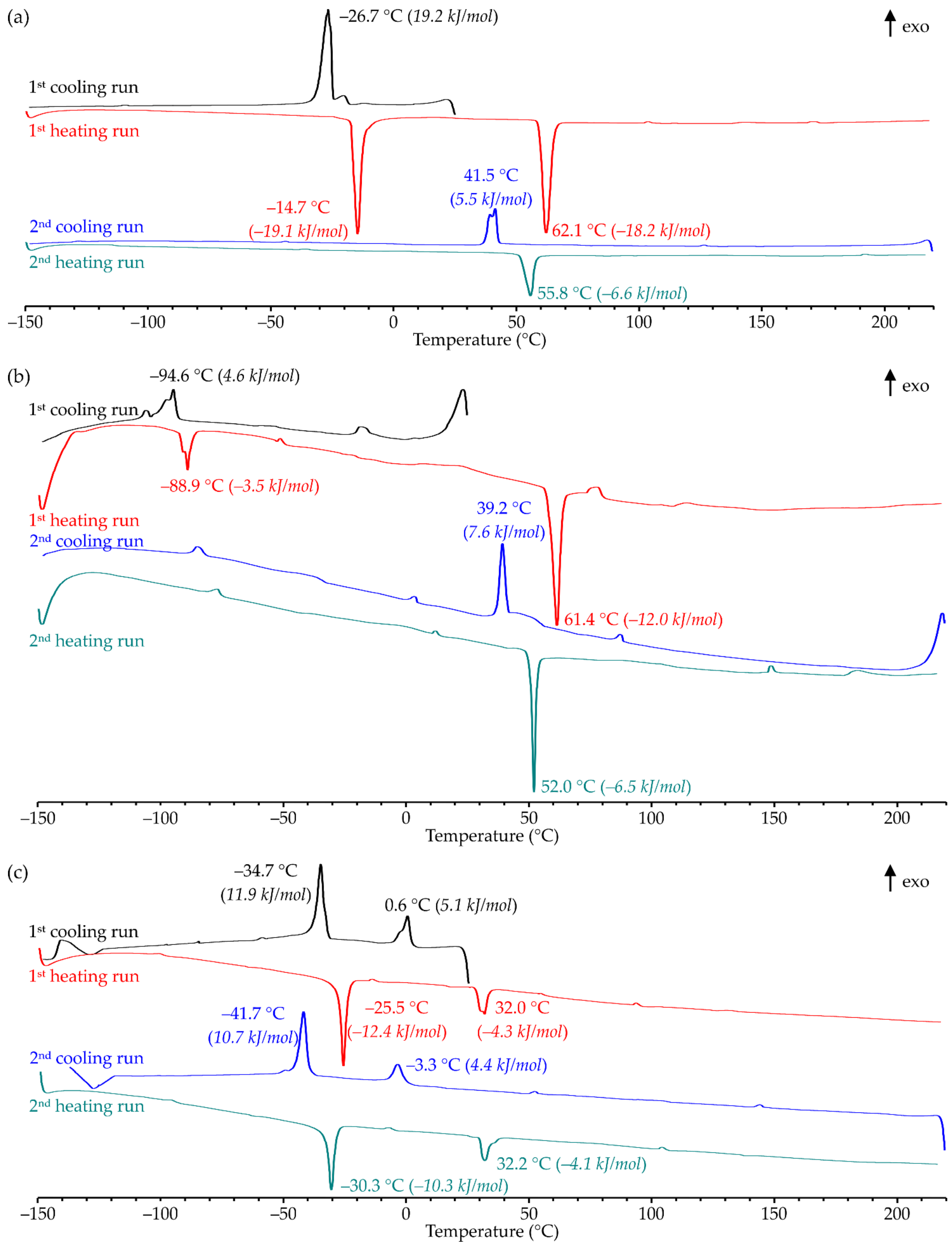

Figure S25. DSC traces of the pristine samples of: (a) ZnDnPP, (b) ZnDBP (sample heated to 60 °C during the last step of the synthesis), and (c) ZnBEHP. The measurements were carried out in the following order of runs: 1st cooling→1st heating→2nd cooling→2nd heating.

corresponding author: maciej.debowski@pw.edu.pl

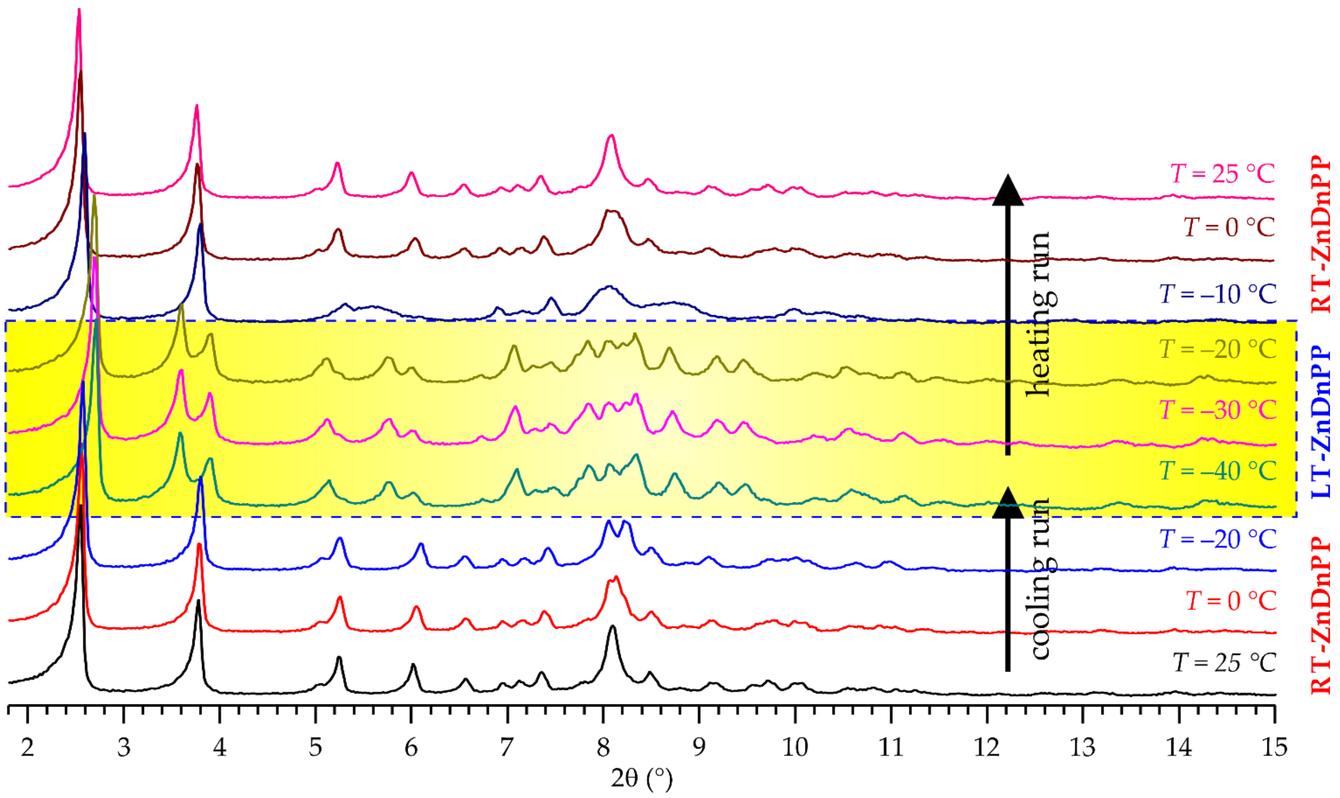

Figure S26. VT-PXRD traces of ZnDnPP recorded below room temperature, during cooling and subsequent heating steps. The measurements were conducted on an instrument equipped with Ag X-ray source (λ = 0.561 Å). Abbreviations: RT-ZnDnPP – room temperature ZnDnPP crystalline phase existing above −10 – −20 °C, LT-ZnDnPP – low temperature ZnDnPP crystalline phase forming below −10 – −20 °C. The diffractograms are normalized between 0 and 1.

corresponding author: maciej.debowski@pw.edu.pl

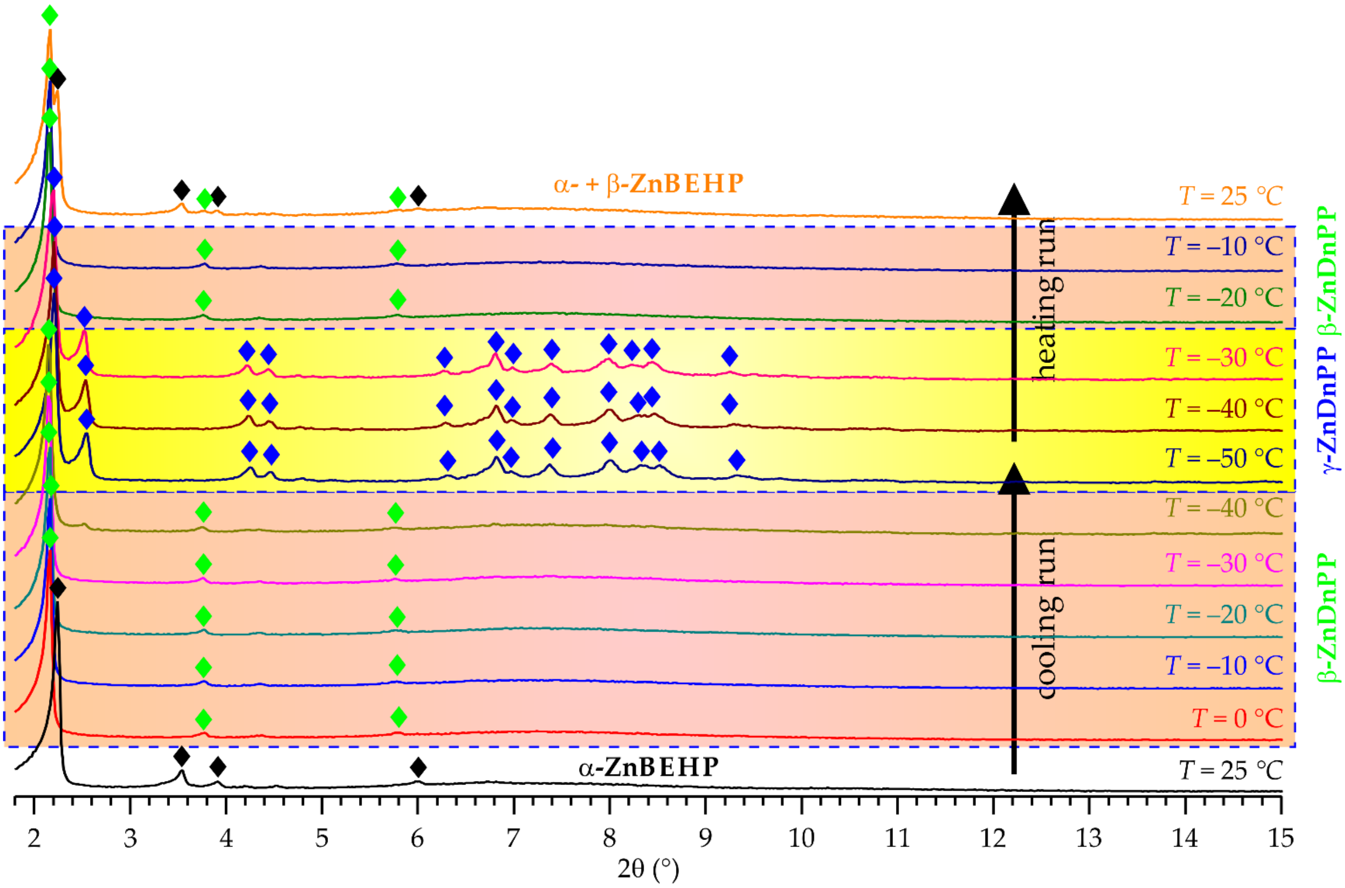

Figure S27. VT-PXRD patterns of ZnBEHP recorded below room temperature, during cooling and subsequent heating steps. Main reflections from different crystalline phases of ZnBEHP are marked with symbols: ♦ α-ZnBEHP (the main crystalline phase at room temperature and above it), ♦ β-ZnBEHP, and ♦ γ-ZnBEHP (the main crystalline phase existing at the lowest temperatures). The measurements were conducted on an instrument equipped with Ag X-ray source (λ = 0.561 Å). The diffractograms are normalized between 0 and 1.

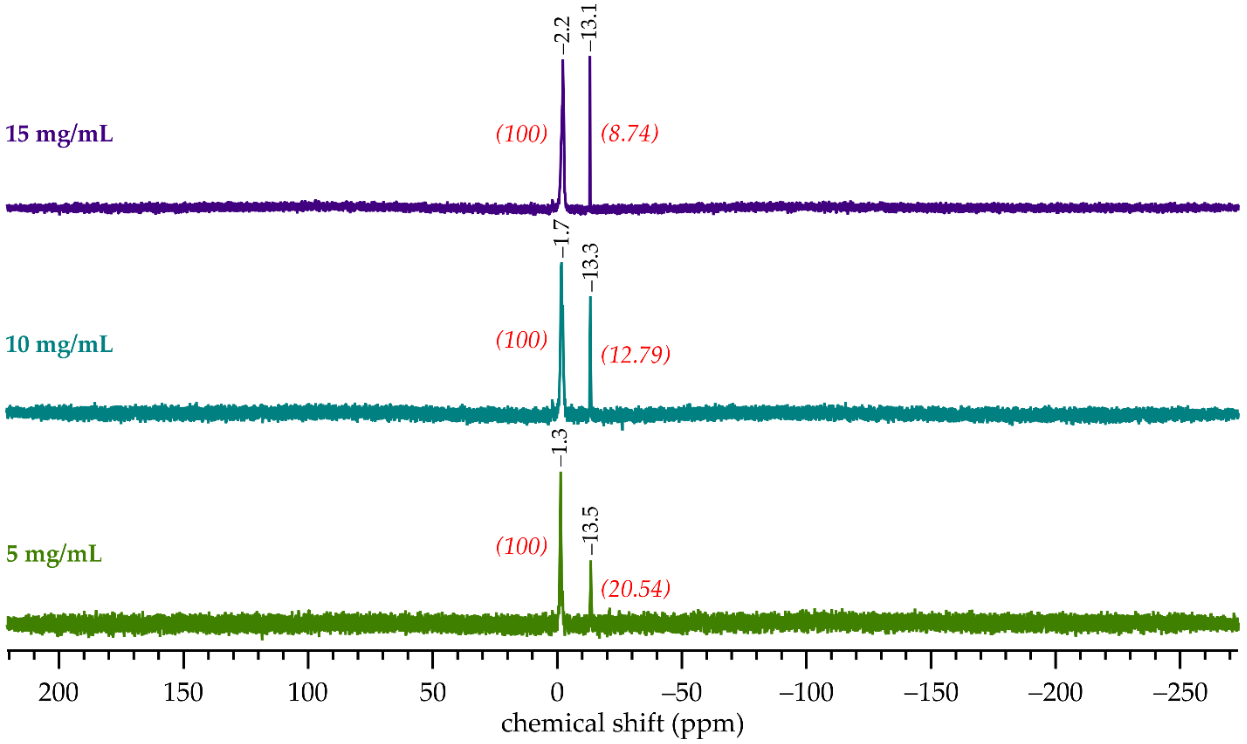

Figure S28. ^{31}P NMR spectra of ZnDBP solutions in $CDCl_3$ recorded at room temperature for different sample's mass concentrations (5–15 mg/mL). Relative peak integral for each NMR signal is given in parenthesis.

corresponding author: maciej.debowski@pw.edu.pl

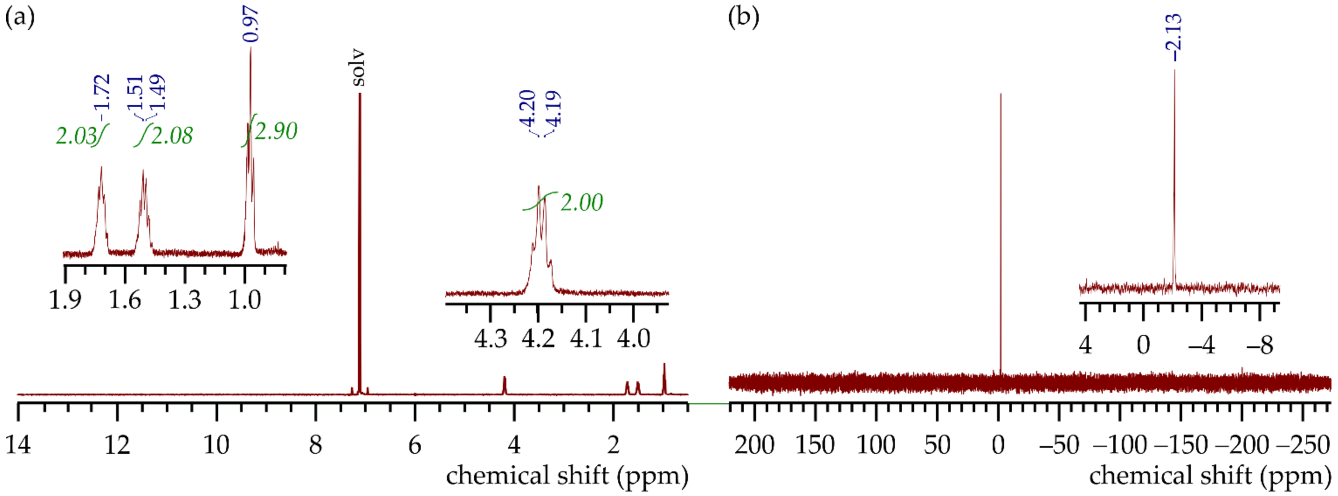

Figure S29. (a) ^{1}H and (b) ^{31}P NMR spectra of ZnDBP solution in benzene-d_6 (mass concentration of 1 mg/mL) recorded at room temperature. Abbreviations: solv – solvent residual peak.

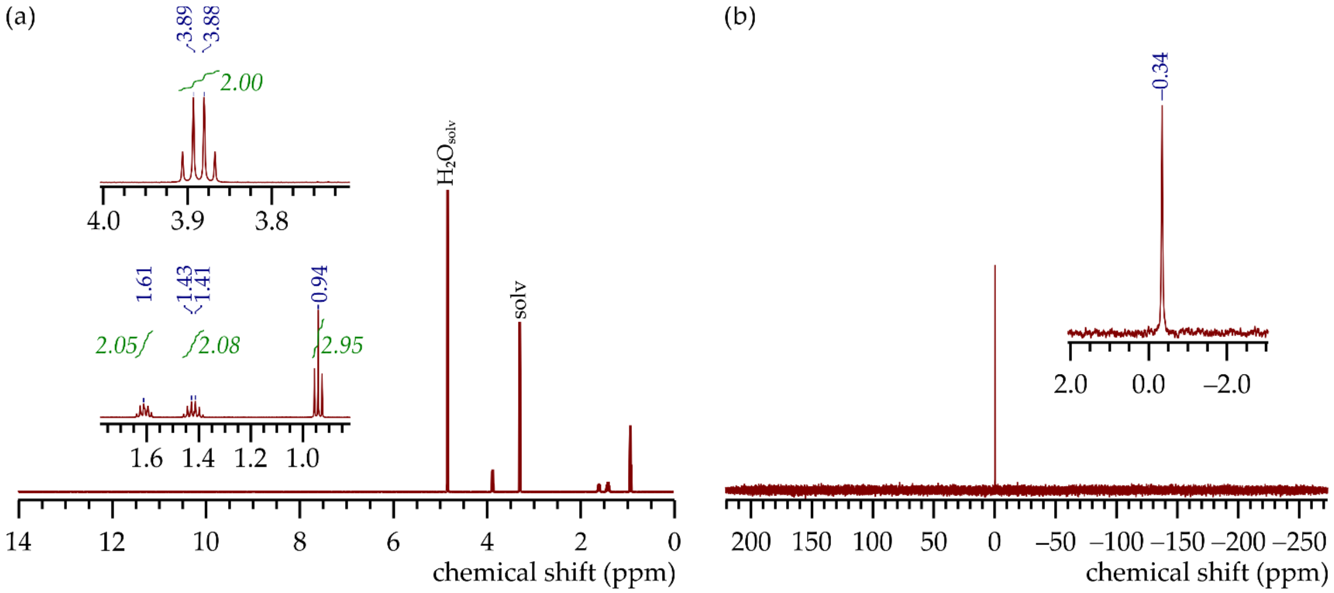

Figure S30. (a) ^{1}H and (b) ^{31}P NMR spectra of ZnDBP solution in methanol-d_4 (mass concentration of 1 mg/mL) recorded at room temperature. Abbreviations: solv – solvent residual peak, H_2O_{solv} – water present in the solvent.

corresponding author: maciej.debowski@pw.edu.pl

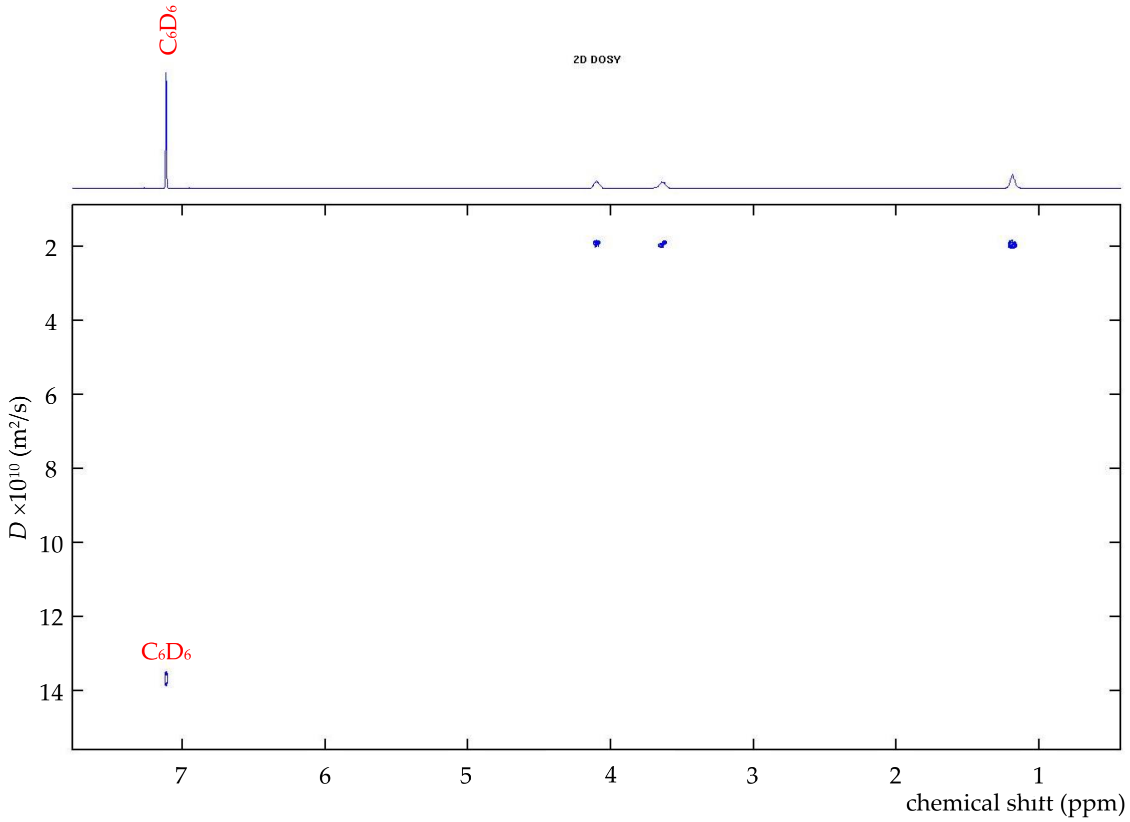

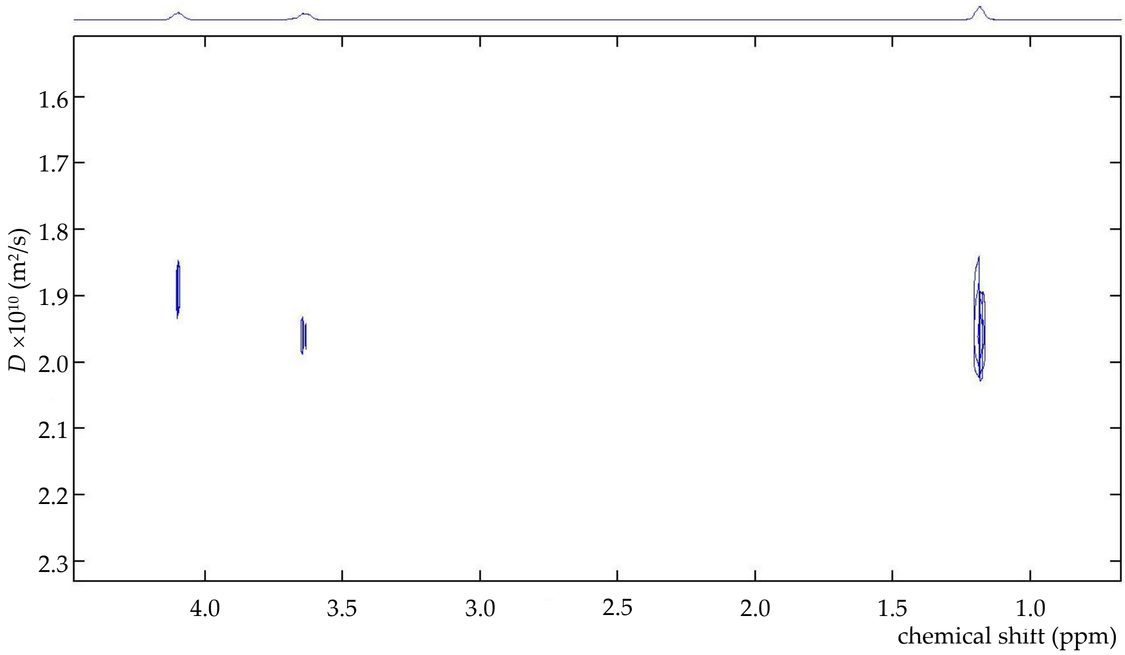

Figure S31. ^{1}H DOSY NMR spectrum of the ZnDMP–*co*–ZnDEP copolymer (50 mol% of ZnDMP monomeric units) dissolved in C_6D_6 (mass concentration of 5 mg/mL): full spectrum (top) and the copolymer's peaks area (bottom). The measurements were conducted at 25 °C.

corresponding author: maciej.debowski@pw.edu.pl

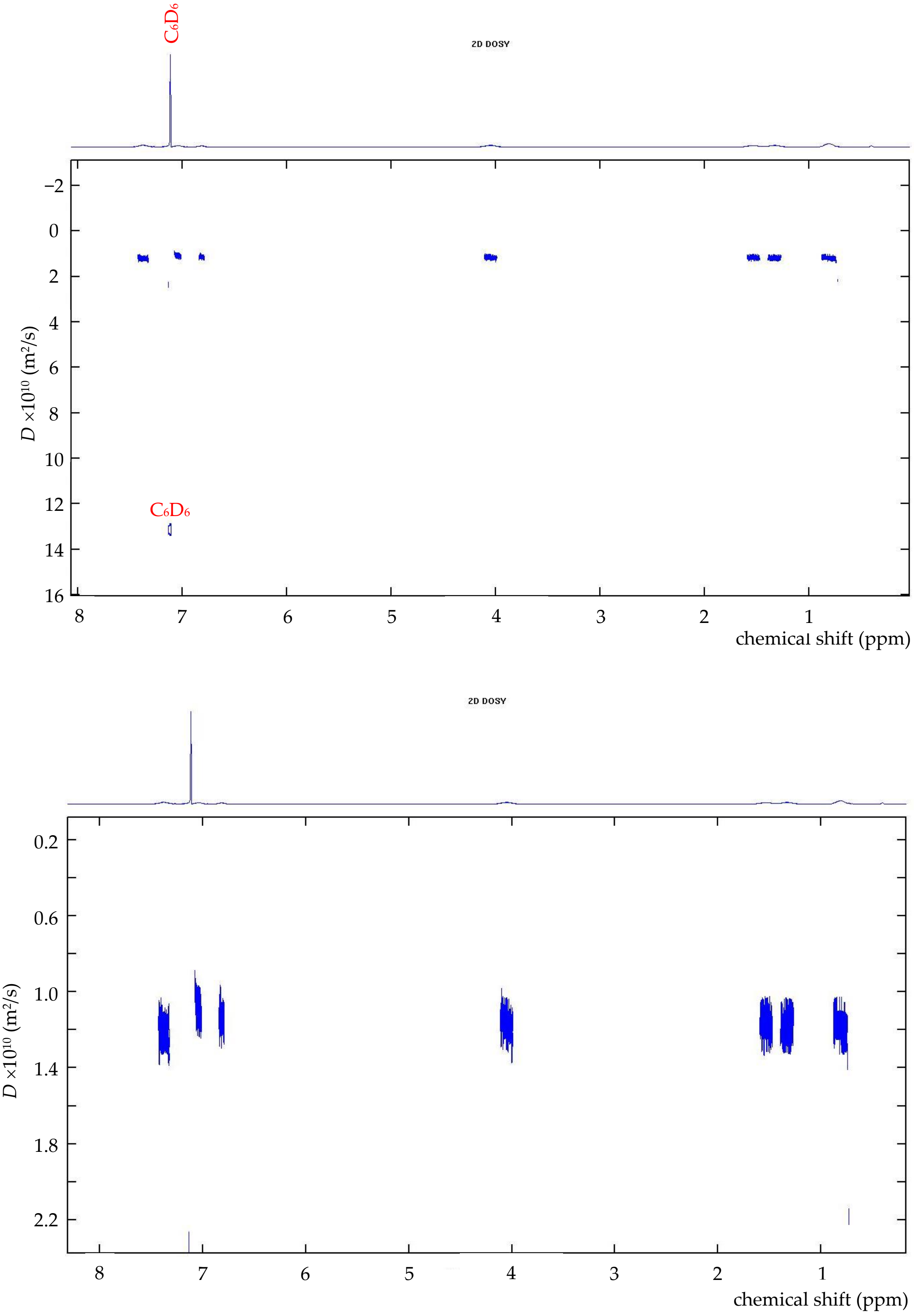

Figure S32. ^{1}H DOSY NMR spectrum of the ZnDBP–*co*–ZnDPhP copolymer (50 mol% of ZnDPhP monomeric units) dissolved in C_6D_6 (mass concentration of 5 mg/mL): full spectrum (top) and the copolymer's peaks area (bottom). The measurements were conducted at 25 °C.

corresponding author: maciej.debowski@pw.edu.pl

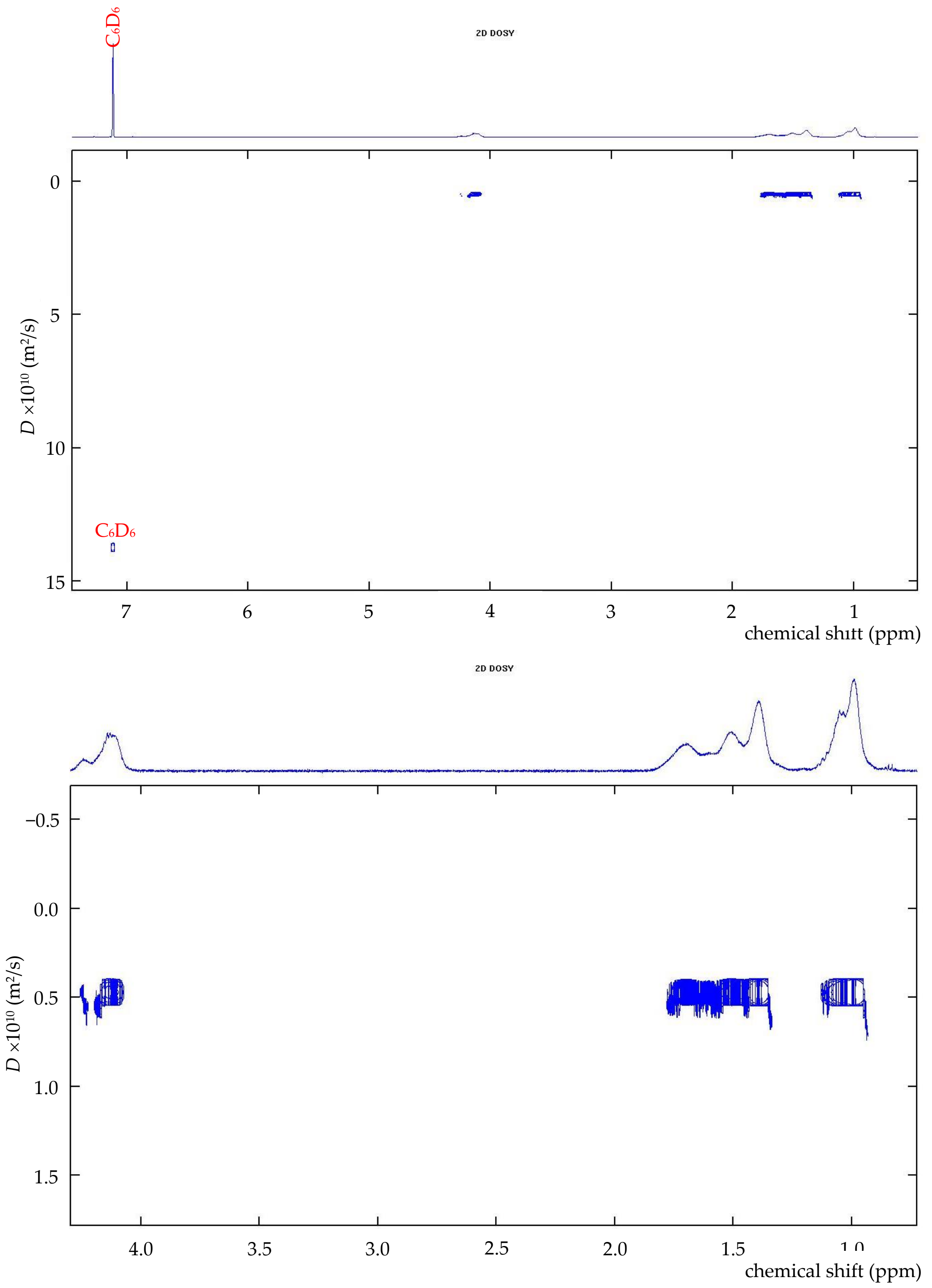

Figure S33. ^{1}H DOSY NMR spectrum of the ZnDBP–*co*–ZnBEHP copolymer (50 mol% of ZnDBP monomeric units) dissolved in C_6D_6 (mass concentration of 5 mg/mL): full spectrum (top) and the copolymer's peaks area (bottom). The measurements were conducted at 25 °C.

corresponding author: maciej.debowski@pw.edu.pl

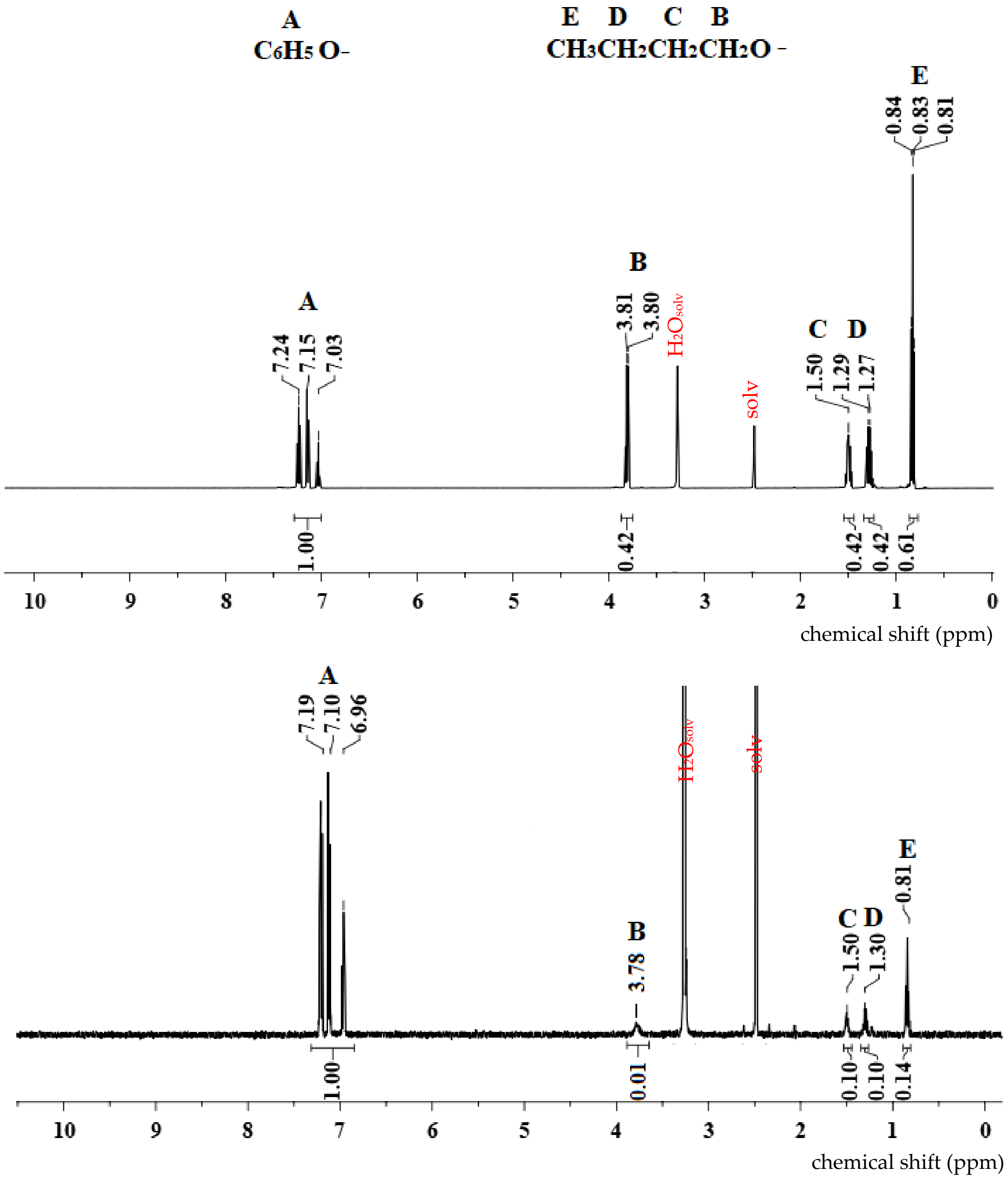

Figure S34. ^{1}H NMR spectra of different fractions of the ZnDBP-*co*-ZnDPhP copolymer (50 mol% of ZnDPhP monomeric units): fraction soluble (top) and insoluble (bottom) in cyclohexane. Spectra recorded in DMSO-d_6 at 25 °C. Abbreviations: solv – residual solvent peak, H_2O_{solv} – water present in the solvent.

corresponding author: maciej.debowski@pw.edu.pl

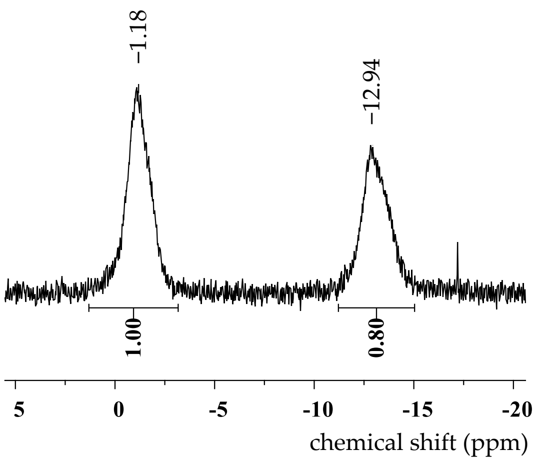

Figure S35. ^{31}P NMR spectrum of the ZnDBP-*co*-ZnDPhP copolymer (50 mol% of ZnDPhP monomeric units) recorded in C_6D_6 at 25 °C.

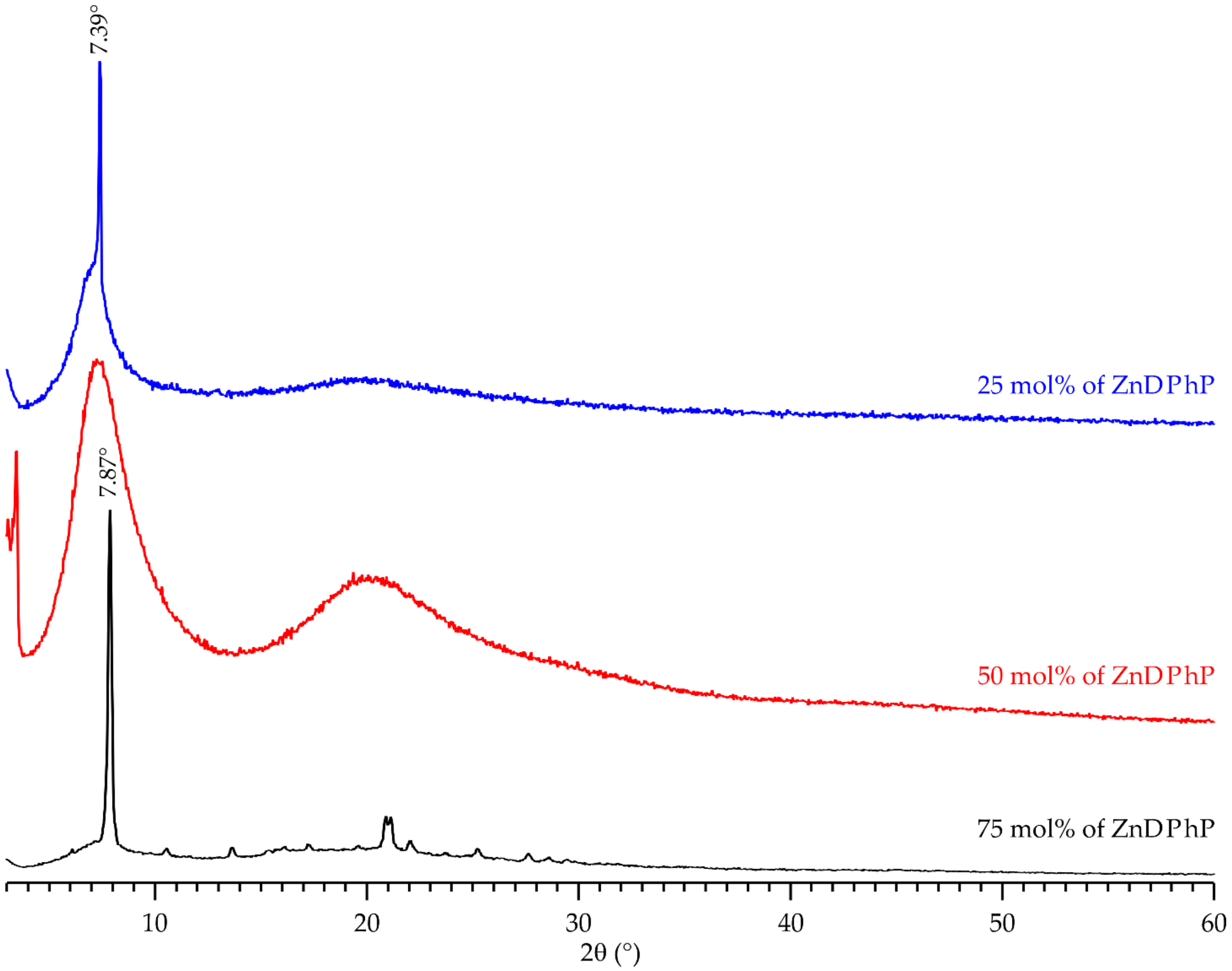

Figure S36. PXRD patterns of the ZnDBP-*co*-ZnDPhP copolymers containing different amounts of aromatic monomeric units: 25 mol% (top), 50 mol% (middle), and 75 mol% (bottom). The measurements were conducted at 25 °C.

corresponding author: maciej.debowski@pw.edu.pl

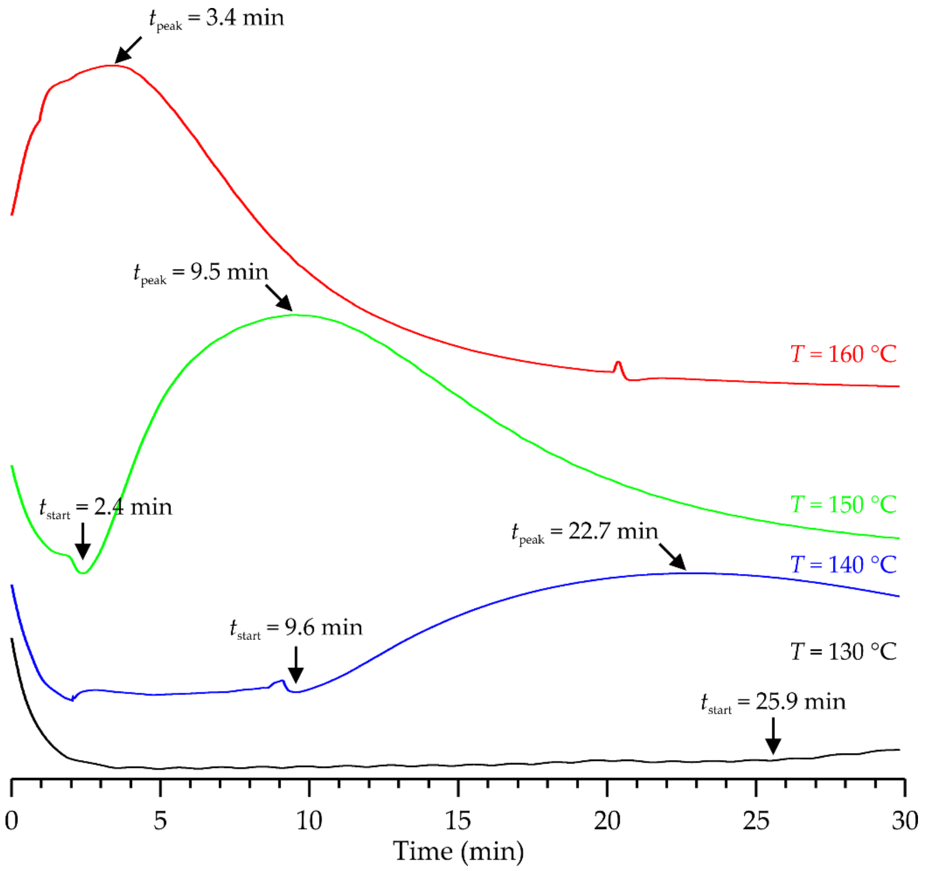

Figure S37. DSC traces recorded during isothermal heating of the ZnDEP/BADGE composition (20 wt% of ZnDEP load) at different temperatures. The time points of the beginning and maximum of the exothermic peak (t_{start} and t_{peak}, respectively) are marked with arrows.

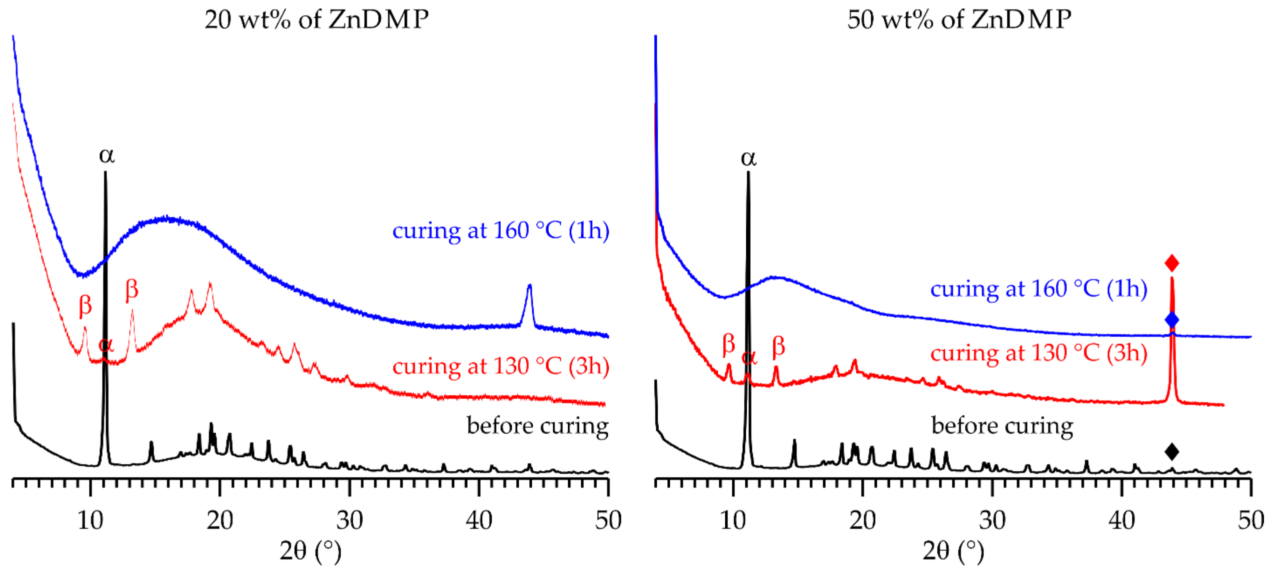

Figure S38. PXRD patterns of the ZnDMP/BADGE compositions containing 20 wt% (left) and 50 wt% (right) of the filler/catalyst: before curing (black line), and cured for 3h at 130 °C (red line) or for 1h at 160 °C (blue line). Symbol ♦ indicates a reflection from diamond powder used as an internal standard. Some reflections of α-ZnDMP or β-ZnDMP are marked with α and β symbols, respectively. The diffractograms are normalized between 0 and 1.

corresponding author: maciej.debowski@pw.edu.pl

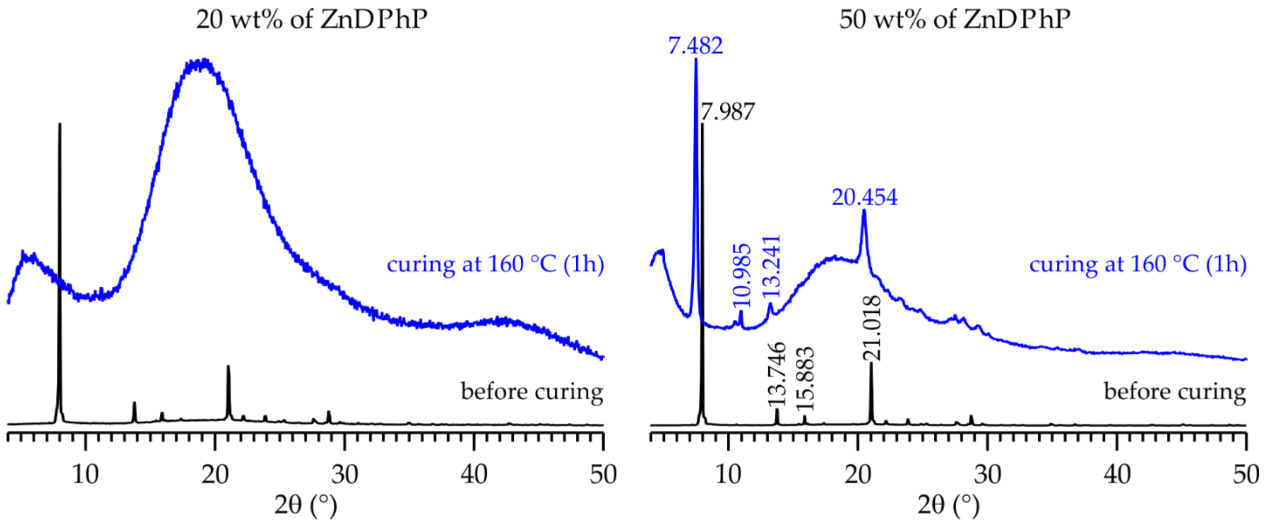

Figure S39. PXRD patterns of the ZnDPhP/BADGE compositions containing 20 wt% (left) and 50 wt% (right) of the filler/catalyst: before curing (black line), and cured for 1h at 160 °C (blue line). The diffractograms are normalized between 0 and 1.

corresponding author: maciej.debowski@pw.edu.pl
